# Synthesis and evaluation of coumarin derivatives on antioxidative, tyrosinase inhibitory activities, melanogenesis, and in silico investigations

**DOI:** 10.1038/s41598-024-54665-x

**Published:** 2024-03-06

**Authors:** Kasemsiri Chandarajoti, Jiraporn Kara, Paptawan Suwanhom, Teerapat Nualnoi, Jindaporn Puripattanavong, Vannajan Sanghiran Lee, Varomyalin Tipmanee, Luelak Lomlim

**Affiliations:** 1 Department of Pharmaceutical Chemistry, Faculty of Pharmaceutical Sciences, Prince of Songkla University, Hat Yai, Songkhla, 90112 Thailand; 2 Drug Delivery System Excellence Center, Faculty of Pharmaceutical Sciences, Prince of Songkla University, Hat-Yai, Songkhla, 90112 Thailand; 3 Phytomedicine and Pharmaceutical Biotechnology Excellent Center (PPBEC), Faculty of Pharmaceutical Sciences, Prince of Songkla University, Songkhla, 90112 Thailand; 4 Department of Pharmaceutical Technology, Faculty of Pharmaceutical Sciences, Prince of Songkla University, Hat Yai, Songkhla, 90112 Thailand; 5 Department of Pharmacognosy and Pharmaceutical Botany, Faculty of Pharmaceutical Sciences, Prince of Songkla University, Hat Yai, Songkhla, 90112 Thailand; 6 Department of Chemistry, Faculty of Science, University of Malaya, 50603 Kuala Lumpur, Malaysia; 7 Department of Biomedical Sciences and Biomedical Engineering, Faculty of Medicine, Prince of Songkla University, Songkhla, 90112 Thailand

**Keywords:** Drug discovery, Chemistry

## Abstract

New coumarin derivatives were designed using a 2-(2-oxo-2*H*-chromen-4-yl)acetic acid scaffold conjugated with amino acid esters or tyramine. The anti-tyrosinase and anti-lipid peroxidation activities of the synthesized compounds were investigated. Coumarin derivatives **7**,**9**, **11**–**13**, **15**–**18** showed strong anti-lipid peroxidation activity. Compound **13** exhibited uncompetitive tyrosinase inhibitory activity with an IC_50_ value of 68.86 µM. Compound **14** (% activity = 123.41) showed stronger tyrosinase activating activity than 8-methoxypsolaren (8-MOP, % activity = 109.46). In silico studies revealed different poses between the inhibitors and activators near the tyrosinase catalytic site. Compounds **13** (25–50 μM) and **14** (25–100 μM) did not show cytotoxicity against B16F10 cells. In contrast to the tyrosinase inhibition assay, compound **13** (50 μM) suppressed melanogenesis in B16F10 cells with two times higher potency than KA (100 μM). Compound **14** at 100 μM showed melanogenesis enhancement in B16F10 cells in a dose-dependent manner, however, inferior to the 8-MOP. Based on the findings, compound **13** and **14** offer potential for development as skin-lightening agents and vitiligo therapy agents, respectively.

## Introduction

Melanin is a pigment that controls the color of the skin and hair and is crucial for shielding the skin from ultraviolet (UV) radiation. Melanin is produced by the melanocytes in the epidermal layer. Skin disorders and aesthetic issues might result from an unbalanced melanogenesis process. Melasma and skin discoloration are caused by hyperpigmentation^1^. UV exposure is also the leading cause of skin aging due to reactive oxygen species (ROS) that can oxidize skin structure and suppress procollagen production, causing collagen destruction and wrinkles^2^.

Tyrosinase (E.C. 1.14.18.1) is the enzyme that triggers the rate-limiting step in melanin synthesis^3,4^. This copper-containing metalloenzyme is a glycoprotein located on the surface of melanosomes. The catalytic region of tyrosinase consists of histidine residues bound with copper ions to form the catalytic region for its function^1^. Tyrosinase inhibitors such as kojic acid (KA), arbutin, tropolone, and 1-phenyl-2-thiourea (PTU) are used in cosmeceuticals currently available on the market. However, these tyrosinase inhibitors are still far from ideal for cosmeceutical development because of stability issues, low potency, a lack of clinical efficacy, and toxicity. As a result, new tyrosinase inhibitors with higher potency and reduced toxicity are still needed^5^.

Natural and synthetic coumarin derivatives have been reported as tyrosinase inhibitors^6–10^. Novel compounds with higher efficacy were developed by conjugating molecules of protocatechuic acid, cinnamic acid, KA, and caffeic acid with amino acids such as phenylalanine, tryptophan, tyrosine, and the structurally related substance tyramine^11–14^. 2-(2-oxo-2*H*-chromen-4-yl)acetic acid is an advantageous scaffold for drug discovery. It has been exploited as a structural motif for the design of anti-bacterial agents, anti-cancer agents, antioxidants, and acetylcholinesterase inhibitors^15–18^. In this study, the 2-(2-oxo-2*H*-chromen-4-yl)acetic acid scaffold was used for designing coumarin-amino acid ester or tyramine conjugates as tyrosinase inhibitors. In this work, new coumarin compounds were designed, synthesized, and investigated for their in vitro lipid peroxidation inhibitory activity, tyrosinase inhibitory activity, and melanogenesis. We aimed to synthesize the tyrosinase inhibitors by varying the substituents at R_3_ and R_4_ of the coumarin ring. Interestingly, certain substituents showed activation activity on mushroom tyrosinase. By using molecular docking, the inhibitor's and activator’s interactions with the tyrosinase enzyme were simulated. Additionally, we investigated the melanin content in the melanoma cells. The results showed that the synthetic inhibitor and activator affected melanogenesis in the cells. These molecules could be considered potential agents for the treatment of skin pigmentation disorders and cosmeceuticals.

## Results

### Chemistry

**Figure 1:**
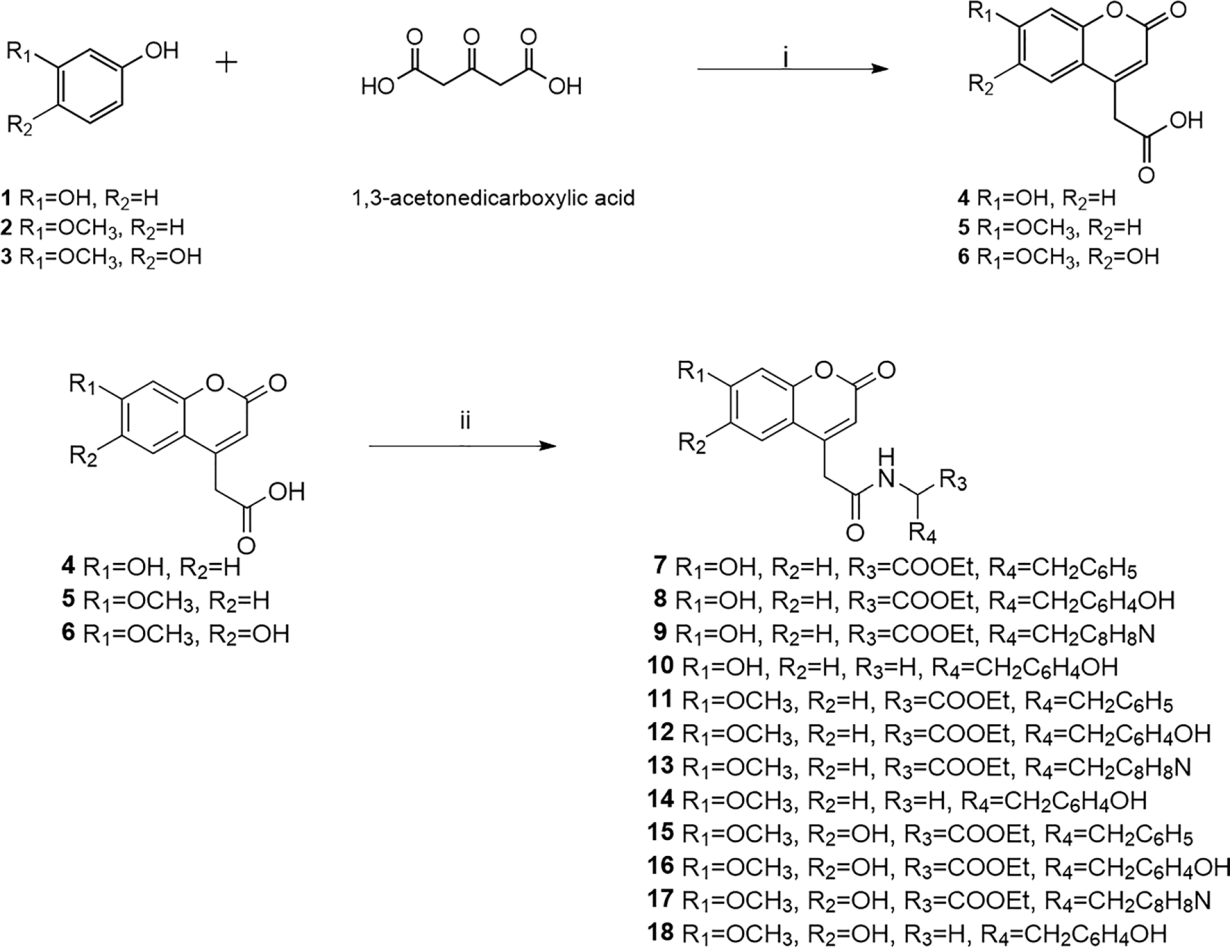
Synthesis pathway of coumarin derivatives (i) H_2_SO_4_, MeOH, 0 °C; (ii) corresponding amino acid ethyl ester or tyramine, EDCI, DMAP, DIEA, DMF, r.t.

Twelve coumarin derivatives were designed and prepared via 2-step synthesis. The first step was the formation of the 2-(2-oxo-2*H*-chromen-4-yl)acetic acid scaffolds (**4**–**6**), and the final step was the coupling of the scaffold with the corresponding amino acid esters or tyramine to yield the desired products (**7**–**18**), as shown in Fig. 1. The chemical structure of the intermediates and final products was confirmed with standard spectroscopic methods and high-resolution mass spectrometry. The bulk purity of the products was confirmed using high-performance liquid chromatography (HPLC).

### Lipid peroxidation inhibitory activity

Lipid peroxidation is a process in which oxidants such as free radicals or nonradical species attack unsaturated lipids^19^. Malondialdehyde (MDA), a minor product of lipid peroxidation, has been employed as a biomarker for lipid peroxidation. In the thiobarbituric acid (TBA) test, nine coumarin derivatives (**7**, **9**, **11–13**, **15–18**) showed remarkable anti-lipid peroxidation efficacy (%inhibition 63.40 ± 0.89 to 98.66 ± 1.57) when compared to butylated hydroxytoluene (BHT, % inhibition 62.90 ± 3.56) at a concentration of 1 mg/mL (Table 1). Compound **7** (% inhibition 98.66 ± 1.57) exhibited the highest anti-lipid peroxidation. Excellent anti-lipid peroxidation activity was also observed in compounds **11**, **15**, and **17**, which exhibit %inhibition > 85. The phenylalanine ethyl ester derivatives (**7**, **11**, **15**) generally showed excellent activity, while the tyramine derivatives (**10**, **14**, **18**) exhibited lower potency. Tyrosine ethyl ester derivatives (**8**, **12**, **16**) demonstrated stronger activity than the tyramine-derived compounds (**10**, **14**, **18**).

**Table 1:** Lipid peroxidation and mushroom tyrosinase inhibitory activity of the coumarin derivatives.


Cpd	R_1_	R_2_	R_3_	R_4_	Lipid peroxidation	Mushroom tyrosinase		Activity
					%Inhibition ± S.D.^a^	%Activity ± S.D.^b^	IC_50_ ± S.D. (µM)	
**7**	OH	H	COOEt	Bn	98.66 ± 1.57	86.22 ± 10.50	> 100	Inhibitor
**8**	OH	H	COOEt	4-OH Bn	44.32 ± 4.16	106.60 ± 9.89	n.d	Activator
**9**	OH	H	COOEt	CH_2_-Indole	77.57 ± 4.11	98.28 ± 2.55	> 100	Inhibitor
**10**	OH	H	H	4-OH Bn	27.51 ± 2.78	120.57 ± 7.51	n.d	Activator
**11**	OCH_3_	H	COOEt	Bn	87.68 ± 1.09	81.31 ± 3.72	> 100	Inhibitor
**12**	OCH_3_	H	COOEt	4-OH Bn	77.84 ± 3.25	112.58 ± 8.16	n.d	Activator
**13**	OCH_3_	H	COOEt	CH_2_-Indole	67.88 ± 5.48	51.11 ± 9.45	68.86 ± 4.11	Inhibitor
**14**	OCH_3_	H	H	4-OH Bn	26.66 ± 1.67	123.41 ± 13.37	n.d	Activator
**15**	OCH_3_	OH	COOEt	Bn	98.06 ± 0.54	90.79 ± 12.39	> 100	Inhibitor
**16**	OCH_3_	OH	COOEt	4-OH Bn	79.31 ± 2.77	100.12 ± 3.13	n.d	Activator
**17**	OCH_3_	OH	COOEt	CH_2_-Indole	91.84 ± 5.39	75.91 ± 8.95	> 100	Inhibitor
**18**	OCH_3_	OH	H	4-OH Bn	63.40 ± 0.89	114.97 ± 3.87	n.d	Activator
**BHT**	–	–	–	–	62.90 ± 3.56	n.d	n.d	N.A
**KA**	–	–	–	–	n.d	4.75 ± 1.04	17.84 ± 0.61	Inhibitor
**8-MOP**	–	–	–	–	n.d	109.46 ± 3.34	n.d	Activator

^a^%lipid peroxidation inhibition at 1 mg/ml of tested compound.

^b^%mushroom tyrosinase activity at 100 µM of tested compound.

*BHT* butylated hydroxytoluene, *KA* kojic acid, *8-MOP* 8-methoxypsoralen, *n.d.* not determined, *N.A.* not applicable.

### Tyrosinase inhibition assay

The synthesized compounds were screened for their effect on mushroom tyrosinase using KA as a reference tyrosinase inhibitor and 8-methoxy-psoralen (8-MOP) as a reference tyrosinase activator. Table 1 displays the % activity of mushroom tyrosinase following incubation with the studied compounds; when the % activity value is less than 100, the molecule is considered an inhibitor, and when it is greater than 100, the compound is considered an activator^20^.

On mushroom tyrosinase, the coumarin derivatives exhibited different effects. Tyrosinase inhibitory action was observed in the phenylalanine ethyl ester and tryptophan ethyl ester derivatives (**7**, **11**, **15,** and **9**, **13**, **17**, respectively). In comparison to KA, the compounds showed mild tyrosinase inhibitory activity. Compound **13** (% activity = 51.11 ± 9.45, IC_50_ = 68.86 ± 4.11 µM) was the most effective tyrosinase inhibitor among these coumarin derivatives. Other compounds that showed low potency (less than 25% inhibition at 100 µM) were not further investigated. Tyrosinase activators include tyrosine ethyl ester and tyramine derivatives (**8**, **12**, **16,** and **10**, **14**, **18**). These compounds (except **16**) have a stronger activating effect on mushroom tyrosinase (% activity ranging from 106.60 ± 9.89 to 123.41 ± 13.37) than 8-MOP (% activity = 101.05 ± 6.18).

### Enzyme kinetic study

**Figure 2:**
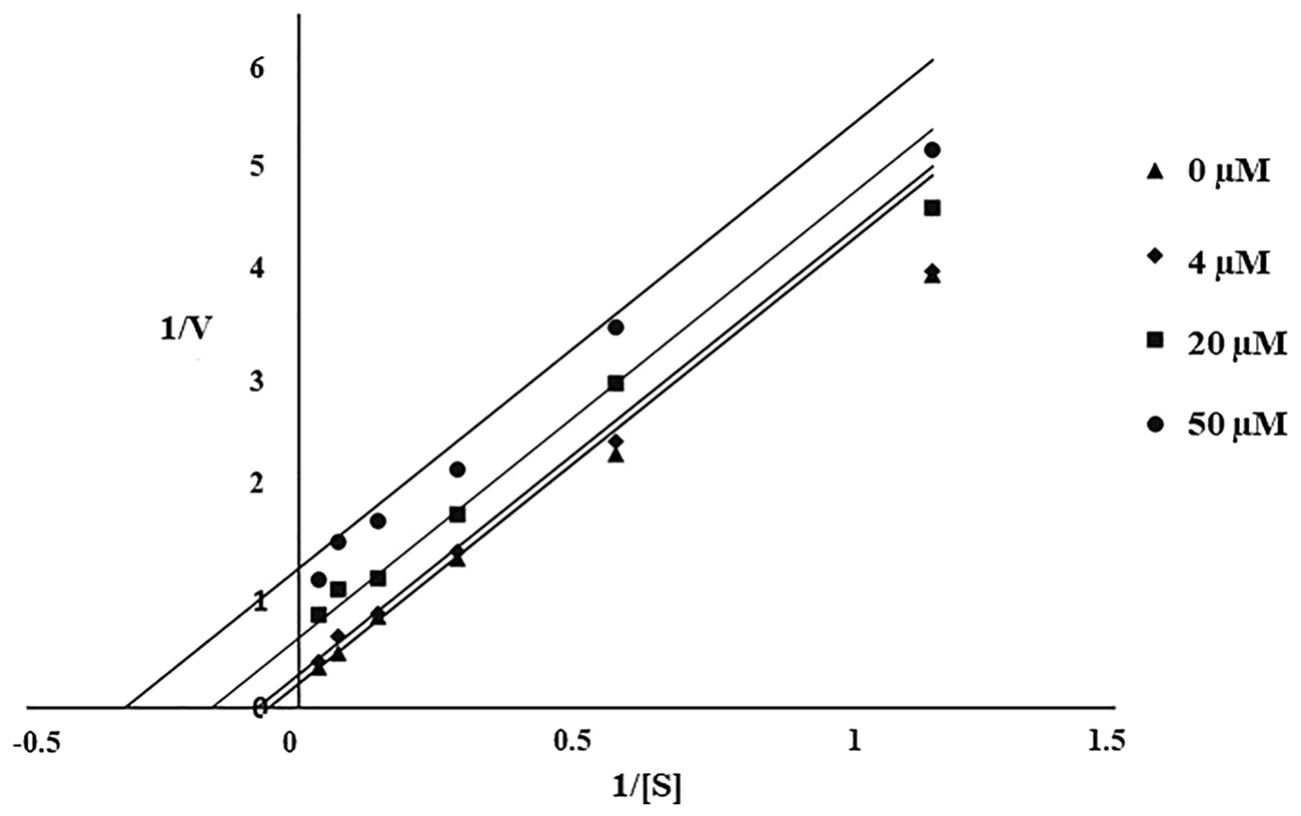
Lineweaver–Burk Plot of compound **13**.

Double-reciprocal plots were used to investigate the mechanisms of mushroom tyrosinase inhibition by compound **13** (Fig. 2). Plotting 1/V vs. 1/[S] yielded parallel straight lines with equal slopes, indicating uncompetitive inhibition.

### Molecular docking

**Figure 3:**
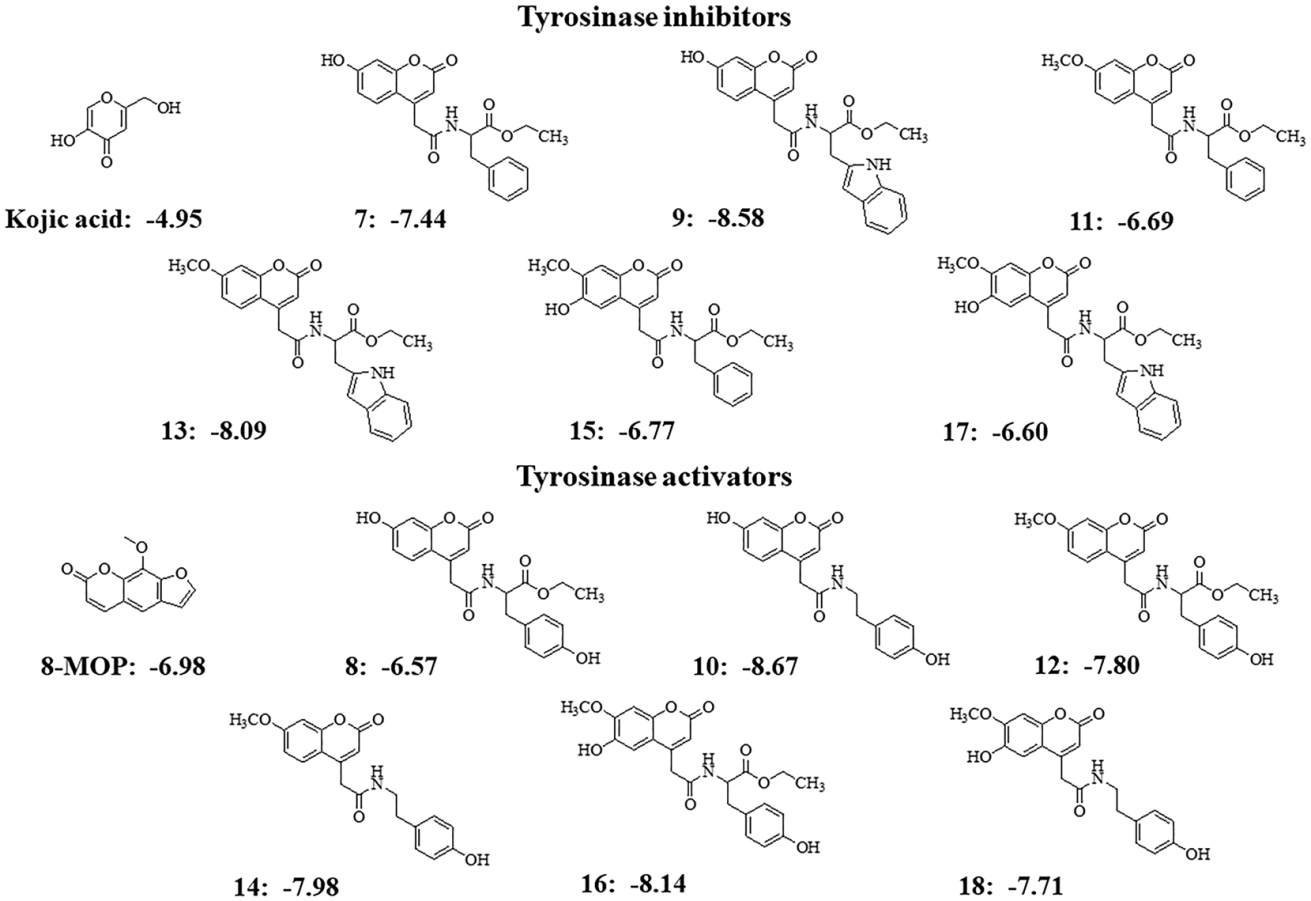
Predictive docking score (ΔG in kcal/mol) of synthesis compounds with mushroom tyrosinase (PDB ID: 2Y9X).

Tyrosinase enzyme is made up of two identical H and two identical L subunits. The two copper ions (Cu^2+^), each of them paired with three histidine residues, are located at the active site of the H subunit. The first Cu^2+^ was linked to His61, His84, and His95, while the second Cu^2+^ was linked to His259, His263, and His296^21^. The docking behavior of the synthesized compounds (**7**–**18**) and the reference compounds (KA, 8-MOP, and tropolone) with the mushroom tyrosinase protein (PDB ID: 2Y9X) was evaluated using the docking score (kcal/mol) from AutoDock4^22^ as shown in Fig. 3.

**Figure 4:**
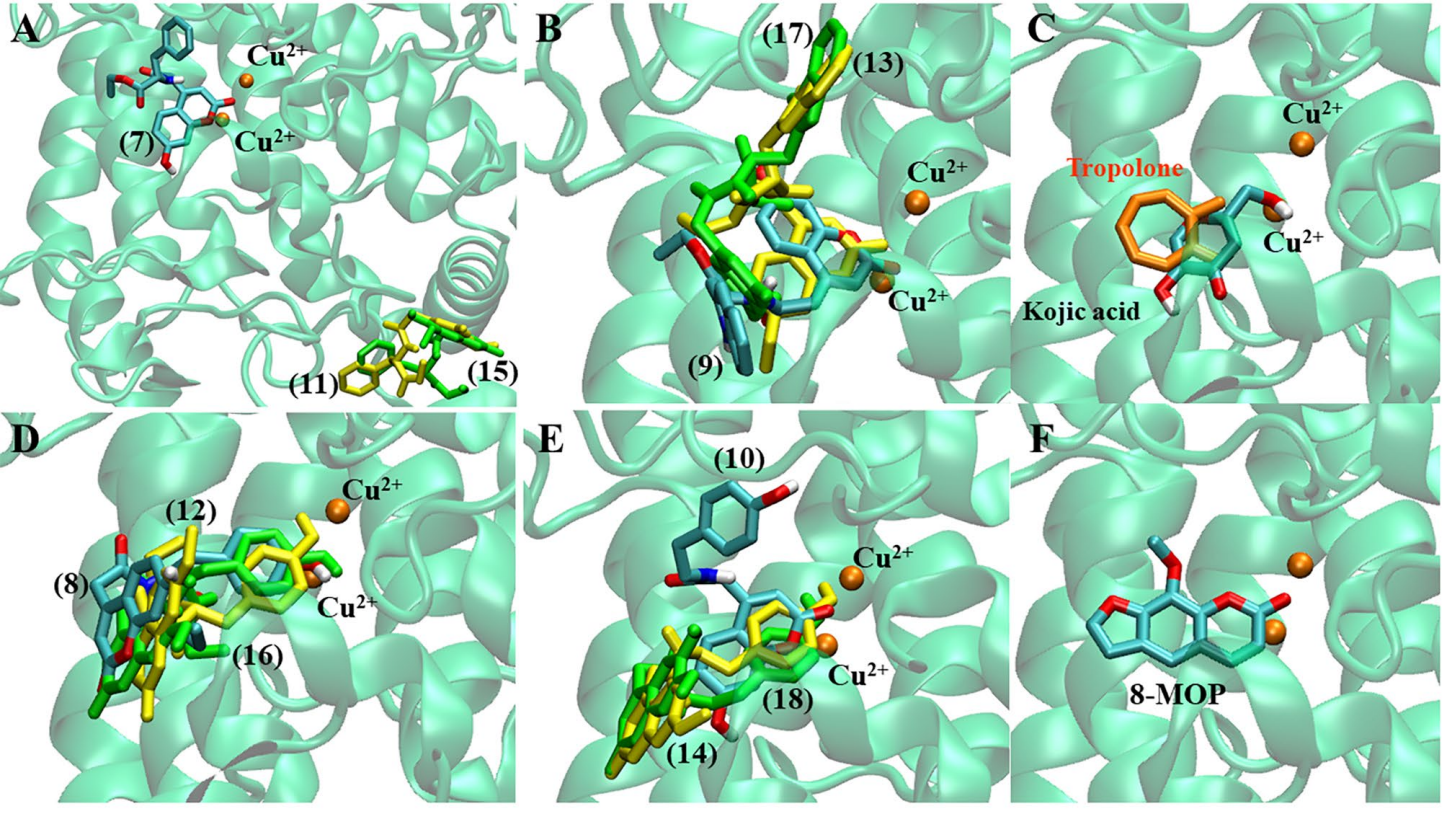
Predicted ligand conformation in a tyrosinase-bound state.

In Fig. 3, the synthesized compounds (**7**–**18**) were separated into two groups based on the coumarin substituent(s). Tyrosinase inhibitors included phenylalanine ethyl ester derivatives (**7**, **11**, **15**) and tryptophan ethyl ester derivatives (**9**, **13**, **17**), while tyrosine ethyl ester derivatives (**8**, **12**, **16**) and tyramine derivatives (**10**, **14**, **18**) acted as tyrosinase activators. Compounds **7**, **9**, **13**, and **17** were bound at the active site with different conformational poses and binding energy values, except compounds **11** and **15**, which were located far from the protein's pocket site (see Fig. 4A,B). The coumarin ring could bind with both copper ions in this group, the same as the KA and tropolone (Fig. 4C). Except for compound **10**, all activators (**8**, **10**, **12**, **14**, **16**, **18**) were well accommodated in the binding site, and their phenolic group of tyrosine or tyramine moiety interacted with the copper ions in the same manner as 8-MOP (Fig. 4D–F).

**Figure 5:**
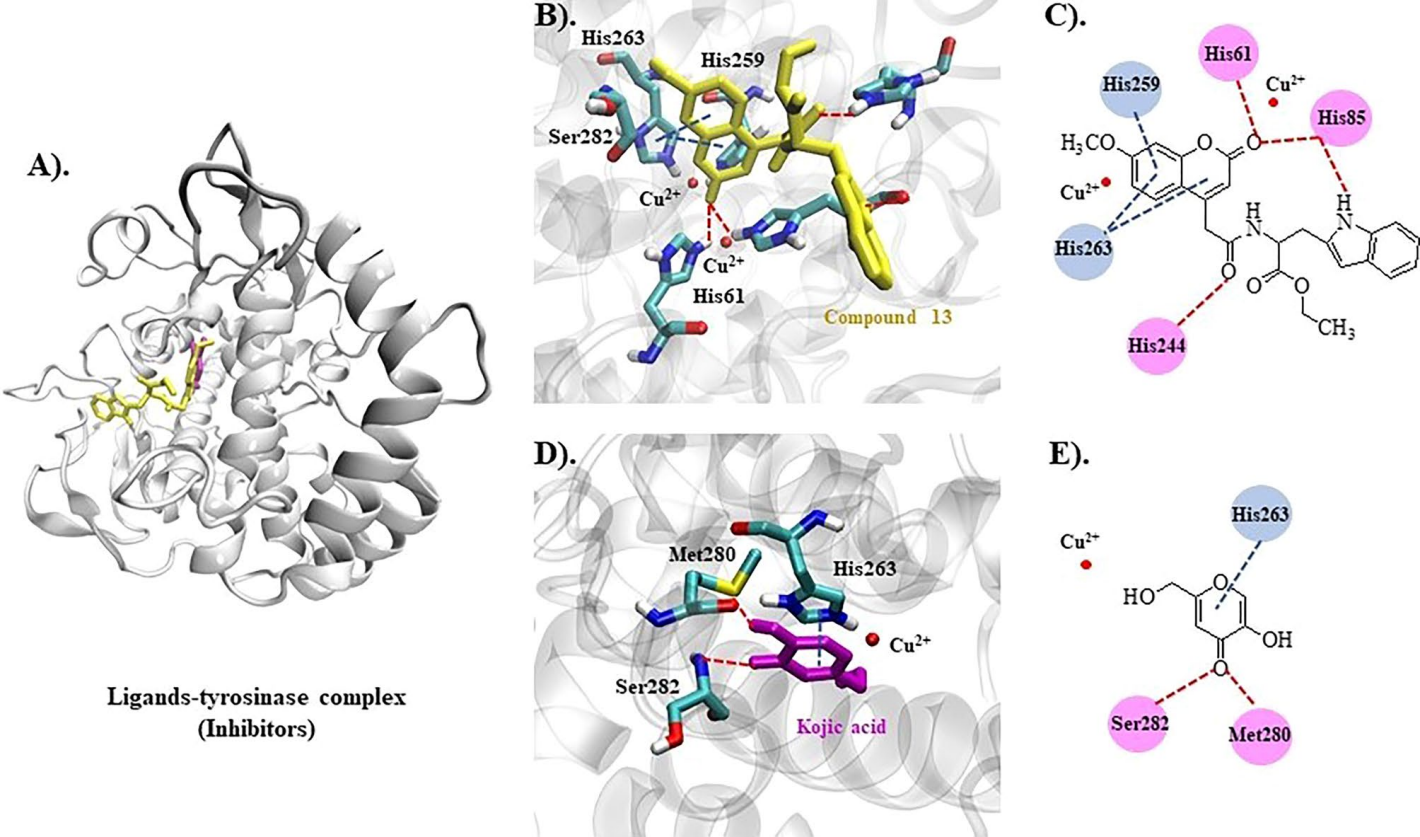
Inhibitors (compound **13** and KA) bound with the tyrosinase protein. (**A**) Molecular docking model of compound **13** (yellow) and KA (magenta) on the tyrosinase active site. Ligand interaction diagrams of compound **13** (**B**,**C**) and KA (**D**,**E**), involving the hydrogen bond and π–π interaction as respectively illustrated in red and blue.

Compounds **13** and **14** were found to be tyrosinase inhibitors and activators in vitro, respectively. As a result, these docking complexes were used for conformational analysis. The docking score of compound **13** (inhibitor, ΔG = − 8.09 kcal/mol) was lower than that of KA (ΔG = − 4.95 kcal/mol). Compound **13**'s carbonyl oxygen atom formed a hydrogen bond with His244. The coumarin ring interacted with His259 and His263 through π-π stacking. Carbonyl oxygen on the coumarin ring also forms hydrogen bonds with His61 and His85. In the case of the reference KA, the molecule has a π-π interaction with His263 and forms hydrogen bonds with Met280 and Ser282 (Fig. 5). According to the tyrosinase inhibitor's docking results, replacing the hydroxyl group at the C7-position with the methoxy group may reduce the predictive binding score, for example, in compounds **7** and **11**, as well as **9** and **13**. The lower binding energy caused by methoxy-substituted coumarin (compound **13** with respect to the compound **9**) may affect tyrosinase inhibitory efficacy because the coumarin ring remained close to the Cu^2+^ in both compounds **9** and **13**, and the coumarin ring can participate in the Cu^2+^-associated electron transfer process. In this case, it is possible that if the binding scores were similar, indicating that the compounds have the same preference for the site, it would not contribute significantly to inhibitory efficacy as long as the coumarin ring remained close to the Cu^2+^ ions (Fig. 5B). However, if the hydroxyl group was added to the C6 position along with the methoxy at the C7-position, as in compounds **13** and **17**, compound **17**'s docking score decreased, and the coumarin ring remained farther away from the Cu^2+^ than compound **13**. These suggested that the hydroxyl group at the C6-position could reduce inhibitory efficacy by inducing the binding pose, resulting in a greater distance between the coumarin ring and the Cu^2+^.

**Figure 6:**
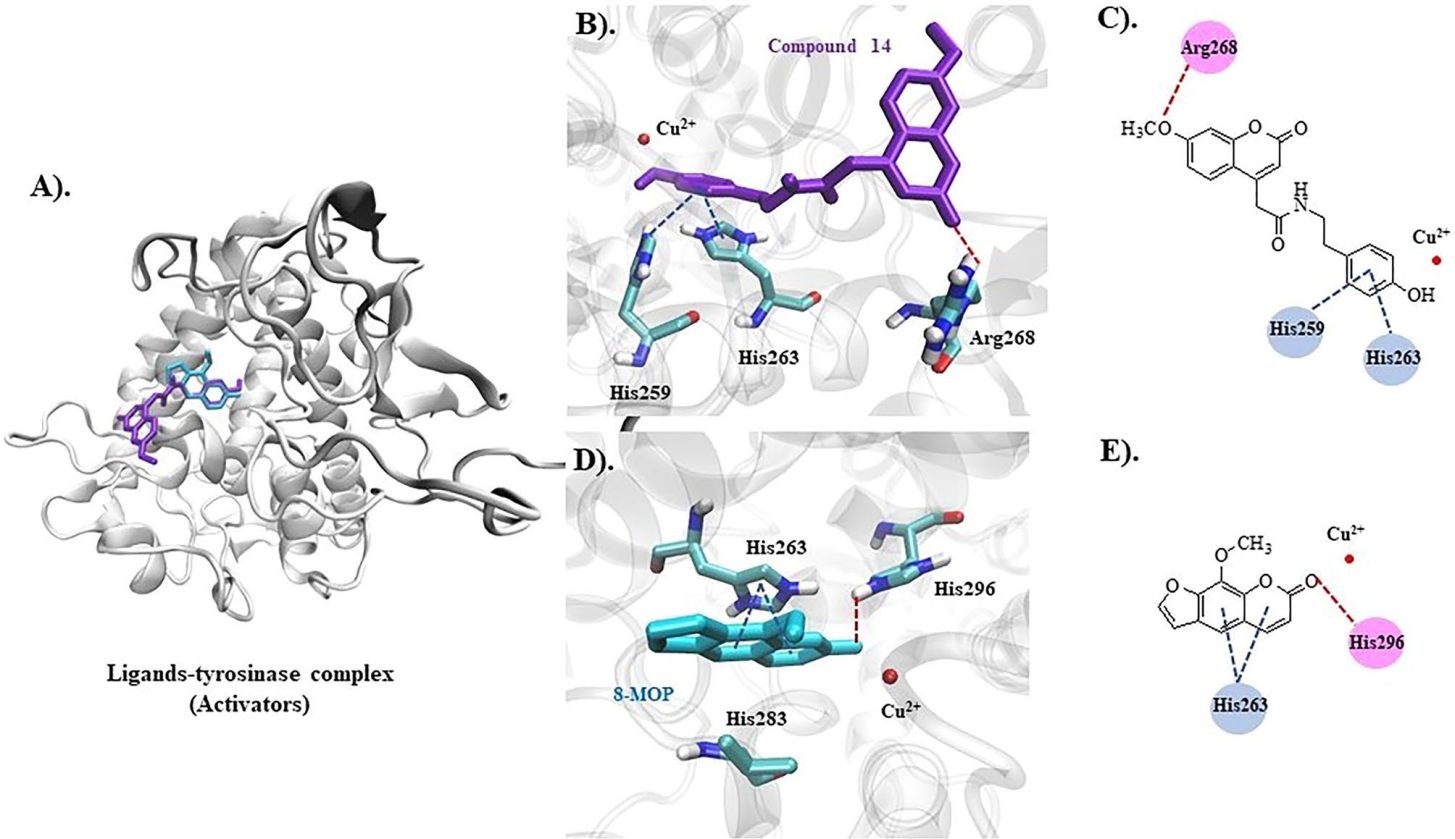
Activators (compounds **14** and 8-MOP) bound with the tyrosinase protein. (**A**) Molecular docking model of compounds **14** (violet) and 8-MOP (blue) on the tyrosinase active site. Ligand interaction diagrams of compounds **14** (**B**,**C**) and 8-MOP (**D**,**E**), involving the hydrogen bond and π–π interaction, as respectively illustrated in red and blue.

For the tyrosinase activator, the compound **14** (ΔG = − 7.98 kcal/mol) had a lower binding energy than 8-MOP (ΔG = − 6.98 kcal/mol) in the case of the activator. The carbonyl oxygen atom on the coumarin ring interacted with Arg268 via a hydrogen bond. Furthermore, the benzyl group on the tyramine moiety interacted with His259 and His263, whereas 8-MOP only interacted with His263 via π-π stacking and His296 via hydrogen bonding (Fig. 6). Likewise, the methoxy substitution at the C7-position in compounds **10** and **14** reduced the docking score, but compound **14** converted the phenolic group to Cu^2+^, whereas compound **10** used the coumarin ring (Fig. 4E). Despite the fact that compound **18** converted the phenolic group to Cu^2+^ similar to the compound **14**, the hydroxyl group, inserted at the C6-position in the coumarin, reduced the docking score, which could be explained by the fact that compound **18** had a lower binding preference for this site than compound **14**.

To summarize, the docking results show that the inhibitor and activator efficacy can be weighed by two factors: binding preference and binding characteristics. The inhibitory efficacy could be attributed to the distance between the coumarin ring and Cu^2+^, whereas the activator efficacy could be attributed to the phenolic group's proximity to Cu^2+^. However, the compound needed to have a strong binding preference for the tyrosinase copper site.

### Cell viability assay

**Figure 7:**
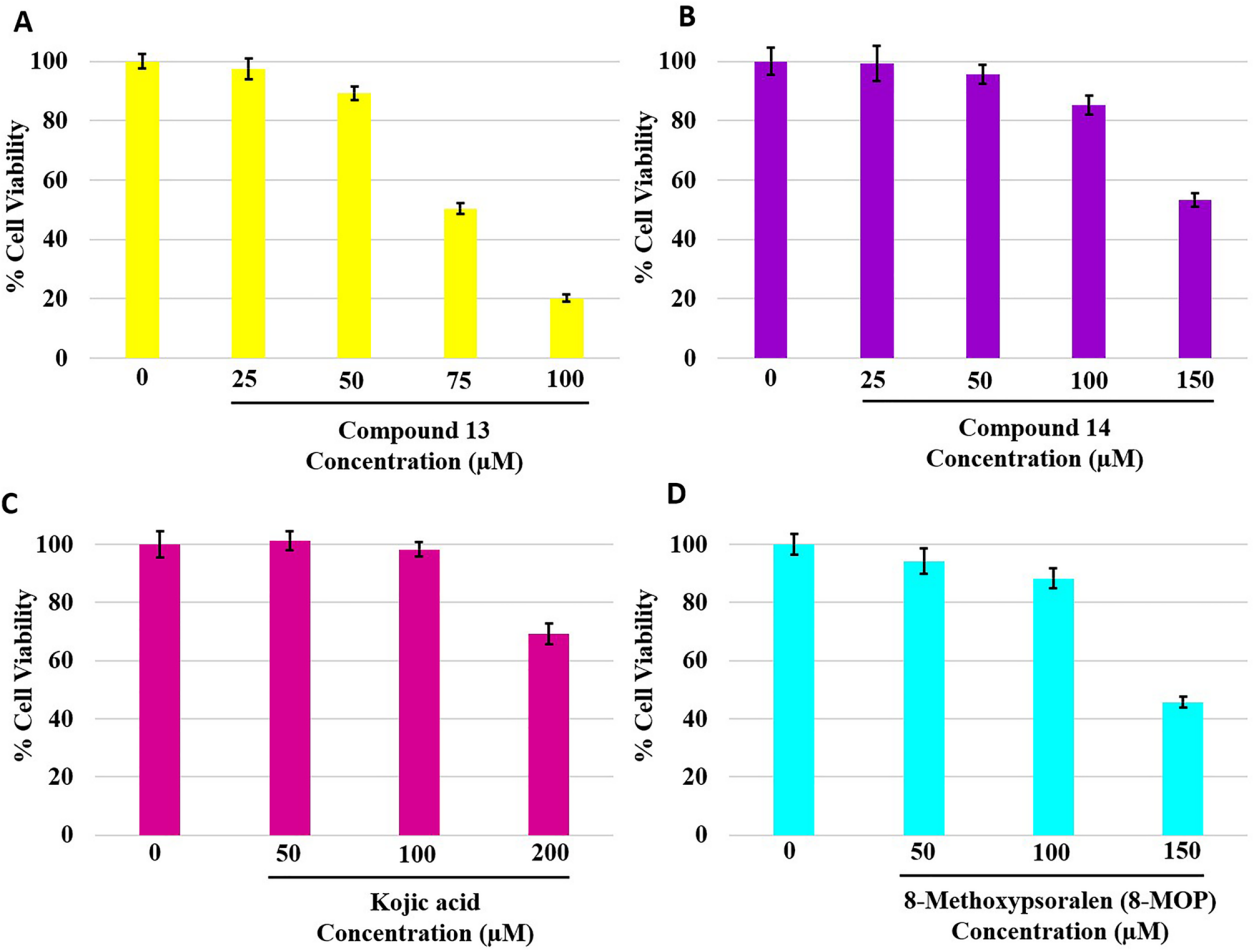
%Cell viability of B16F10 cells after incubation with the synthetic coumarin derivatives for 48 h.

From the synthetic series, we selected the most potent tyrosinase inhibitor (compound **13**) and activator (compound **14**) and evaluated their toxicity to the skin cells by MTT assay in the B16F10 melanoma cells from *Mus musculus* skin. B16F10 cells were incubated with compounds **13** (25–100 µM) and **14** (25–150 µM) for 48 h. KA (50–200 µM) and 8-MOP (50–150 µM) were used as reference compounds for the tyrosinase inhibitor and the activator, respectively. A percentage of cell viability was calculated and illustrated in Fig. 7A–D. Compounds **13** did not show cytotoxicity to B16F10 cells at 25–50 µM (%viability > 80%). Cytotoxicity increased when the concentration of the compounds increased to 75 µM. (%viability decreased to 50%). Compound **13** had a narrow cytotoxicity profile in comparison to KA. Compound **14** was less cytotoxic than compound **13** and showed a cytotoxicity profile similar to 8-MOP.

### Melanin content assay

**Figure 8:**
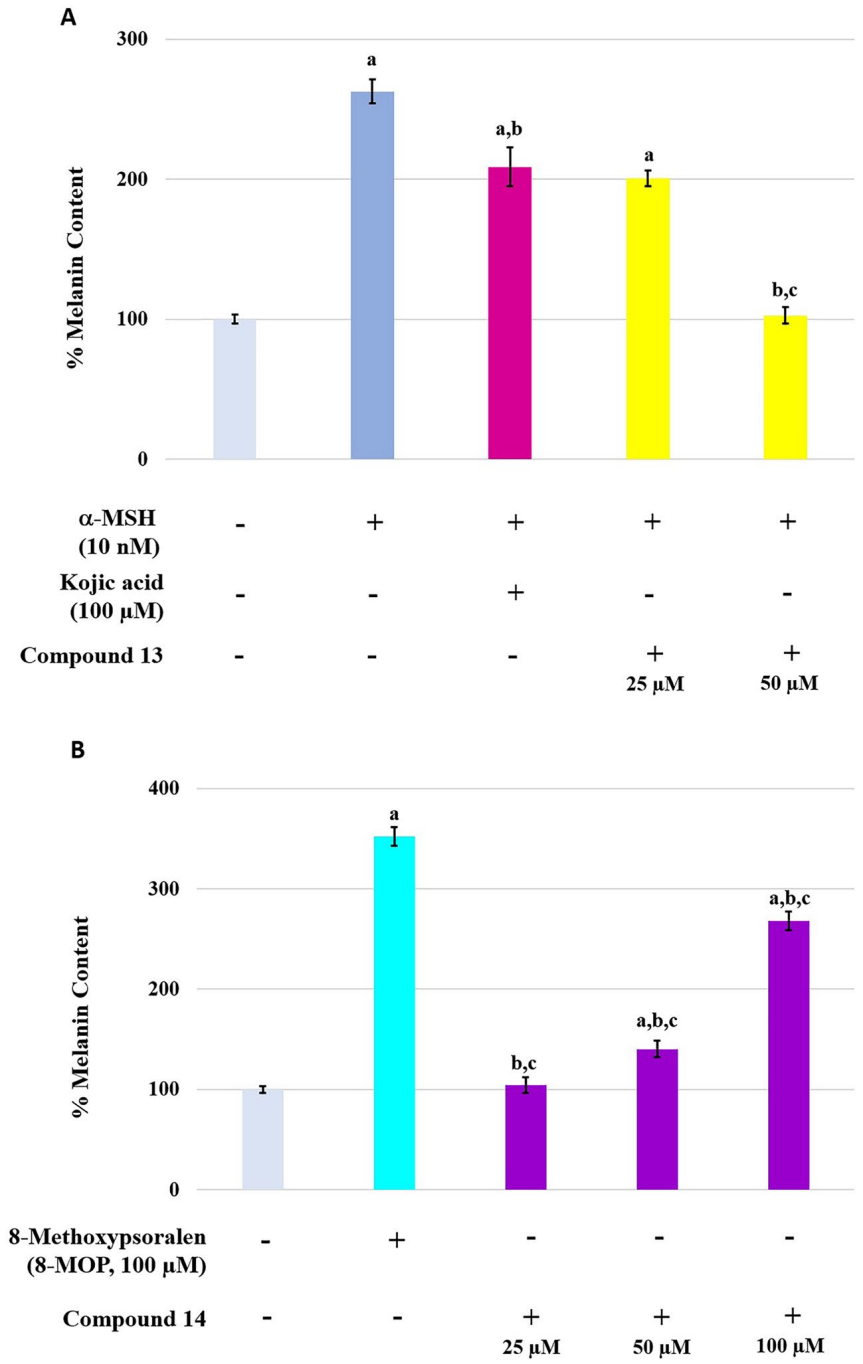
Effect of the tyrosinase inhibitors (**A**) and activators (**B**) on melanin content in B16F10 cells for 48 h. a, b, and c displayed statistically significant differences (P < 0.05) against the untreated group, the α-MSH or 8-MOP-treated group, and the KA or 8-MOP-treated group, respectively.

Compound **13** and compound **14** were also determined for their melanogenesis activities in the B16F10 cells. Intracellular melanin content was measured. In contrast to the result from the tyrosinase inhibition assay, compound **13** at 50 μM suppressed melanogenesis with approximately two times higher potency than KA at 100 μM. When compared to the untreated cells, compound **13** significantly suppressed melanin synthesis at 50 μM whereas 25 μM inhibited melanogenesis equally to KA at 100 μM (Fig. 8A). As an activator, compound **14** showed less stimulation at 25 and 50 μM and effectively enhanced melanogenesis in B16F10 cells in a dose-dependent manner. However, the increase in melanin content in the cells was lower than cells treated with 8-MOP (Fig. 8B).

## Discussion

Twelve coumarin derivatives (compound **7**–**18**) were developed, their anti-oxidative activity and effect on the tyrosinase enzyme were evaluated. The effect of melanogenesis in B16F10 cells was studied on the most potent tyrosinase inhibitor (compound **13**) and activator (compound **14**). Compounds **7**, **9**, **11**–**13**, and **15**–**18** demonstrated excellent anti-lipid peroxidation activity (Table 1). The anti-lipid peroxidation activity tended to depend on R_1_ and R_2_ substituents on the coumarin moiety of the designed compounds. In general, the 2-(7-methoxy-2-oxo-2*H*-chromen-4-yl)acetamides (compounds **11**–**14**, R_1_ = OCH_3_ and R_2_ = H) exhibited lower activity than other series bearing a phenolic group on the coumarin ring (compounds **7**–**10** and **15**–**18**). In each series, the compounds derived from phenylalanine ethyl ester (compounds **7**, **11**, and **15**) showed higher potency than the tyrosine (compounds **8**, **12**, and **16**), tryptophan (compounds **9**, **13**, and **17**), and tyramine (compounds **10**, **14**, and **18**) derivatives. The tyramine derivatives showed the weakest anti-lipid peroxidation activity than other amino acid ester derived-compounds in each series. This might be due to lower lipophilicity of tyramine comparing to the amino acid esters. The anti-lipid peroxidation results suggested additional benefits of the coumarin derivatives for cosmeceutical applications.

In the tyrosinase inhibition activity evaluation, the synthesized compounds showed different impacts on the enzyme (Table 1). The phenylalanine ethyl ester (compounds **7**, **11**, **15**) and tryptophan ethyl ester derivatives (compounds **9**, **13**, **17**) inhibited the function of tyrosinase, while, surprisingly, the tyrosine ethyl ester (compounds **8**, **12**, **16**) and tyramine derivatives (compounds **10**, **14**, **18**) tended to activate the enzyme. The most potent tyrosinase activator and inhibitor were compounds **13** and **14**, respectively.

An enzyme activator is a molecule that binds to an enzyme and thus increases its activity. Enzyme activators often bind at the allosteric site and modify the rate of enzyme function. In some cases, when a substrate binds to one catalytic subunit of an enzyme, this can trigger an increase in substrate affinity as well as catalytic activity in the enzyme's other subunits. Many chalcones, phenolic compounds^20,23,24^, flavonoids^25^, and coumarins^26,27^ were reported as tyrosinase activators, but the mechanism of the activity is still unknown. Similar to our findings, previous studies have shown that substituent changes can cause a switch from an activator to an inhibitor^20,28^. For example, You, A. et al.^28^ showed that adding a thiosemicarbazide group to amino-acetophenones made them inhibitors instead of activators. However, the underlying mechanism was still unknown.

An enzyme kinetic study revealed that compound **13**, the most potent inhibitor, uncompetitively inhibited the tyrosinase enzyme. In uncompetitive inhibition, the inhibitor binds at a different site from the substrate and combines with the enzyme–substrate complex (ES), but not with the free enzyme (E). From molecular docking, different binding poses between the inhibitors and the activators at the active site of the mushroom tyrosinase enzyme were observed (Fig. 4). These findings might explain their different effects on the tyrosinase enzyme. Compounds **13** and **14** had lower binding energies than KA and 8-MOP, respectively (Fig. 3). The enzyme inhibition assay suggested that compound **13** (IC_50_ 68.86 µM) was less potent than KA (IC_50_ 4.75 µM). Compound **14** (%activity 123.41) was determined to be a stronger tyrosinase activator than the reference compound 8-MOP (%activity 101.05). Compounds **13** exhibited a minimally different profile in cytotoxicity against B16F10 melanoma cells in comparison to compound **14**. In the melanin content assay, compound **13** exhibited higher melanin suppression at a lower concentration (50 μM) than KA (100 μM) (Fig. 8A); however, this contrasted with the results obtained from the enzyme inhibition assay. SwissADME prediction (accessed from http://www.swissadme.ch)^29^ showed that compound **13** (cLogP 3.27) exhibited higher lipophilicity than KA (cLogP − 0.16). This suggested that compound **13** is a lipophilic molecule, allowing its ease of penetrating through the cell membrane, thus demonstrating approximately two folds higher melanin suppression. KA, currently used as an inhibitor for melanin inhibition, also showed activity against murine tyrosinase in a broad range (> 100 μM)^30^. Mushroom tyrosinase was used in the enzyme inhibition assay; however, B16F10 cells from murine were used in the melanin content assay. Certain amino acid sequence differences in the primary structure of tyrosinase from different species contribute to variances in binding affinity, resulting in irrelevant results^31^. In addition, mechanisms involved in melanosome activity by other proteins involved in melanogenesis may explain the differences between results from melanoma cells and mushroom tyrosinase^32^. Compound **14** stimulated melanin production in B16F10 cells in the same manner as the mushroom enzyme inhibition assay.

## Conclusion

The effects of 12 synthetic coumarin derivatives on tyrosinase activity and melanogenesis have been studied. Compound **13** was discovered to possess strong anti-lipid peroxidation activity and a moderately potent, uncompetitive tyrosinase inhibitor. Compound **14** demonstrated potent enzyme-activating potency. Molecular docking showed that the tyrosinase activators and inhibitors bound to different areas near the catalytic site. Compound **13** suppressed melanogenesis with a higher potency than KA in B16F10 cells. Compound **14** stimulated melanin production in a dose-dependent manner. Compounds **13** and **14** are potential candidates for the treatment of skin pigmentation disorders and as cosmeceuticals.

## Methods

All chemicals were purchased from Sigma-Aldrich or Merck AG. The progress of the reactions and the purities of the compounds were checked by thin layer chromatography (TLC) on silica gel 60 F_254_ aluminum sheets (Merck AG).

### Chemistry

Twelve coumarin derivatives were prepared in 2 steps as shown in Fig. 1. The synthesis process started with Pechmann condensation between the corresponding resorcinol derivatives and 1,3-acetonedicarboxylic acid to afford 2-(2-oxo-2*H*-chromen-4-yl)acetic acids (**4–6**). The intermediates were consecutively coupled with the corresponding amino acid esters or tyramine to obtain the desired product (**7–18**) in good yields^18,33^.

#### General method for preparation of 2-(2-oxo-2H-chromen-4-yl)acetic acids (***4***–***6***)

At 0 °C, resorcinol derivatives (27.25 mmol) were dissolved in 70% sulfuric acid (20 mL), and then 1,3-acetonedicarboxylic acid (27.25 mmol) was added. The reaction mixture was then stirred for 24 h at room temperature. Iced water was poured into the reaction mixture to form precipitates. The solid was collected by vacuum filtration and washed with cold water. The desired product was purified by silica gel column chromatography using a mixture of dichloromethane:methanol (97:3) as the mobile phase.

#### General method for preparation of 2-(2-oxo-2H-chromen-4-yl)acetamide derivatives (***7***–***18***)

*O*-(7-azabenzotriazole-1-yl)-*N*,*N*,*N*',*N*'-tetramethyluronium (HATU, 1.04 g, 2.73 mmol) was added to the solution of 2-(2-oxo-2*H*-chromen-4-yl)acetic acids (4–6, 2.27 mmol) in DMF (3 mL) and stirred at room temperature for 10 min. The corresponding amines (2.73 mmol) and *N*, *N*-diisopropylethylamine (DIEA, 3.41 mmol) were added, and the mixture was further stirred for 24 h. The mixture was partitioned into 50 mL of water (50 mL) and ethyl acetate (3 × 20 mL). The combined organic layer was washed with water (2 × 50 mL) and brine (50 mL). A trace of water in the organic layer was removed by the addition of anhydrous sodium sulfate. The solid was filtered off, and the filtrate was evaporated. The desired product was purified by column chromatography using a mixture of dichloromethane:methanol (99:1) as a mobile phase.

Melting points of the synthesized compounds were recorded using the Mel-TEMP II Laboratory Devices. The structures of the synthesized compounds were confirmed by infrared spectroscopy, (Spectrum One, Perkin Elmer, Massachusetts, USA), ^1^H- (500 MHz) and ^13^C- (125 MHz) nuclear magnetic resonance spectroscopy (Avance NEO, Bruker, Massachusetts, USA), High-resolution mass spectrometry (MAT 95XL, Thermo Finnigan, California, USA). The purity of the synthesized compounds was determined using high-performance column chromatography (HPLC).

#### 2-(7-hydroxy-2-oxo-2H-chromen-4-yl)acetic acid (***4***)

Brown solid (55% yield, recryst. from methanol), m.p. 210–212 °C; IR (cm^−1^, KBr); 3496, 3126, 2939, 1733, 1706, 1246; ^1^H-NMR (ppm, DMSO-d_6_): δ 12.72 (1H, s), 10.55 (1H, s), 7.52 (1H, d, *J* = 8.7 Hz), 6.79 (1H, dd, *J* = 8.7, 2.4 Hz), 6.72 (1H, d, *J* = 2.4 Hz), 3.81 (2H, s); ^13^C-NMR: (ppm, DMSO-d_*6*_): 161.48, 160.42, 155.01, 153.64, 126.69, 124.35, 113.06, 112.07, 110.30, 102.33, 18.22; HR-MS: calcd. for C_11_H_8_O_5_ (M + 1)^+^: 221.0450, found: 221.0457. Purity = 100.00%.

#### 2-(7-methoxy-2-oxo-2H-chromen-4-yl)acetic acid (***5***)

Pale pink solid (85% yield, recryst. from methanol), m.p. 180–181 °C; IR (cm^−1^, KBr); 3208, 3025, 2956, 1720, 1681, 1604, 1088, 757, 730; ^1^H-NMR (ppm, DMSO-d_6_): δ 12.73 (1H, s), 7.60 (1H, d, *J* = 8.8 Hz), 6.97 (1H, d, *J* = 2.5 Hz), 6.92–6.94 (1H, dd, *J* = 8.8, 2.5 Hz), 6.27 (1H, s), 3.82 (2H, s), 3.84 (3H, s); ^13^C-NMR: (ppm, DMSO-d_6_) 162.52, 160.25, 154.92, 153.52, 126.56, 124.40, 113.25, 112.21, 111.27, 100.86, 56.04, 18.30; HR-MS: calcd. for C_12_H_10_O_5_ (M + 1)^+^: 235.0607, found: 235.0616. Purity = 100.00%.

#### 2-(6-hydroxy-7-methoxy-2-oxo-2H-chromen-4-yl)acetic acid (***6***)

White solid (71% yield, recryst. from methanol), m.p. 163–165 °C; IR (cm^−1^, KBr); 3185, 3019, 2969, 1727, 1662, 1617, 1002, 793, 743; ^1^H-NMR (ppm, DMSO-d_*6*_): δ 12.76 (1H, s), 9.36 (1H, s), 7.02 (1H, s), 6.98 (1H, s), 6.26 (1H, s), 3.83 (3H, s), 3.77 (2H, s); ^13^C-NMR (500 MHz, ppm, DMSO-d_*6*_): 170.73, 160.59, 151.87, 149.95, 148.07, 143.65, 113.22, 111.86, 109.30, 100.34, 56.32, 37.69; HR-MS: calcd. for C_12_H_10_O_6_ (M + 1)^+^: 251.0556, found: 251.0565. Purity = 98.76%.

#### Ethyl 2-(2-(7-hydroxy-2-oxo-2H-chromen-4-yl)acetamido)-3-phenylpropanoate (***7***)

White solid (58% yield, recryst. from methanol), m.p. 139–140 °C; IR (cm^−1^, KBr); 3284, 1709, 1656, 1609, 1564, 1394, 1268, 1214, 745, 701; ^1^H-NMR (ppm, DMSO-d_6_): δ10.52 (1H, s), 8.73, 8.71 (1H, d, *J* = 7.85 Hz), 7.45, 7.43 (1H, d, *J* = 8.51 Hz), 7.19–7.27 (5H, m), 6.69–6.71 (2H, m), 6.11 (1H, s), 4.45–4.49 (1H, m), 4.05 (2H, q, *J* = 7.0 Hz), 3.63 (1H, d, *J* = 3.5 Hz), 2.88–2.93 (1H, dd, *J* = 13.8, 9.6 Hz), 3.03–3.07 (1H, dd, *J* = 13.8, 5.3 Hz), 1.10 (1H, t, *J* = 7.0 Hz); ^13^C-NMR: (ppm, DMSO-d_6_): 171.45, 167.97, 161.25, 160.31, 155.10, 151.02, 137.17, 129.22, 128.36, 126.79, 126.73, 112.96, 111.84, 111.50, 102.39, 60.82, 53.88, 38.52, 36.77, 14.08; HR-MS: calcd. for C_22_H_21_NO_6_ (M + 1)^+^: 396.1447, found 396.1437. Purity = 97.96%.

#### Ethyl 2-(2-(7-hydroxy-2-oxo-2H-chromen-4-yl)acetamido)-3-(4-hydroxyphenyl)propanoate (***8***)

White solid (90% yield, recryst. from methanol), m.p. 152–154 °C; IR (cm^−1^, KBr); 3403, 3295, 1715, 1694, 1661, 1555, 1370, 1269, 1216, 703; ^1^H-NMR (ppm, DMSO-d_6_): δ 10.69 (1H, s), 9.23 (1H, s), 8.65 (1H, d, J = 7.8 Hz), 7.45 (1H, d, *J* = 8.7 Hz), 6.95–7.00 (2H, m), 6.67–6.74 (2H, m), 6.61–6.66 (2H, m), 6.13 (1H, s), 4.36 (1H, ddd, *J* = 9.2, 7.8, 5.5 Hz), 3.98–4.07 (2H, m), 3.59–3.68 (2H, m), 2.90 (1H, dd, *J* = 13.9, 5.5 Hz), 2.78 (1H, dd, *J* = 13.9, 9.2 Hz), 1.09 (3H, t, *J* = 7.1 Hz); ^13^C-NMR: (ppm, DMSO-d_6_): 171.85, 168.22, 161.59, 160.62, 156.48, 155.38, 151.40, 130.44, 127.42, 127.12, 115.47, 113.29, 112.11, 111.79, 102.67, 61.01, 54.55, 38.77, 36.37, 14.37; HR-MS: calcd. for C_22_H_21_NO_7_ (M + 1)^+^: 412.1396, found: 412.1391. Purity = 97.64%.

#### Ethyl 2-(2-(7-hydroxy-2-oxo-2H-chromen-4-yl)acetamido)-3-(1H-indol-2-yl)propanoate (***9***)

White solid (42% yield, recryst. from methanol), m.p. 185–188 °C; IR (cm^−1^, KBr); 3414, 3305, 1721, 1704, 1652, 1564, 1397, 1214, 1139, 744; ^1^H-NMR (ppm, DMSO-d_*6*_): δ 10.86 (1H, s), 10.50 (1H, s), 8.71 (1H, d, *J* = 7.6 Hz), 7.49 (1H, d, *J* = 7.9 Hz), 7.42–7.47 (1H, m), 7.34 (1H, d, *J* = 8.1 Hz), 7.15 (1H, d, *J* = 2.3 Hz), 7.03–7.10 (1H, m), 6.93–7.01 (2H, m), 6.70 (2H, dd, *J* = 6.7), 6.13 (1H, s), 4.51 (1H, dt, *J* = 7.6, 5.7 Hz), 4.02 (2H, q, *J* = 7.1 Hz), 3.06 (1H, dd, *J* = 14.6, 2.6 Hz), 3.17 (1H, dd, *J* = 14.6, 5.6 Hz), 3.66 (2H, m), 1.06 (3H, t, *J* = 7.1 Hz); ^13^C-NMR: (ppm, DMSO-d_*6*_): 171.81, 168.00, 161.26, 160.51, 155.08, 151.18, 127.22, 126.77, 123.85, 121.14, 118.57, 118.13, 112.98, 111.77, 111.54, 111.57, 109.5, 102.37, 60.68, 55.54, 38.37, 27.20, 14.01; HR-MS: calcd. for C_24_H_22_N_2_O_6_ (M + 1)^+^: 435.1556, found: 435.1583. Purity = 96.50%.

#### N-(4-hydroxyphenethyl)-2-(7-hydroxy-2-oxo-2H-chromen-4-yl)acetamide (***10***)

White solid (23% yield, recryst. from methanol), m.p. 223–225 °C; IR (cm^−1^, KBr); 3316, 3194, 3146, 1698, 1621, 1563, 1326, 1241, 1141, 826; ^1^H-NMR (ppm, DMSO-d_*6*_): δ 10.52 (1H, s), 9.14 (1H, s), 8.18 (1H, t, *J* = 5.6 Hz), 7.55 (1H, d, *J* = 8.8 Hz), 6.89–6.97 (2H, m), 6.69–6.81 (2H, m), 6.61–6.67 (2H, m), 6.13 (1H, s), 3.60 (2H, s), 3.22 (2H, dt, *J* = 7.1, 56 Hz), 2.57 (2H, t, *J* = 7.2 Hz); ^13^C-NMR: (ppm, DMSO-d_*6*_): 167.85, 161.32, 160.36, 155.79, 155.15, 151.32, 129.61, 129.46, 126.88, 115.21, 113.01, 111.87, 111.61, 102.42, 40.92, 39.04, 34.29; HR-MS: calcd. for C_19_H_17_NO_5_ (M + 1)^+^: 340.1185, found: 340.1195. Purity = 100.00%.

#### Ethyl 2-(2-(7-methoxy-2-oxo-2H-chromen-4-yl)acetamido)-3-phenylpropanoate (***11***)

White solid (55% yield, recryst. from methanol), m.p. 144–145 °C; IR (cm^−1^, KBr); 3307, 3029, 2935, 1728, 1645, 1613, 1540, 1288, 1266, 744; ^1^H-NMR (ppm, DMSO-d_*6*_): δ 8.74, 8.72 (1H, d, *J* = 7.8 Hz), 7.52, 7.54 (1H, d, *J* = 8.8 Hz), 7.18–7.27 (5H, m), 6.96–6.97 (1H, d, *J* = 2.5 Hz), 6.84–6.86 (1H, dd, *J* = 2.5, 8.8 Hz), 6.19 (1H, s), 4.45–4.50 (1H, m), 4.03–4.07 (2H, q, *J* = 7.1 Hz), 3.85 (3H, s), 3.67 (2H, s), 2.88–2.93 (1H, dd, *J* = 13.8, 9.6 Hz), 3.03–3.07 (1H, dd, *J* = 13.8, 5.3 Hz), 1.09–1.11 (3H, t, *J* = 7.1 Hz); ^13^C-NMR: (ppm, DMSO-d_*6*_) 171.43, 167.88, 162.46, 160.14, 155.03, 150.87, 137.15, 129.20, 128.35, 126.71, 126.60, 112.73, 112.56, 112.16, 100.94, 60.82, 56.09, 53.86, 38.51, 36.76, 14.07; HR-MS: calcd. for C_23_H_23_NO_6_ (M + 1)^+^: 410.1604, found: 410.1593. Purity = 99.40%.

#### Ethyl 2-(2-(7-methoxy-2-oxo-2H-chromen-4-yl)acetamido)-3-(4-hydroxyphenyl) propanoate (***12***)

White solid (70% yield, recryst. from methanol), m.p. 154–155 °C; IR (cm^−1^, KBr); 3311, 3338, 3070, 2940, 1734, 1671, 1649, 1612, 1517, 1294, 1216, 862, 833; ^1^H-NMR (ppm, DMSO-d_*6*_): δ 9.23 (1H, s), 8.67, 8.68 (1H, d, *J* = 7.8 Hz), 7.46, 7.47 (1H, d, *J* = 8.8 Hz), 6.99, 7.00 (2H, d, *J* = 8.4 Hz), 6.96 (1H, d, *J* = 2.5 Hz), 6.84–6.87 (2H, dd, *J* = 8.8, 2.5 Hz), 6.64–6.66 (2H, d, *J* = 8.4 Hz), 6.22 (1H, s), 4.36–4.41 (1H, m), 4.04–4.07 (2H, q, *J* = 7.1 Hz), 3.85 (3H, s), 3.68 (2H, s), 2.91–2.95 (1H, dd, *J* = 13.8, 5.3 Hz), 2.76–2.81 (1H, dd, *J* = 13.8, 9.5 Hz), 1.11 (3H, t, *J* = 7.1 Hz); ^13^C-NMR: (ppm, DMSO-d_*6*_); 171.67, 167.84, 162.44, 160.15, 156.18, 155.00, 150.93, 130.16, 127.13, 126.56, 115.16, 112.84, 112.59, 112.08, 100.99, 60.73, 56.06, 54.19, 38.51, 36.08, 14.09; HR-MS: calcd. for C_23_H_23_NO_7_ (M + 1)^+^: 426.1553, found: 426.1539. Purity = 98.64%.

#### Ethyl 3-(1H-indol-2-yl)-2-(2-(7-methoxy-2-oxo-2H-chromen-4-yl) acetamido) propanoate (***13***)

White solid (80% yield, recryst. from methanol), m.p. 162–163 °C; IR (cm^−1^, KBr); 3421, 3311, 3076, 2929, 1725, 1659, 1641, 1616, 738, 710; ^1^H-NMR (ppm, DMSO-d_*6*_): δ 10.81 (1H, s), 8.72, 8.74 (1H, d, *J* = 7.6 Hz), 7.47–7.51 (2H, dd, *J* = 7.1, 8.8 Hz), 7.34, 7.35 (1H, d, *J* = 8.0 Hz), 7.16 (1H, d, *J* = 2.5 Hz), 6.99–7.08 (3H, m), 6.79–6.82 (1H, dd, *J* = 8.8, 2.5 Hz), 6.21 (1H, s), 4.49–4.54 (1H, m), 4.05–4.09 (2H, q, *J* = 7.1 Hz), 3.84 (3H, s), 3.69, 3.70 (2H, d, *J* = 5.5 Hz), 3.16–3.20 (1H, dd, *J* = 14.6, 5.5 Hz), 3.04–3.09 (1H, dd, *J* = 14.6, 8.8 Hz); ^13^C-NMR: (ppm, DMSO-d_*6*_): 171.80, 167.93, 162.44, 160.15, 154.99, 151.00, 139.20 127.20, 126.51, 123.87, 121.14, 118.57, 118.13, 112.76, 112.61, 112.09, 111.57, 109.49, 100.87, 60.71, 56.08, 53.51, 38.50, 27.19, 14.02; HR-MS: calcd. for C_25_H_24_N_2_O_6_ (M + 1)^+^: 449.1713, found: 449.1704. Purity = 95.73%.

#### N-(4-hydroxyphenethyl)-2-(7-methoxy-2-oxo-2H-chromen-4-yl)acetamide (***14***)

White solid (83% yield, recryst. from methanol), m.p. 152–153 °C; IR (cm^−1^, KBr); 3293 (O–H stretching), 3079, 2931, 1697, 1641, 1614, 881, 826; ^1^H-NMR (ppm, DMSO-d_*6*_): δ 9.14 (1H, s), 8.19 (1H, t, *J* = 5.6 Hz), 7.60 (1H, d, *J* = 8.87 Hz), 6.91–6.98 (3H, m), 6.63–6.66 (2H, m), 6.22 (1H, s), 3.85 (3H, s), 3.63 (1H, s), 3.21–3.25 (2H, q, *J* = 7.1 Hz), 2.58 (2H, d, *J* = 7.2 Hz); ^13^C-NMR: (ppm, DMSO-d_*6*_) 167.81, 162.79, 160.52, 156.10, 155.39, 151.48, 129.93, 129.75, 127.00, 115.51, 113.17, 113.04, 112.46, 101.33, 56.39, 41.18, 39.34, 34.57; HR-MS: calcd. for C_20_H_19_NO_5_ (M + 1)^+^: 354.1341, found: 354.1325. Purity = 99.94%.

#### Ethyl 2-(2-(6-hydroxy-7-methoxy-2-oxo-2H-chromen-4-yl)acetamido)-3-phenylpropanoate (***15***)

White solid (57% yield, recryst. from methanol), m.p. 184–185 °C; IR (cm^−1^, KBr); 3325, 3033, 2938, 1723, 1676, 1649, 1567, 1289, 1213, 859, 832; ^1^H-NMR (ppm, DMSO-d_*6*_): δ 9.24 (1H, s), 8.66, 8.68 (1H, d, *J* = 7.7 Hz), 7.17–7.25 (5H, m), 7.08 (1H, s), 7.01 (1H, s), 6.16 (1H, s), 4.43–4.47 (1H, m), 4.01–4.06 (2H, q, *J* = 7.0 Hz), 3.87 (3H, s), 3.60 (2H, s), 2.90–3.04 (2H, dd, *J* = 13.8, 7.4 Hz), 1.08 (3H, t, *J* = 7.1 Hz); ^13^C-NMR: (ppm, DMSO-d_*6*_); 171.65, 168.17, 160.85, 152.10, 150.97, 148.30, 143.81, 137.41, 129.50, 128.64, 127.01, 112.89, 112.22, 110.15, 100.44, 61.08, 56.65, 54.32, 38.85, 37.12, 14.33; HR-MS: calcd. for C_23_H_23_NO_7_ (M + 1)^+^: 426.1553, found: 426.1537. Purity = 98.79%.

#### Ethyl 2-(2-(6-hydroxy-7-methoxy-2-oxo-2H-chromen-4-yl)acetamido)-3-(4-hydroxyphenyl) propanoate (***16***)

White solid (65% yield, recryst. from methanol), m.p. 198–200 °C; IR (cm^−1^, KBr); 3312, 3373, 3025, 2929, 1706, 1658, 1614, 1516, 1217, 851, 832; ^1^H-NMR (ppm, DMSO-d_*6*_): δ 9.21, 9.17 (2H, s), 8.60, 8.61 (1H, d, *J* = 7.6 Hz), 7.09 (1H, s), 7.01 (1H, s), 6.95–6.96 (2H, d, *J* = 8.5 Hz), 6.63–6.61 (2H, d, *J* = 8.5 Hz), 6.19 (1H, s), 4.33–4.37 (1H, m), 4.00–4.04 (2H, q, *J* = 7.0 Hz), 3.87 (3H, s), 3.61 (2H, 2 s), 2.79–2.91 (2H, dd, *J* = 14.6, 8.8 Hz), 1.08 (3H, t, *J* = 7.1 Hz); ^13^C-NMR: (ppm, DMSO-d_*6*_); 171.44, 167.81, 160.52, 156.17, 151.78, 150.75, 147.99, 143.50, 130.12, 127.06, 115.17, 112.56, 111.93, 109.85, 100.15, 60.66, 56.33, 54.39, 38.51, 36.14, 14.04; HR-MS: calcd. for C_23_H_23_NO_8_ (M + 1)^+^: 442.1502, found: 442.1496. Purity = 100.00%.

#### Ethyl 3-(1H-indol-2-yl)-2-(2-(6-hydroxy-7-methoxy-2-oxo-2H-chromen-4-yl) acetamido) propanoate (***17***)

White solid (53% yield, recryst. from methanol), m.p. 173–174 °C; IR (cm^−1^, KBr); 3313, 3058, 2977, 1708, 1664, 1625, 1561, 1289, 863, 834; ^1^H-NMR (ppm, DMSO-d_*6*_): δ 10.83 (1H, s), 9.24 (1H, s), 8.68, 8.69 (1H, d, *J* = 7.4 Hz), 7.47, 7.48 (1H, d, *J* = 7.8 Hz), 7.32, 7.33 (1H, d, *J* = 8.3 Hz), 7.14 (1H, s), 6.95–7.07 (4H, m), 6.19 (1H, s), 4.38–4.52 (1H, m), 3.97–4.02 (2H, q, *J* = 7.1 Hz), 3.87 (3H, s), 3.59–3.68 (2H, q, *J* = 14.6 Hz), 3.05–3.18 (1H, dd, *J* = 14.6, 7.8 Hz), 1.02–1.04 (1H, t, *J* = 7.1 Hz); ^13^C-NMR: (ppm, DMSO-d_*6*_): 172.02, 168.24, 160.86, 152.11, 151.12, 148.30, 143.82, 136.57, 127.53, 124.15, 121.44, 118.86, 118.42, 112.80, 112.29, 111.89, 110.12, 109.72, 100.48, 60.95, 56.65, 53.98, 38.79, 27.55, 14.26; HR-MS: calcd. for C_25_H_24_N_2_O_7_ (M + 1)^+^: 465.1662, found: 465.1648. Purity = 97.42%.

#### N-(4-hydroxyphenethyl)-2-(6-hydroxy-7-methoxy-2-oxo-2H-chromen-4-yl)acetamide (***18***)

White solid (48% yield, recryst. from methanol), m.p. 227–228 °C; IR (cm^−1^, KBr); 3314, 3270, 3079, 2924, 1707, 1661, 1626, 738, 710; ^1^H-NMR (ppm, DMSO-d_*6*_): δ 9.30 (1H, s), 9.13 (1H, s), 8.16 (1H, t, *J* = 5.6 Hz), 7. 90 (1H, s), 7.01 (1H, s), 6.92–6.95 (2H, m), 6.62–6.65 (2H, m), 6.19 (1H, s), 3.87 (3H, s), 3.57 (2H, s), 3. 91–3.23 (2H, q, *J* = 6.8 Hz), 2.56–2.59 (2H, t, *J* = 7.3 Hz); ^13^C-NMR: (ppm, DMSO-d_*6*_) 167.61, 160.63, 155.79, 151.83, 150.98, 148.07, 143.54, 129.62, 129.50, 115.27, 112.90, 112.04, 109.76, 100.24, 56.35, 41.08, 39.19, 34.40; HR-MS: calcd. for C_20_H_19_NO_6_ (M + 1)^+^: 370.1291, found: 370.1275. Purity = 100.00%.

### Lipid peroxidation inhibitory activity

This method was modified from our previous work^18^. Briefly, the test compound (2.0 mg) was dissolved in 0.5 mL of 2.5% linoleic acid in ethanol, 8.0 mL of 10 mM phosphate buffer (pH 7.0), and 2.0 mL of purified water in a screw-capped vial. The control was prepared in the same way but without the test sample. Butylated hydroxytoluene (BHT) was used as a positive control. The experiment was performed in duplicate. Each vial was incubated at 45 °C in the dark for 7 days. The reaction mixture (2 mL) was mixed with 20% trichloroacetic acid (1.0 mL) and 1% thiobarbituric acid in 0.05 N sodium hydroxide (1.0 mL) and heated in a water bath for 20 min. The mixture was left to cool down to room temperature, centrifuged at 4000 rpm for 20 min, and the supernatant was drawn to measure absorbance at 532 nm. Percent inhibition was calculated as follows: %Inhibition = [(A_control_-A_sample_)/A_control_] × 100, when A_control_ is the absorbance of TBARS solution without sample, A_sample_ is the absorbance of TBARS solution with sample solution.

### ***Mushroom tyrosinase activity assay***^20^

Samples were dissolved in a mixture of DMSO and the phosphate buffer solution (40%DMSO, 20 µL) and were added into a 96-well microplate filled with phosphate buffer (20 mM, pH 6.8, 140 µL), mushroom tyrosinase (30 U/mL, 20 µL). After 10 min of pre-incubation at room temperature (25 °C), 20 µL of a 0.85 mM L-DOPA (3,4-dihydroxyphenylalanine) solution in phosphate buffer was added to the mixture. (Final concentration of the tested compound equaled 100 µM and final DMSO content equaled 4%). After 20 min of additional incubation, the absorbance at 475 nm was measured using a microplate reader (Power Wave X, California, USA). KA and 8-MOP were used as reference compounds for tyrosinase inhibitors and activators, respectively. Instead of an inhibitor solution, phosphate buffer was added as the negative control. The percentage of tyrosinase activity was calculated as follows: %Activity = (Sample) × 100/Blank, where Blank = (OD of Blank solution at 10 min)—(OD of Blank solution at 0 min) and Sample = (OD of Sample solution at 10 min)—(OD of Sample solution at 0 min). Each sample was analyzed in triplicate (n = 3). The 50 percent inhibition of tyrosinase activity (IC_50_) studies were carried out using six concentrations of compound **13** (ranging from 0.390625 to 80 µM) and KA (ranging from 0.00305 to 400 µM), with the data analyzed using Sigmoidal dose–response model built in the GraphPad Prism 8.0.2 software. Each sample was examined in triplicate.

### Enzyme kinetic study

Utilizing the same protocol as the tyrosinase activity assay, an enzyme kinetics study was conducted with compound **13**. A Michaelis–Menten plot was generated by varying the substrate concentration (25–10,000 µM) and reaction rate under conditions with and without inhibitors. A Lineweaver–Burk plot was generated, and the inhibition mode of the inhibitor was determined based on the variation of K_m_ and V_max_ as the inhibitor concentration changed.

### Molecular docking

The structure of coumarin was obtained from the PubChem database (CID: 323). The PCCDB project (www.pccdb.org) created the reference compounds, and Molview software (www.molview.org) created the other ligands. The Online SMILES Translator and Structure File Generator (http://cactus.nci.nih.gov/translate/) was used to convert a ligand structure into a Protein Data Bank (PDB) format file. The Protein Data Bank (PDBID: 2Y9X; resolution: 2.78) was used to acquire the crystal structure of mushroom tyrosinase (*Agaricus bisporus*). The co-crystallized ligand (tropolone) and water molecules were removed. AutoDock Tools (ADT) version 1.5.6 was used to add the hydrogen atoms.

AutoDock4 was used to evaluate the molecular docking studies between synthesis compounds (**7**–**18**) and mushroom tyrosinase. The grid points in x–y–z were set to 90–90–90 with a spacing of 0.375 Å and the center coordinate (− 11.495, − 23.224, − 40.241). Other parameters in the program were set to default values. As in previous studies^34^, the sampling was carried out using a Lamarckian genetic algorithm (GA) with 50 iterations and a population size of 200. BIOVIA Discovery Studio version 2021 and Visual Molecular Dynamics (VMD) package^35^ were used to visualize all molecular interactions and the complex structure.

### Cell viability assay

B16F10 cells (ATCC-CRL-6475, USA) were cultured in Dulbecco’s modified eagle medium (DMEM) containing 10% fetal bovine serum (FBS), 100 U/mL penicillin, and 1 μg/mL streptomycin. Cell viability was evaluated using the MTT assay^36^. B16F10 cells (5 × 10^3^ cells/well) were cultured in a 96-well plate at 37 °C in a 5% CO_2_ incubator for 24 h. Tested compounds dissolved in DMSO/H_2_O (40% v/v) at different concentrations were added to sample wells, and the cells were further incubated at 37 °C in a 5% CO_2_ incubator for 48 h. Then, 50 µL of methylthiazolyldiphenyl-tetrazolium bromide (MTT) solution (0.5 mg/ml in 10 mM phosphate buffer, pH 7.4) was added to each well and left under a CO_2_ incubator for 2 h. The formazan crystals formed were dissolved in DMSO, and the absorbance was measured at 570 nm. Percent viability was calculated as the percent of surviving cells in the well compared to the control cells.

### Melanin content assay

The method for the melanin content assay was modified from Lee et al.^37^. B16F10 (8 × 10^4^ cell/mL) cells were seeded in a 6-well plate and incubated at 37 °C in a 5% CO_2_ incubator for 24 h. The tested and reference compounds in various concentrations were mixed with α-melanocyte stimulating hormone (α-MSH, 10 nM) and added to the well. The cells were continuously incubated at 37 °C for 48 h. Then, the cells were washed twice with 10 mM phosphate buffer pH 7.4. 1 M NaOH (0.2 mL) was added to each well and warmed at 65 °C for 1 h. The mixture was centrifuged at 12,000 rpm for 10 min. The absorbance of the supernatant was measured at 405 nm. The percent melanin content in the treatment group was obtained from the absorbance and calculated as a percentage compared to the untreated group.

### Statistical analyses

The data were expressed as means ± standard deviations (SD). Statistical analyses were performed using the t-test (two tails) in Microsoft Excel. A p-value < 0.05 was considered statistically significant.

### Supplementary Information

Supplementary Figures.

## Data Availability

All data generated or analyzed during this study are included in this published article [and its Supplementary Information files].
