# Supplementary material for: Synthesis and evaluation of coumarin derivatives on antioxidative, tyrosinase inhibitory activities, melanogenesis, and in silico investigations
Source: Sci Rep. 2024 Mar 6;14:5535. doi: 10.1038/s41598-024-54665-x (PMC10917816; doi:10.1038/s41598-024-54665-x)
Supplement: Supplementary file 1 — Supplementary Figures. [file 41598_2024_54665_MOESM1_ESM.pdf]

# Supplementary material

**Synthesis and evaluation of coumarin derivatives on antioxidative, tyrosinase inhibitory activities, melanogenesis, and *in silico* investigations**

Kasemsiri Chandarajoti[1,2], Jiraporn Kara[1,3], Paptawan Suwanhom[1,3], Teerapat Nualnoi[2,4], Jindaporn Puripattanavong[5], Vannajan Sanghiran Lee[6], Varomyalin Tipmanee[7] and Luelak Lomlim[1,3,*]

[1] Department of Pharmaceutical Chemistry, Faculty of Pharmaceutical Sciences, Prince of Songkla University, Hat Yai, Songkhla, 90112, Thailand; kasemsiri.c@psu.ac.th; 5210720005@psu.ac.th; luelak.l@psu.ac.th

[2] Drug Delivery System Excellence Center, Faculty of Pharmaceutical Sciences, Prince of Songkla University, Hat-Yai, Songkhla 90112, Thailand; kasemsiri.c@psu.ac.th, teerapat.n@psu.ac.th

[3] Phytomedicine and Pharmaceutical Biotechnology Excellent Center (PPBEC), Faculty of Pharmaceutical Sciences, Prince of Songkla University, Songkhla, 90112, Thailand; 5210720005@psu.ac.th, luelak.l@psu.ac.th

[4] Department of Pharmaceutical Technology, Faculty of Pharmaceutical Sciences, Prince of Songkla University, Hat Yai, Songkhla, 90112, Thailand; teerapat.n@psu.ac.th

[5] Department of Pharmacognosy and Pharmaceutical Botany, Faculty of Pharmaceutical Sciences, Prince of Songkla University, Hat Yai, Songkhla, 90112, Thailand; jindaporn.p@psu.ac.th

[6] Department of Chemistry, Faculty of Science, University of Malaya, Kuala Lumpur, 50603, Malaysia; vannajan@um.edu.my

[7] Department of Biomedical Sciences and Biomedical Engineering, Faculty of Medicine, Prince of Songkla University, Songkhla 90112, Thailand; tvaromya@medicine.psu.ac.th.

Running title: Coumarin derivatives as tyrosinase modulators

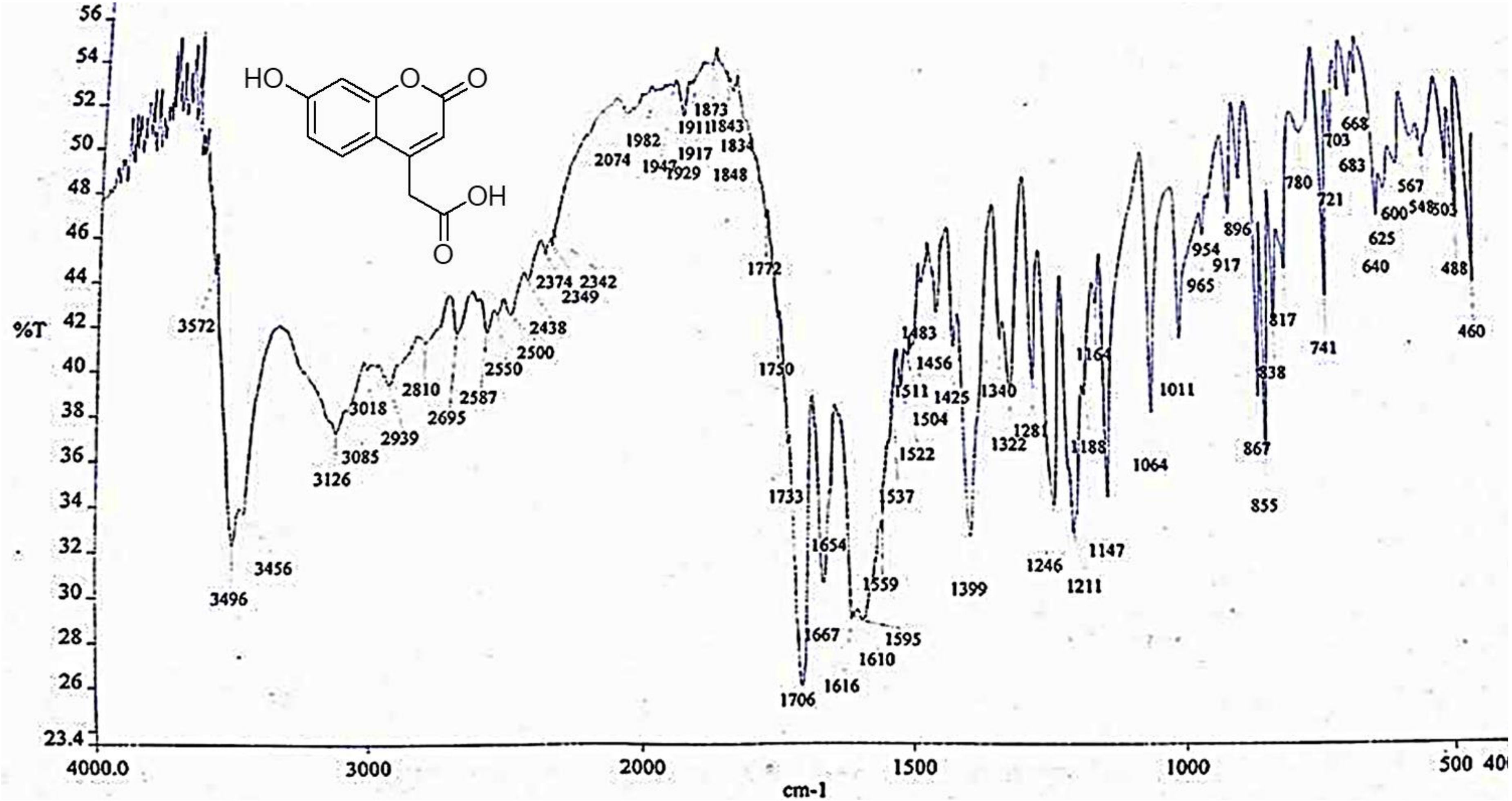


**Figure S-1** IR Spectra of 2-(7-hydroxy-2-oxo-2H-chromen-4-yl)acetic acid (**4**)

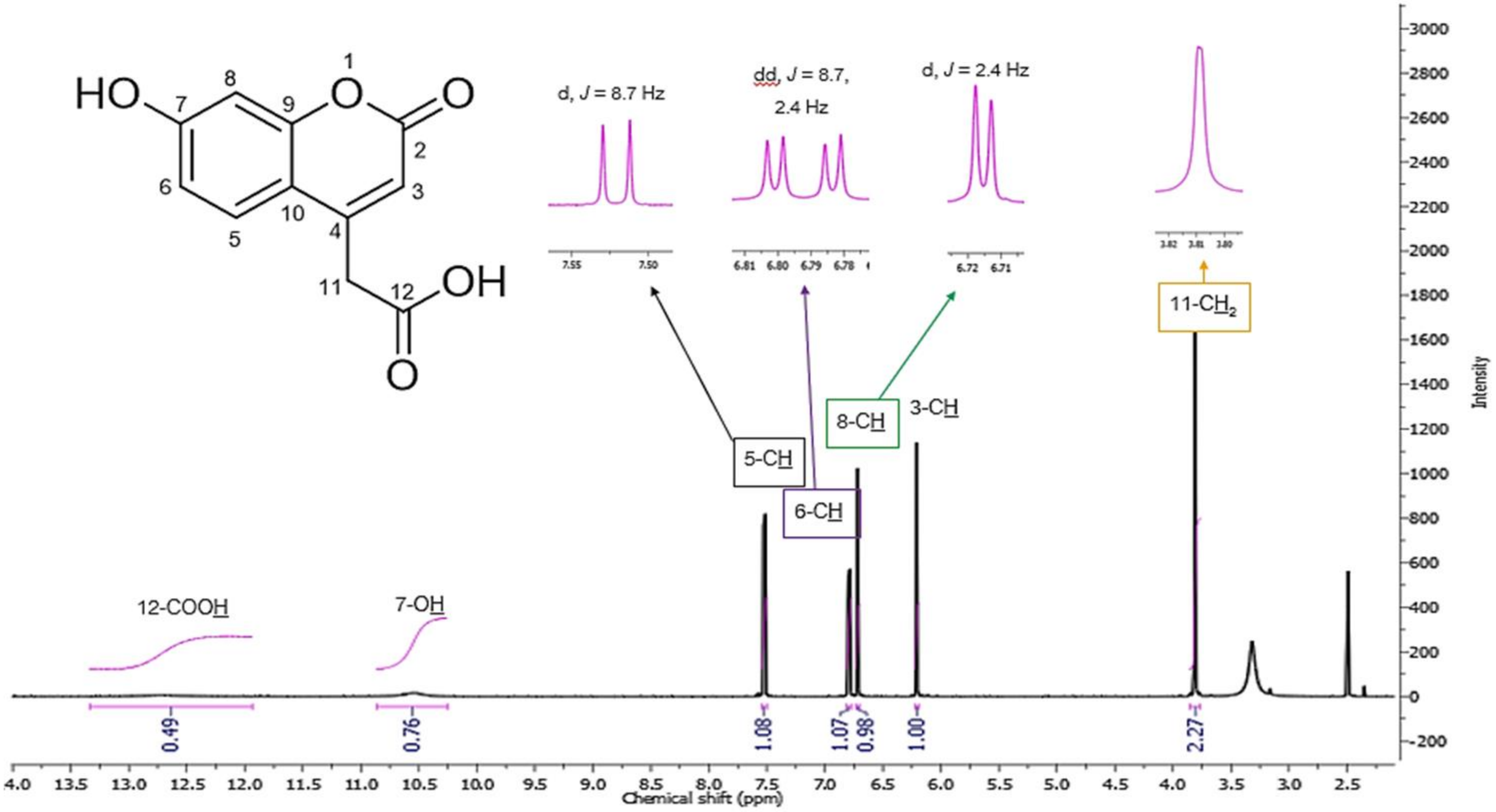


**Figure S-2** $^{1}$H-NMR spectra of 2-(7-hydroxy-2-oxo-2H-chromen-4-yl)acetic acid (**4**) in DMSO-$d_6$

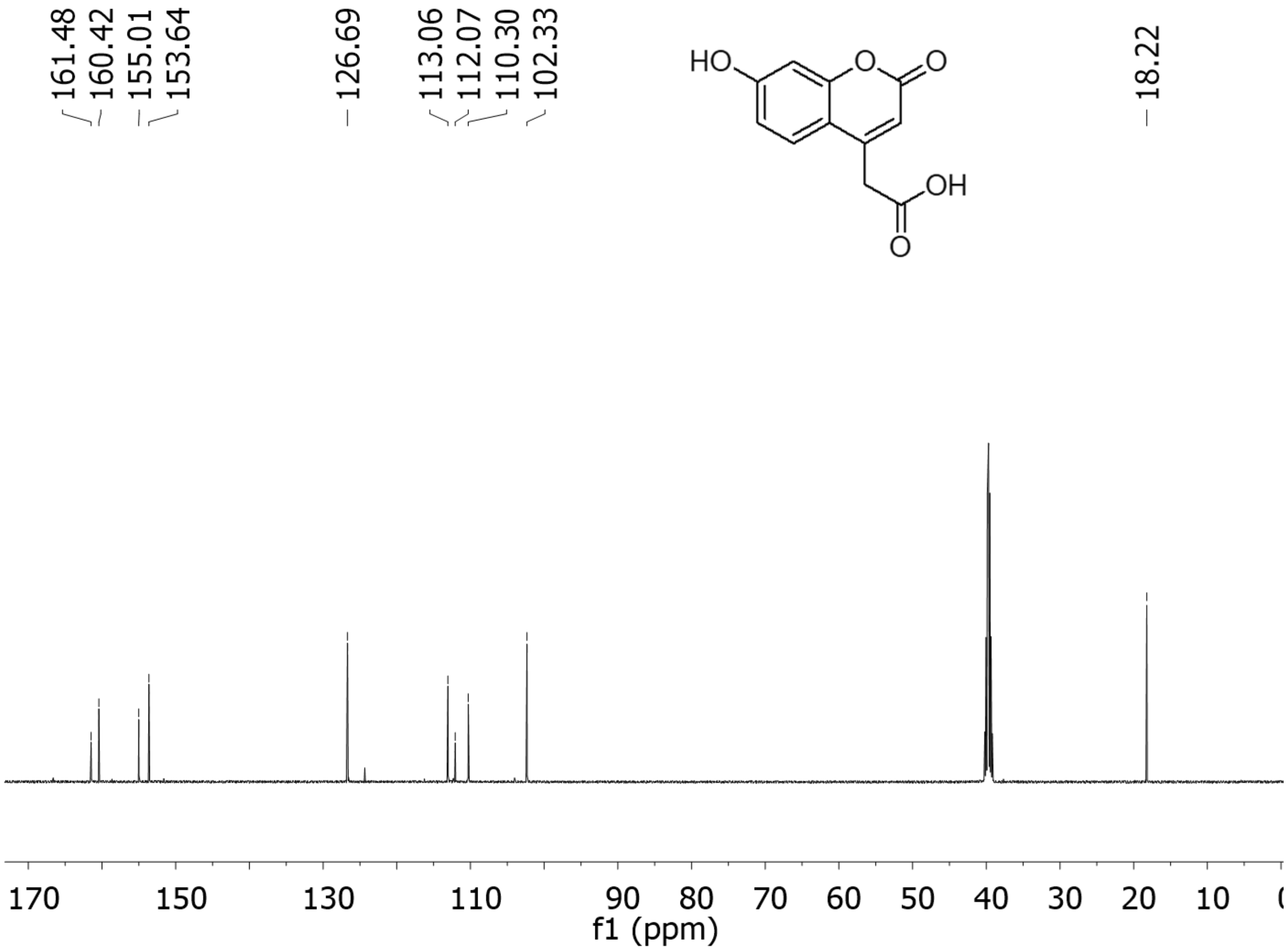


**Figure S-3** $^{13}$C-NMR spectra of 2-(7-hydroxy-2-oxo-2H-chromen-4-yl)acetic acid (**4**) in DMSO-$d_6$

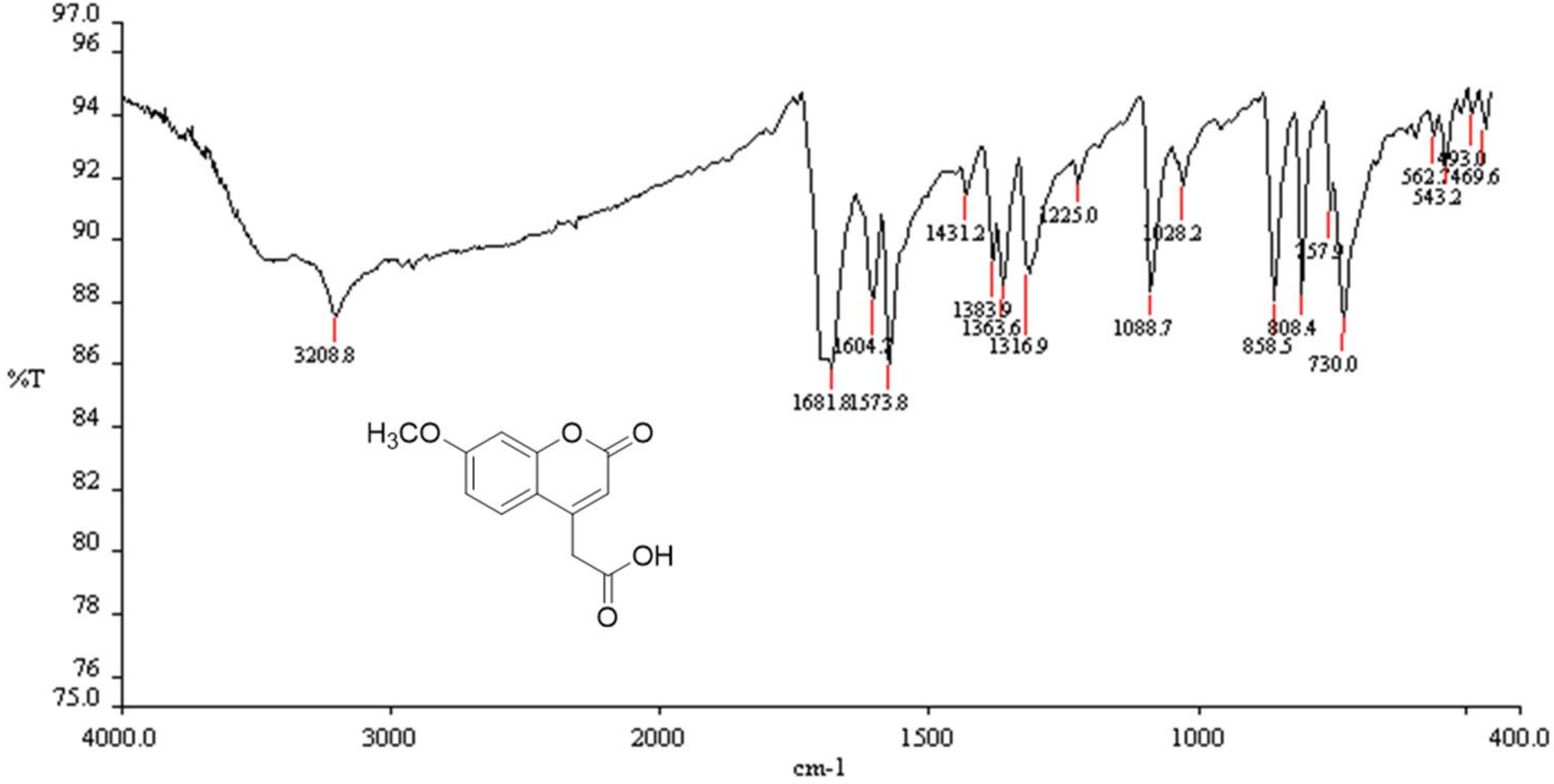


**Figure S-4** IR Spectra of 2-(7-methoxy-2-oxo-2H-chromen-4-yl)acetic acid (**5**)

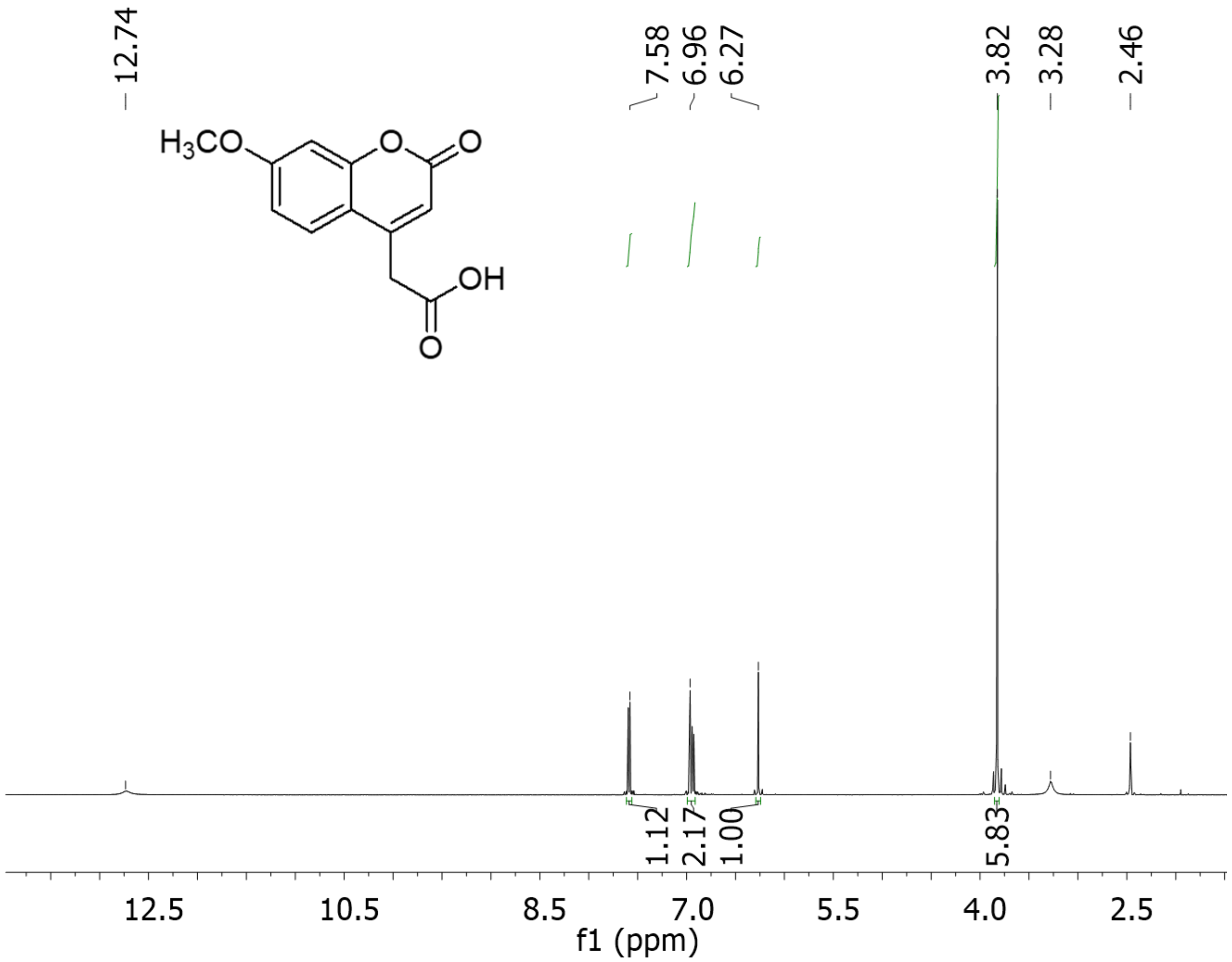


**Figure S-5** $^{1}$H-NMR spectra of 2-(7-methoxy-2-oxo-2H-chromen-4-yl)acetic acid (**5**) in DMSO-*d6*

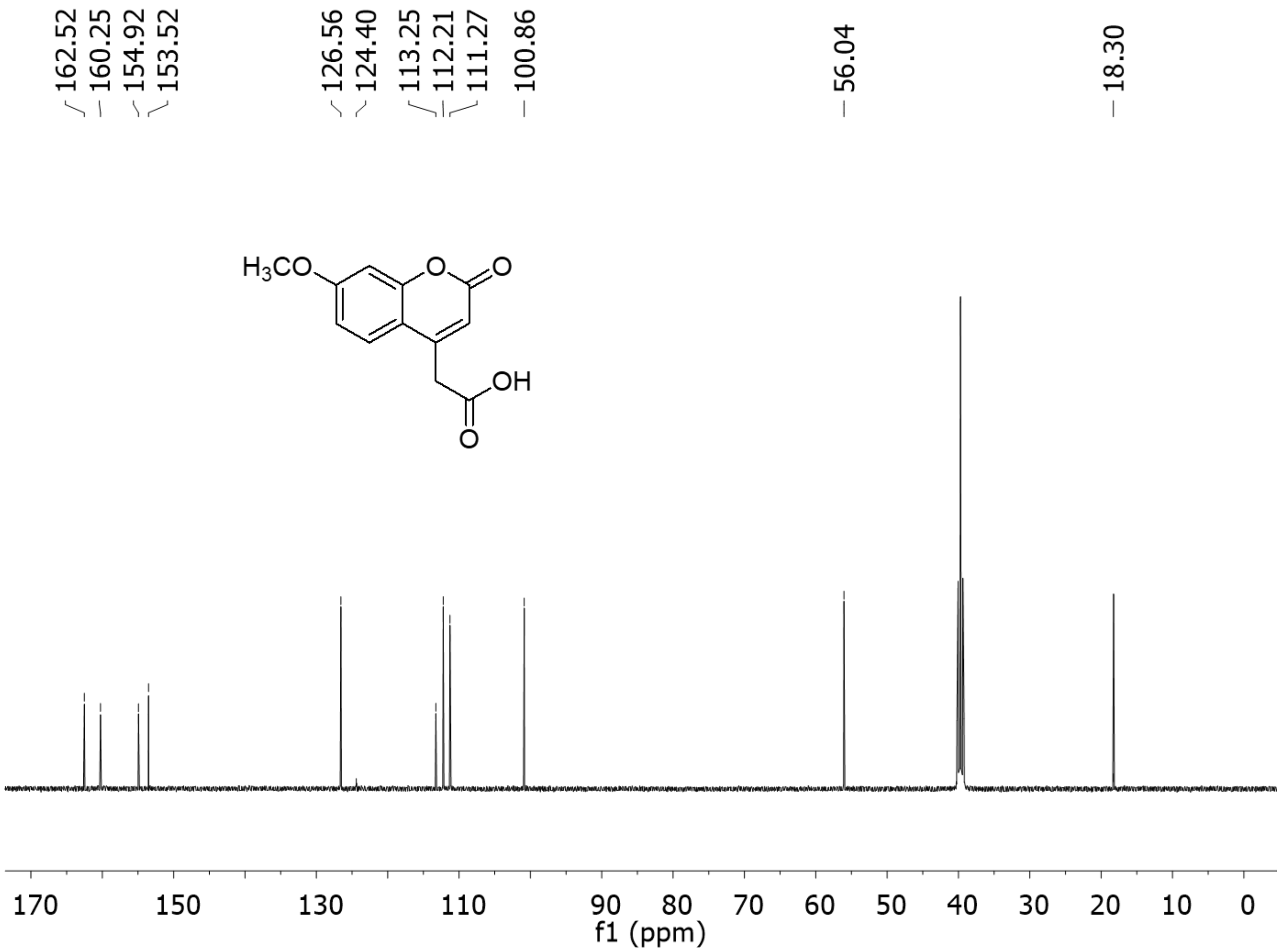


**Figure S-6** $^{13}$C-NMR spectra of 2-(7-methoxy-2-oxo-2H-chromen-4-yl)acetic acid (**5**) in DMSO-$d_6$

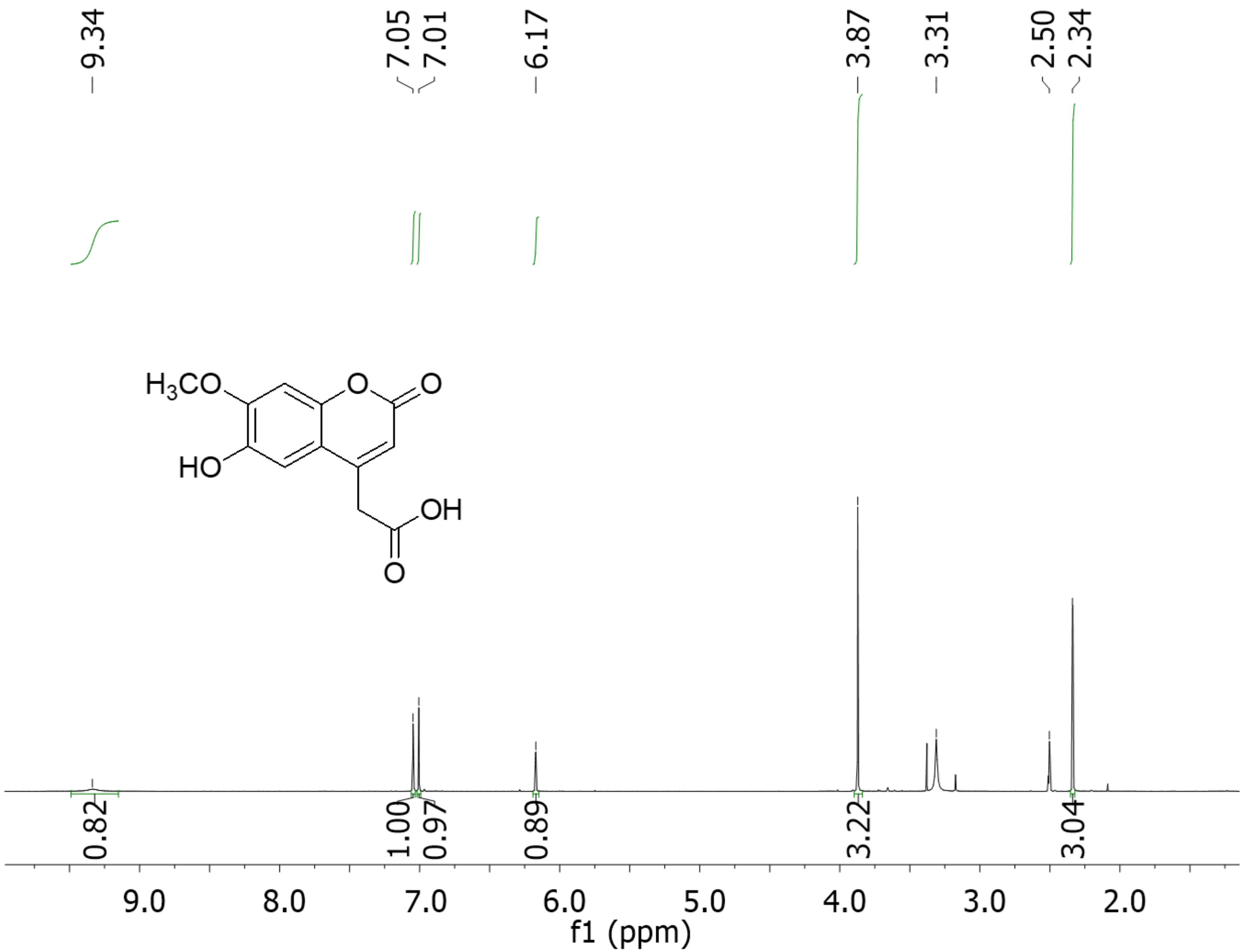


**Figure S-7** $^1$H-NMR spectra of 2-(6-hydroxy-7-methoxy-2-oxo-2H-chromen-4-yl)acetic acid (**6**) in DMSO-$d_6$

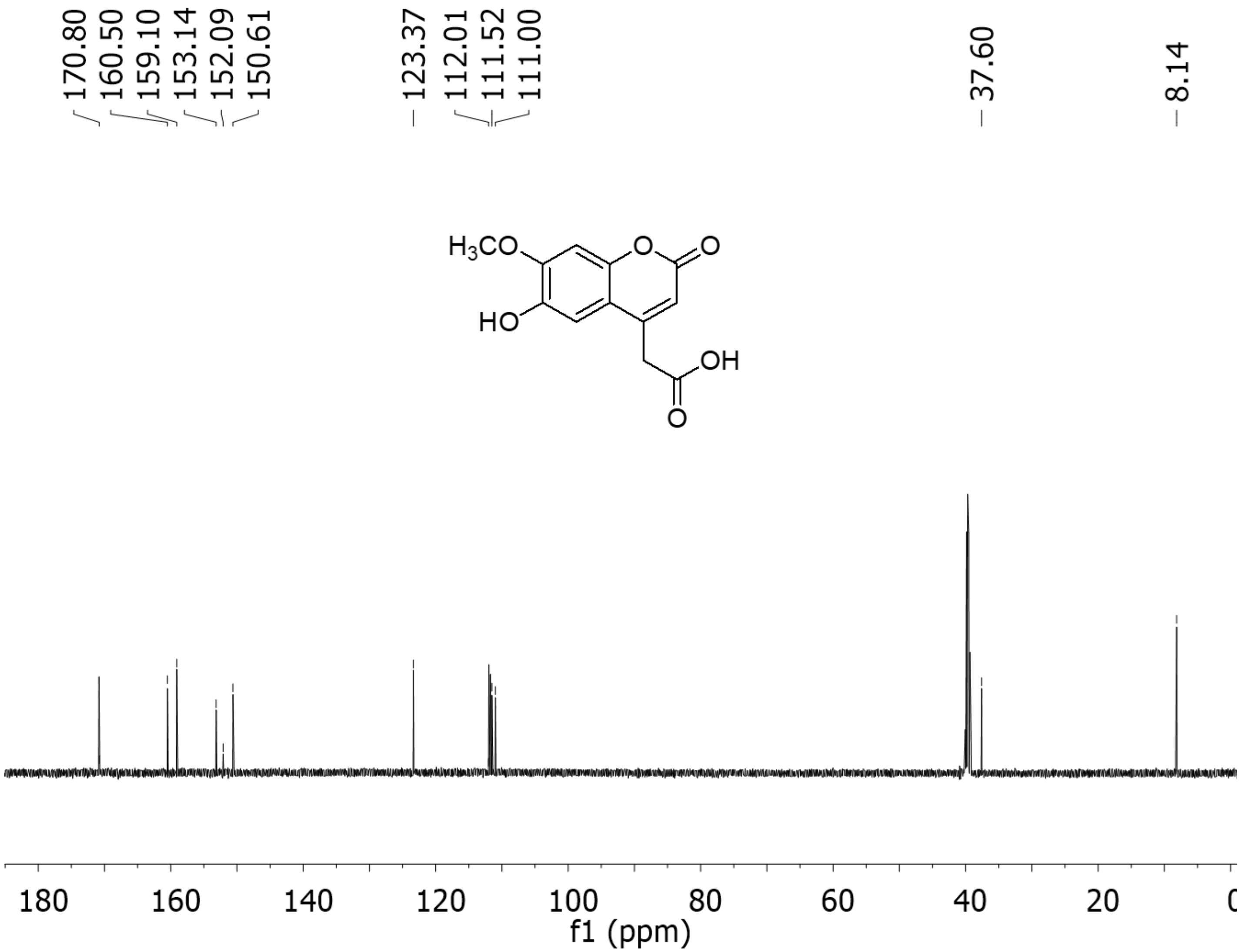


**Figure S-8** $^{13}$C-NMR of 2-(6-hydroxy-7-methoxy-2-oxo-2H-chromen-4-yl)acetic acid (**6**) **in** DMSO-$d_6$

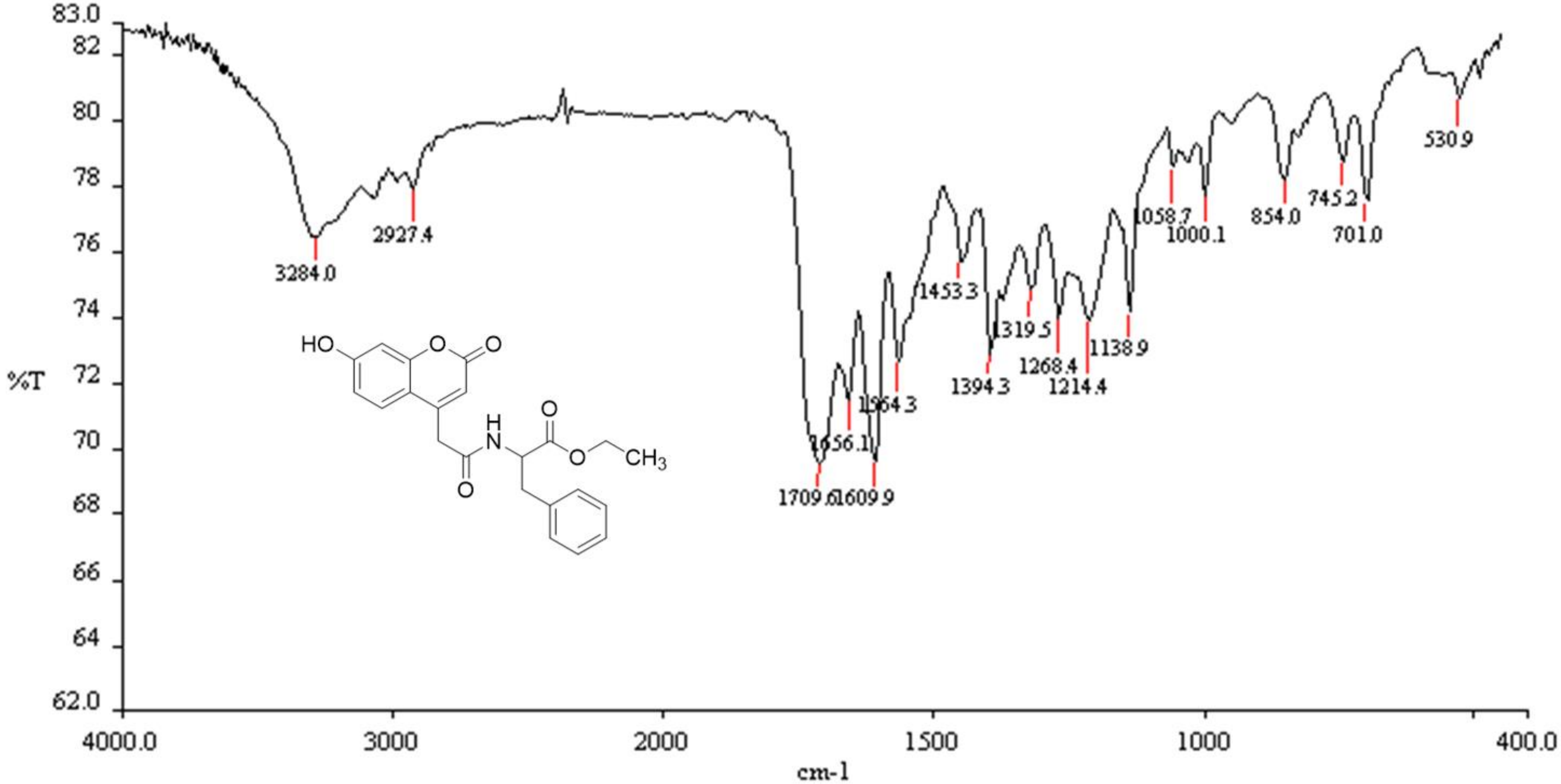


**Figure S-9** IR Spectra of Ethyl 2-(2-(7-hydroxy-2-oxo-2H-chromen-4-yl)acetamido)-3-phenylpropanoate (**7**)

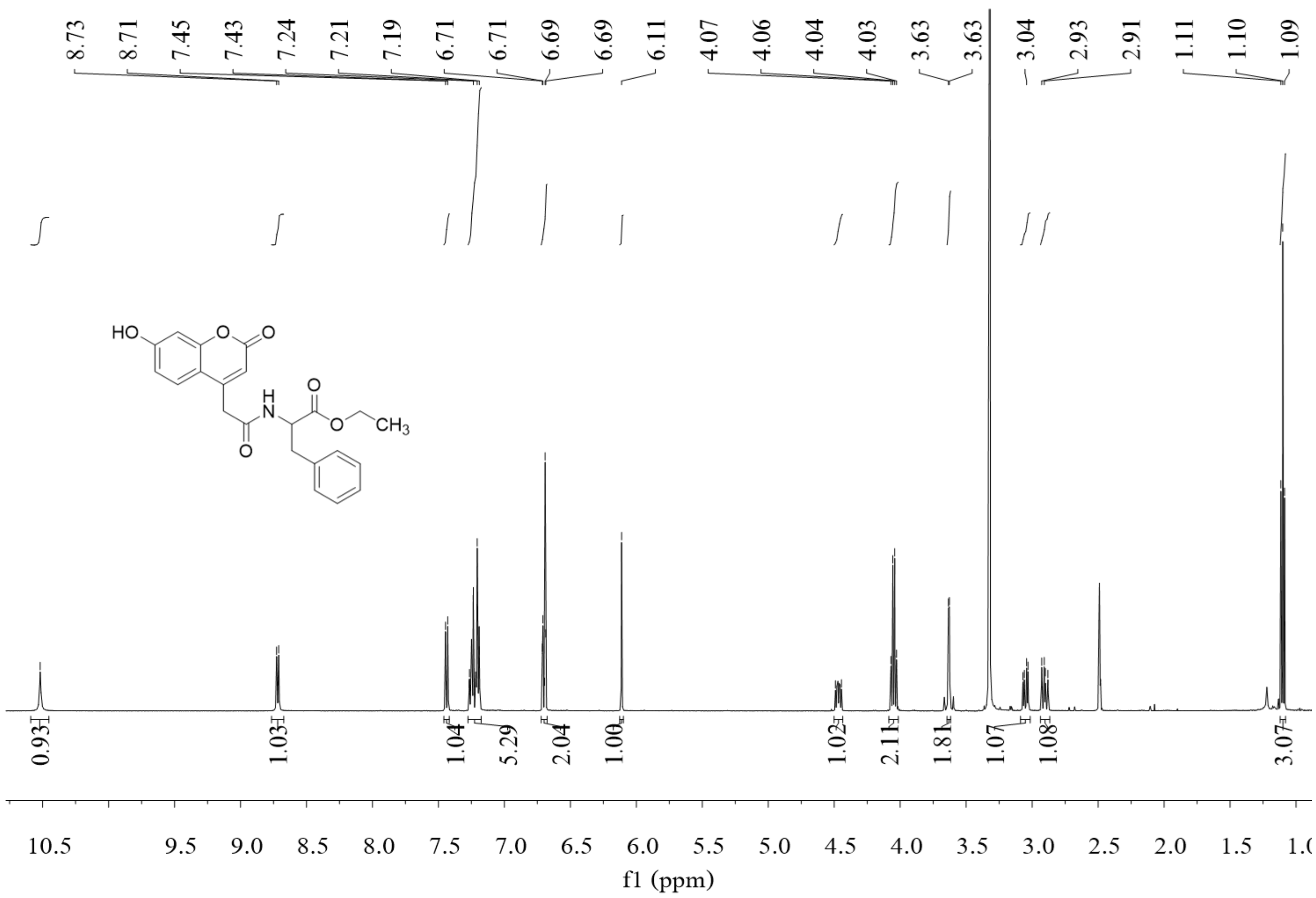


**Figure S-10** $^{1}$H-NMR spectra of Ethyl 2-(2-(7-hydroxy-2-oxo-2H-chromen-4-yl)acetamido)-3-phenylpropanoate (**7**) in DMSO-$d_6$

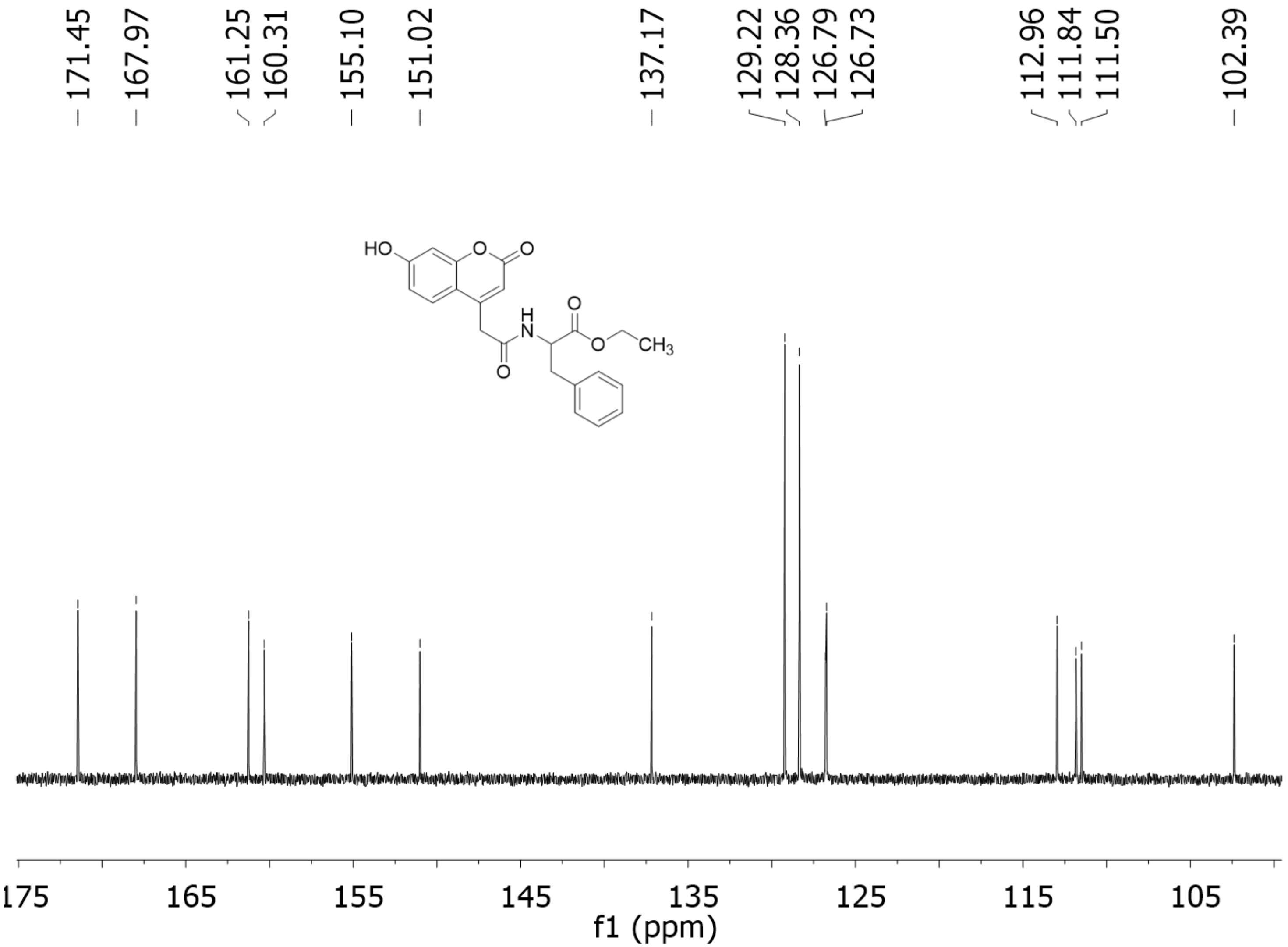


**Figure S-11** $^{13}$C-NMR spectra of Ethyl 2-(2-(7-hydroxy-2-oxo-2H-chromen-4-yl)acetamido)-3-phenylpropanoate (**7**) in DMSO-$d_6$

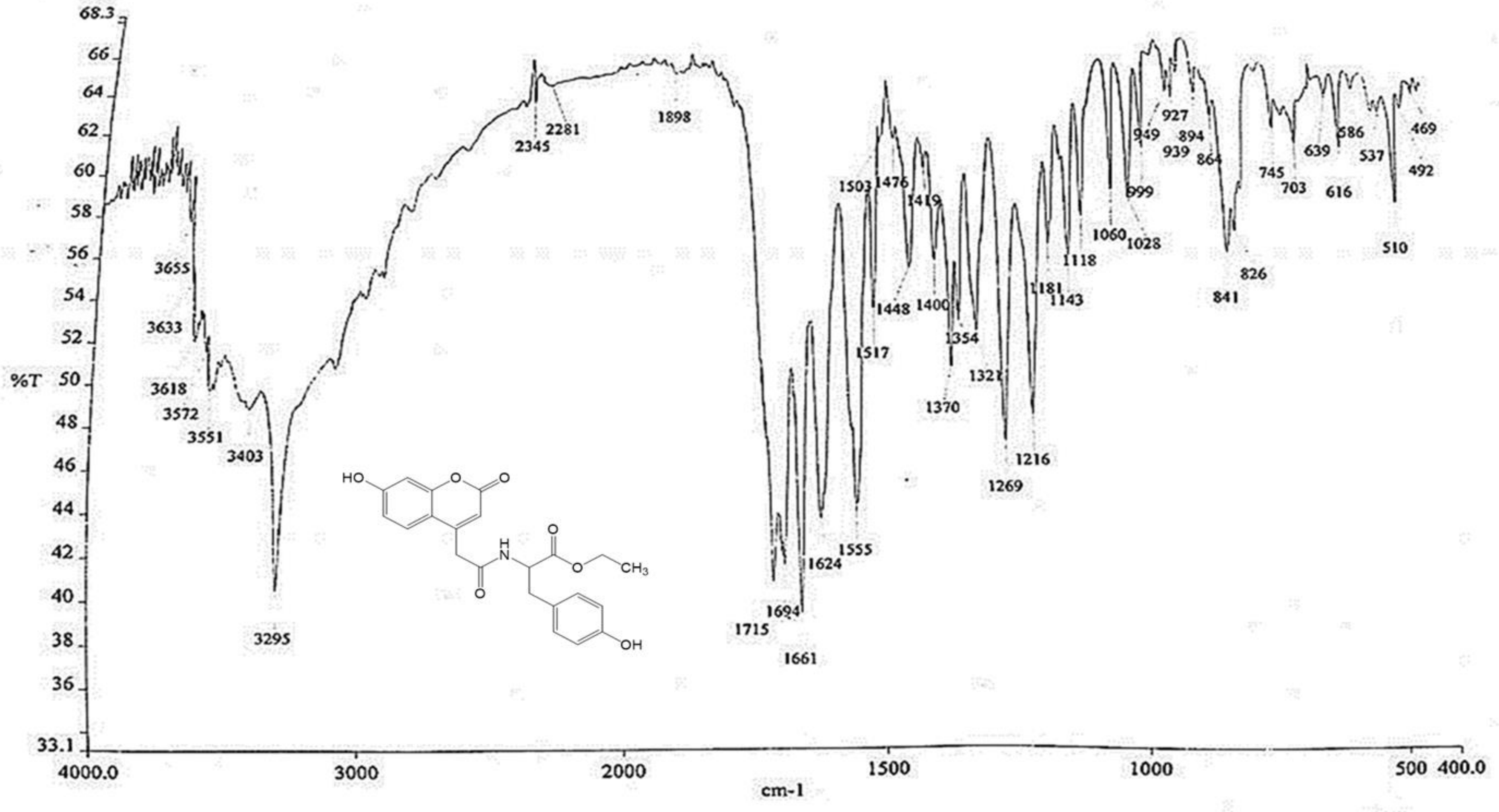


**Figure S-12** IR Spectra of Ethyl 2-(2-(7-hydroxy-2-oxo-2H-chromen-4-yl)acetamido)-3-(4-hydroxyphenyl)propanoate (**8**)

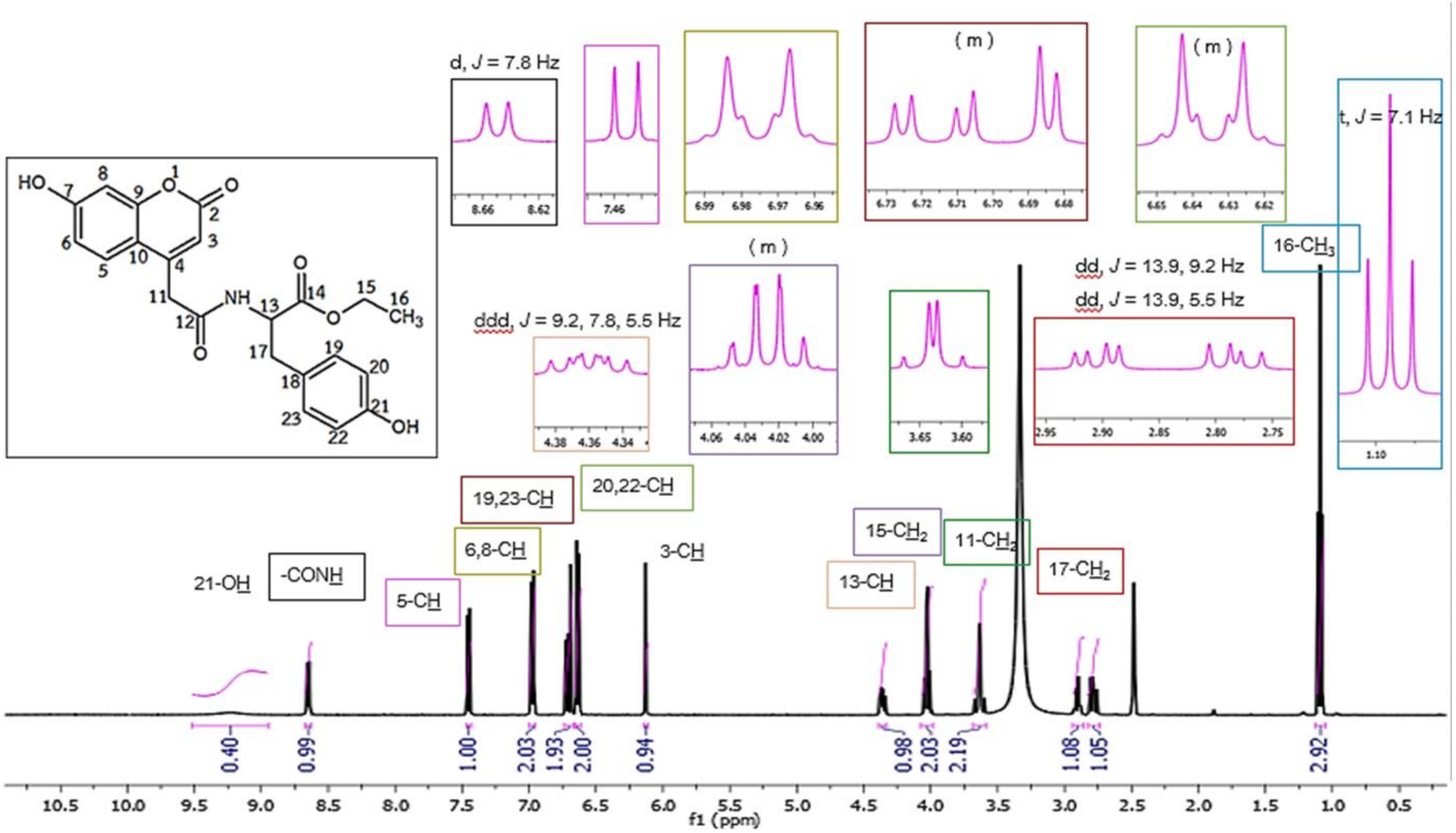


**Figure S-13** $^{1}$H-NMR spectra of Ethyl 2-(2-(7-hydroxy-2-oxo-2H-chromen-4-yl)acetamido)-3-(4-hydroxyphenyl)propanoate (**8**) in DMSO-$d_6$

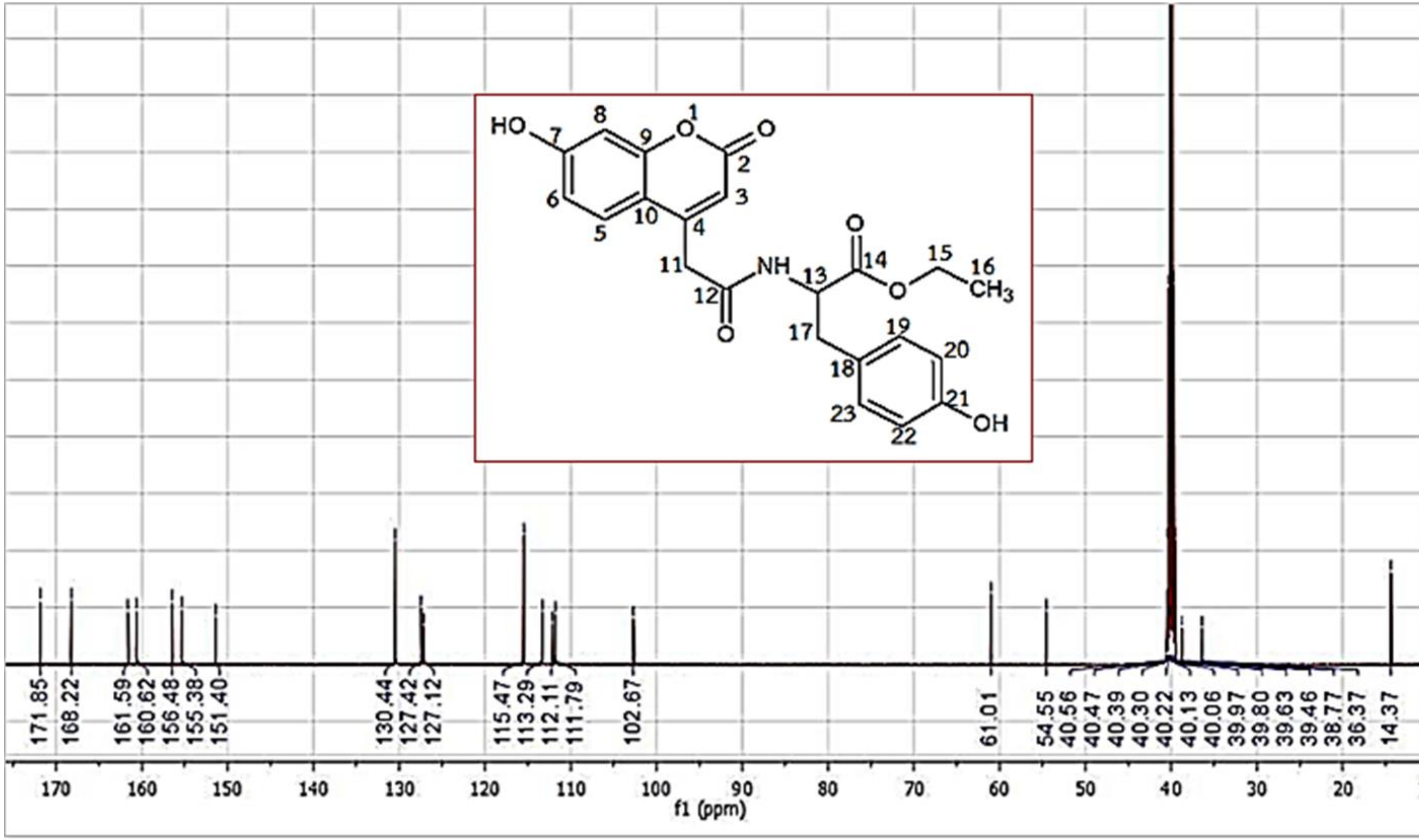


**Figure S-14** $^{13}$C-NMR of Ethyl 2-(2-(7-hydroxy-2-oxo-2H-chromen-4-yl)acetamido)-3-(4-hydroxyphenyl)propanoate (**8**) in DMSO-$d_6$

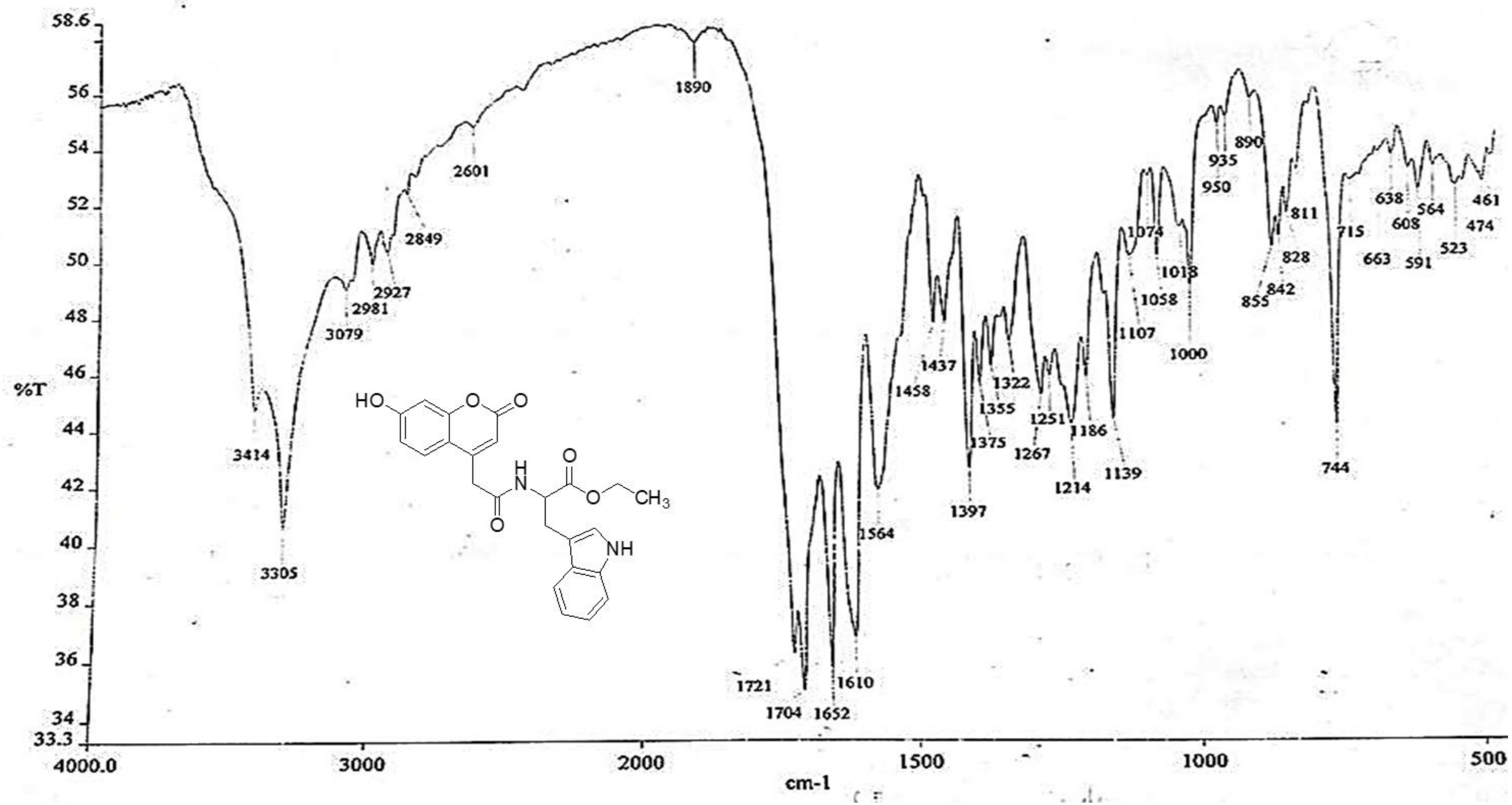


**Figure S-15** IR Spectra ของ Ethyl 2-(2-(7-hydroxy-2-oxo-2H-chromen-4-yl)acetamido)-3-(1H-indol-2-yl)propanoate (**9**)

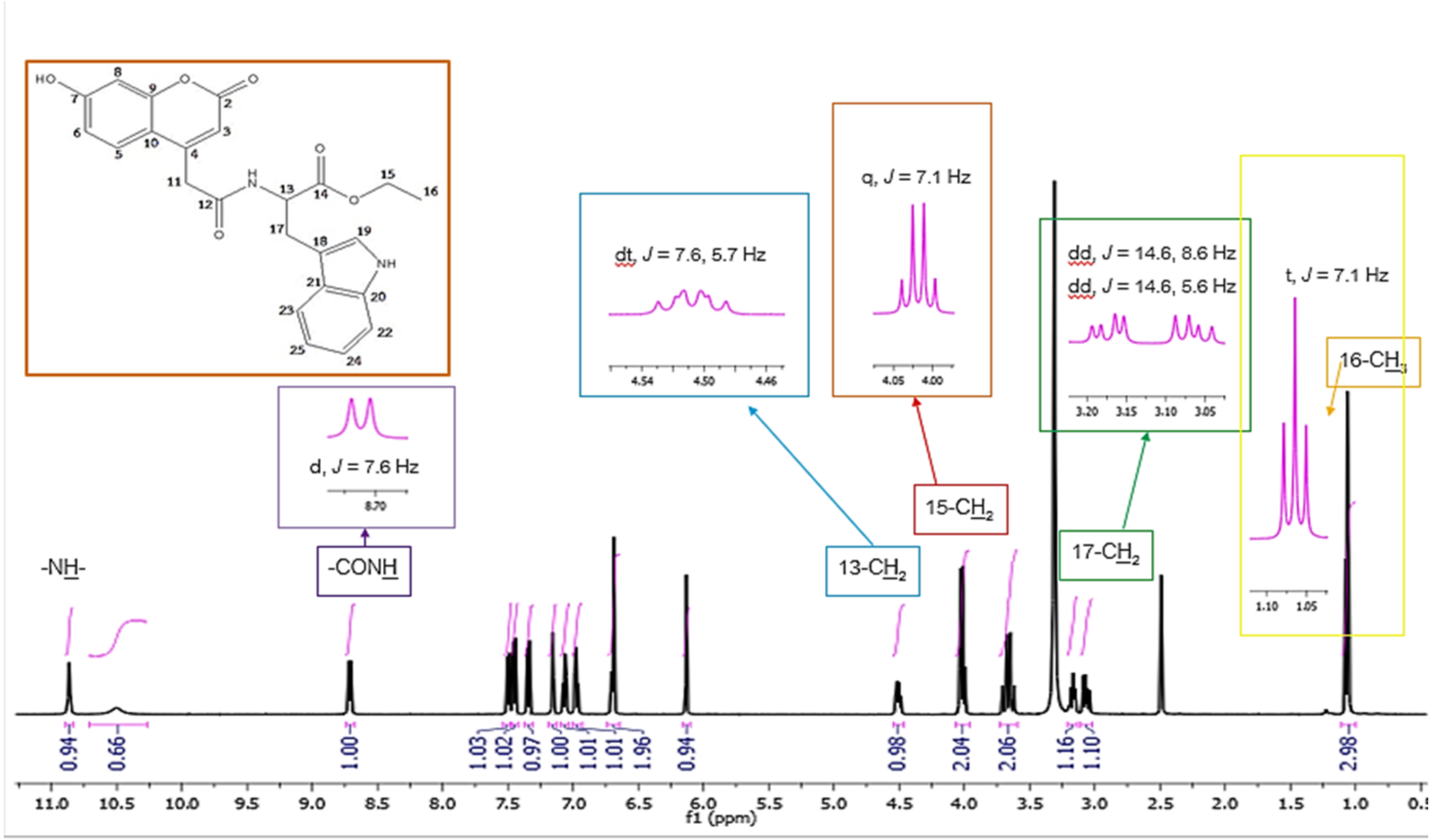


**Figure S-16** $^{1}$H-NMR spectra of Ethyl 2-(2-(7-hydroxy-2-oxo-2H-chromen-4-yl)acetamido)-3-(1H-indol-2-yl)propanoate (**9**) in DMSO-$d_6$

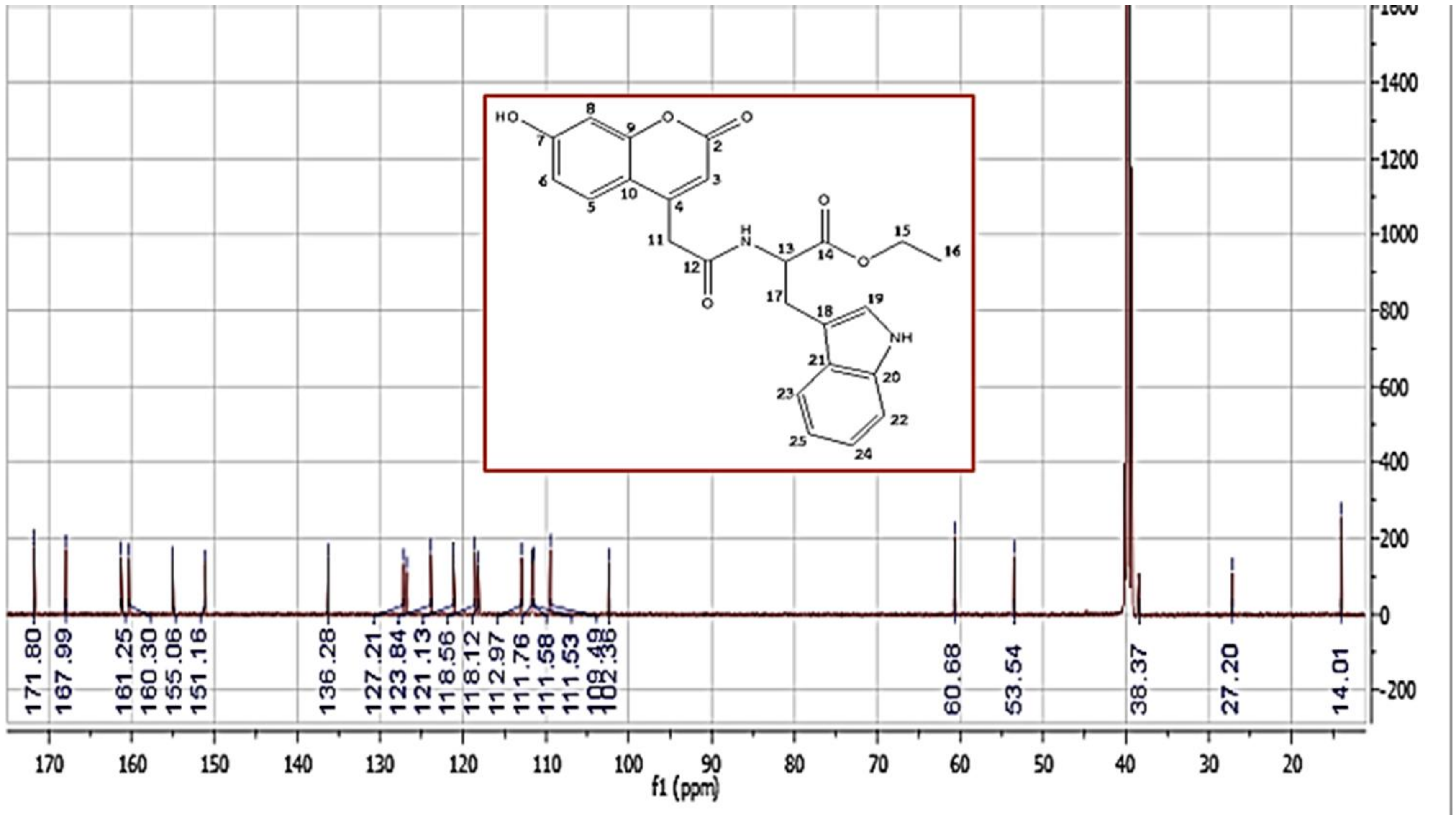


**Figure S-17** $^{13}$C-NMR spectra of Ethyl 2-(2-(7-hydroxy-2-oxo-2H-chromen-4-yl)acetamido)-3-(1H-indol-2-yl)propanoate (**9**) in DMSO-$d_6$

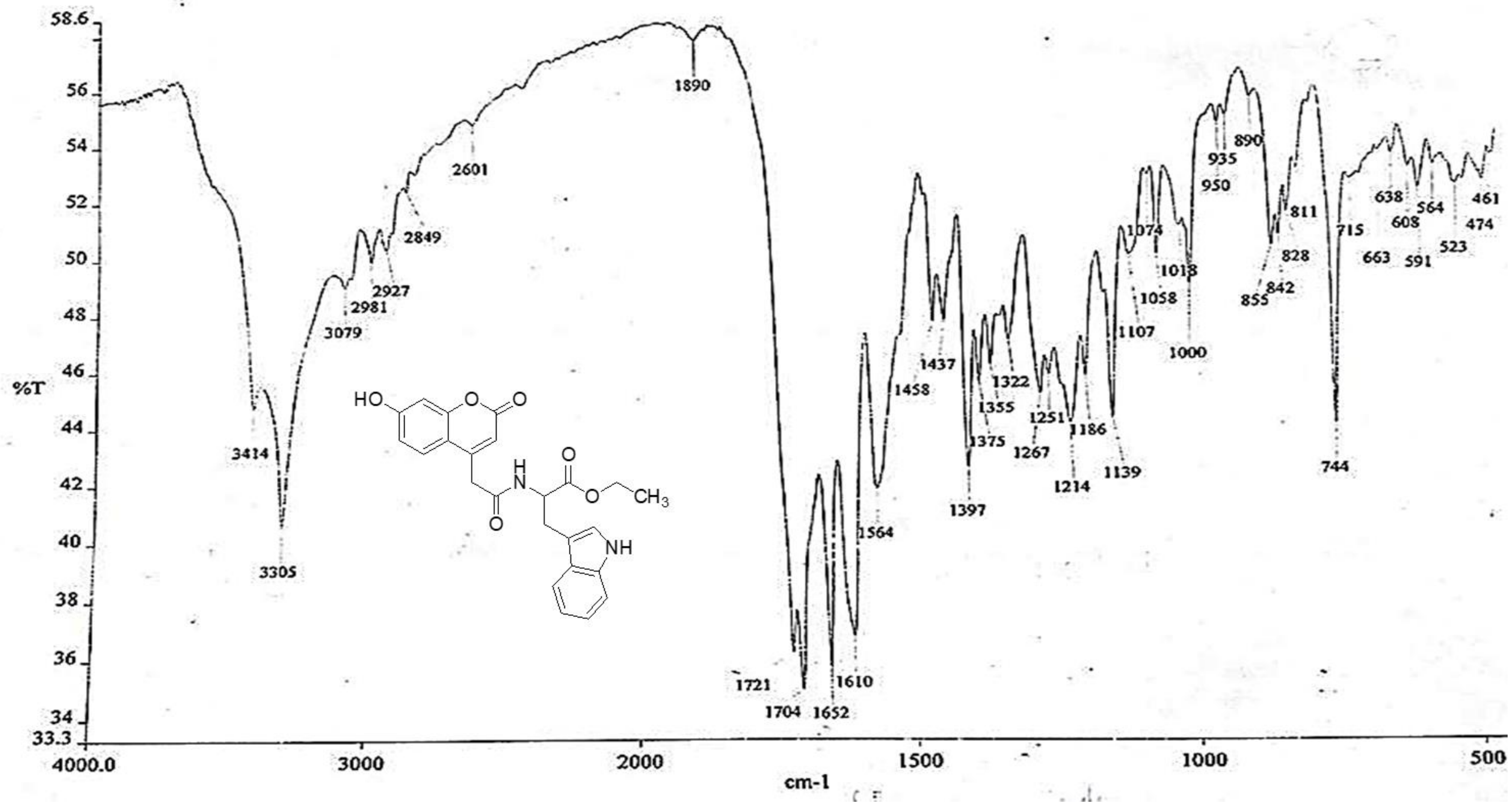


**Figure S-18** IR Spectra of N-(4-hydroxyphenethyl)-2-(7-hydroxy-2-oxo-2H-chromen-4-yl)acetamide (**10**)

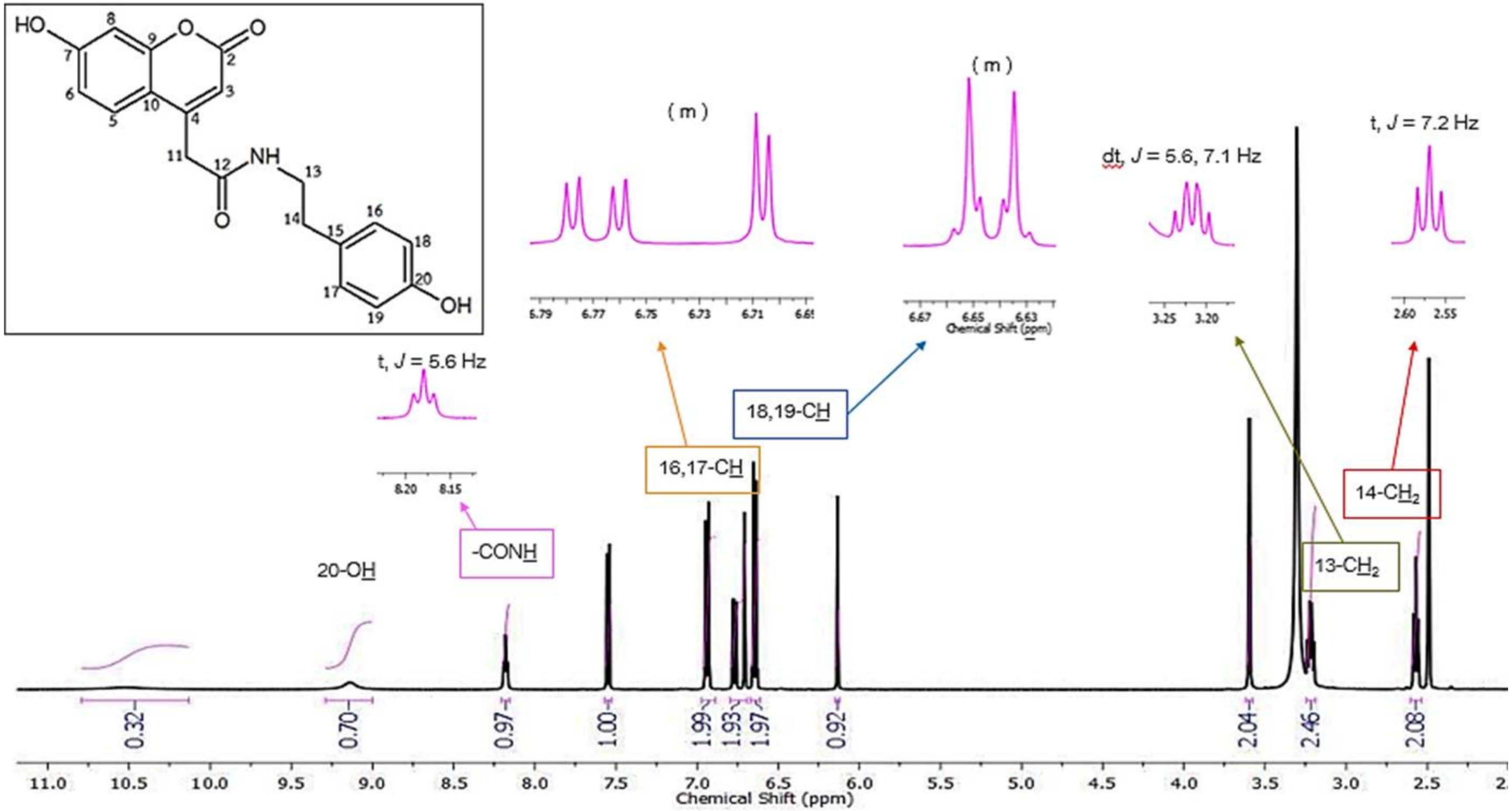


**Figure S-19** $^{1}$H-NMR spectra of N-(4-hydroxyphenethyl)-2-(7-hydroxy-2-oxo-2H-chromen-4-yl)acetamide (**10**) in DMSO-$d_6$

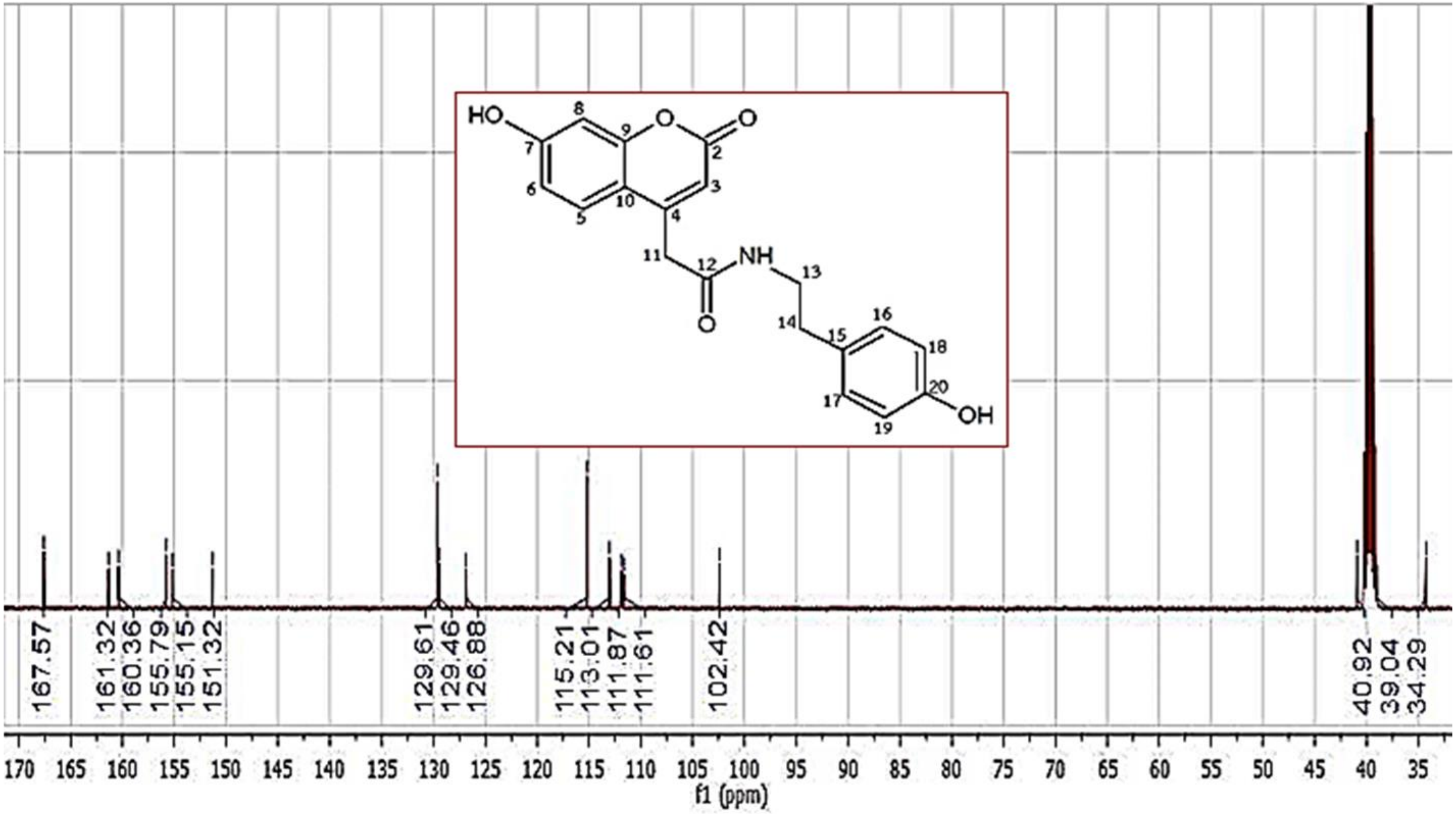


**Figure S-20** $^{13}$C-NMR spectra of N-(4-hydroxyphenethyl)-2-(7-hydroxy-2-oxo-2H-chromen-4-yl)acetamide (**10**) in DMSO-$d_6$

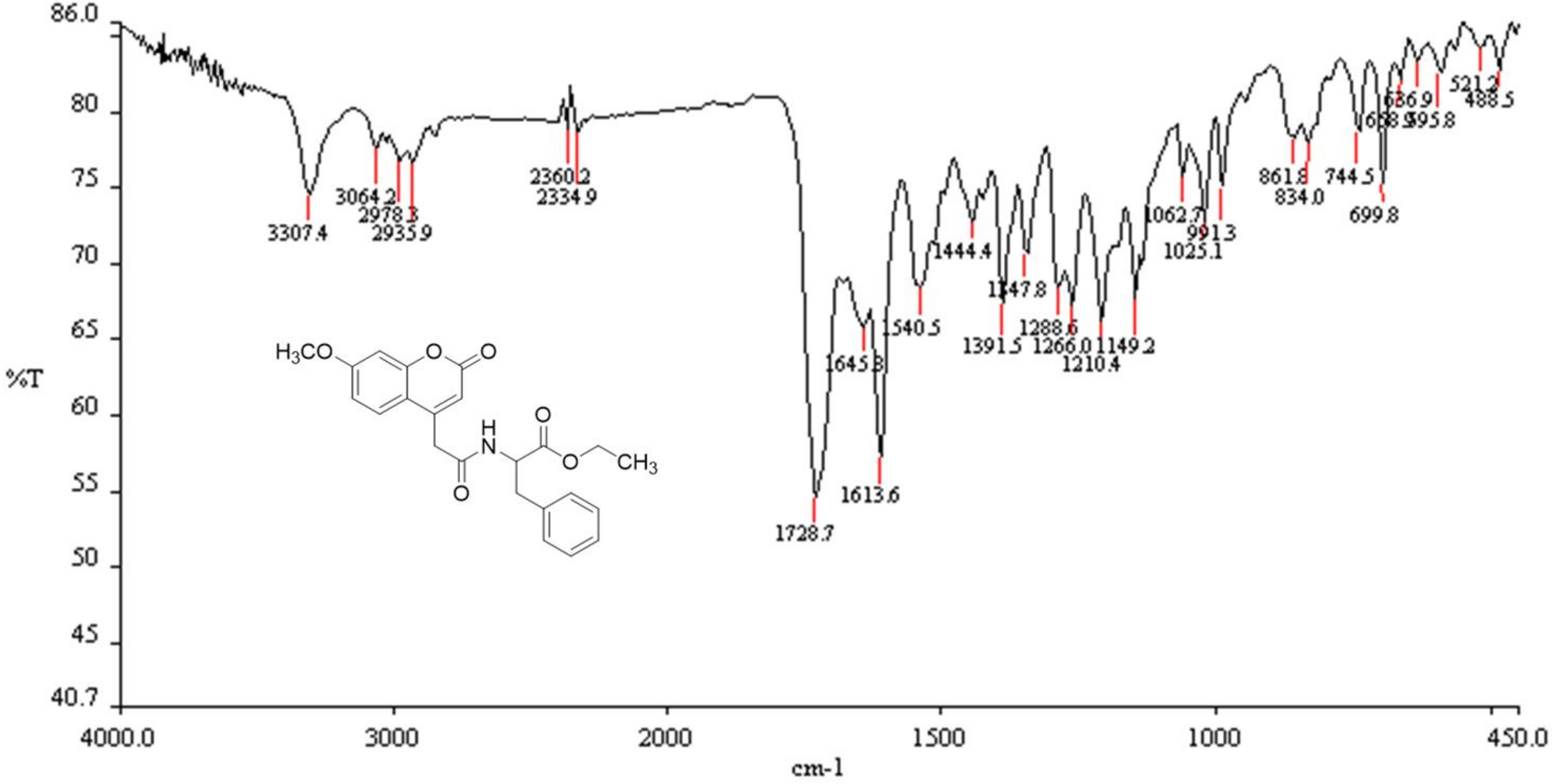


**Figure S-21** IR Spectra of Ethyl 2-(2-(7-methoxy-2-oxo-2H-chromen-4-yl)acetamido)-3-phenylpropanoate (**11**)

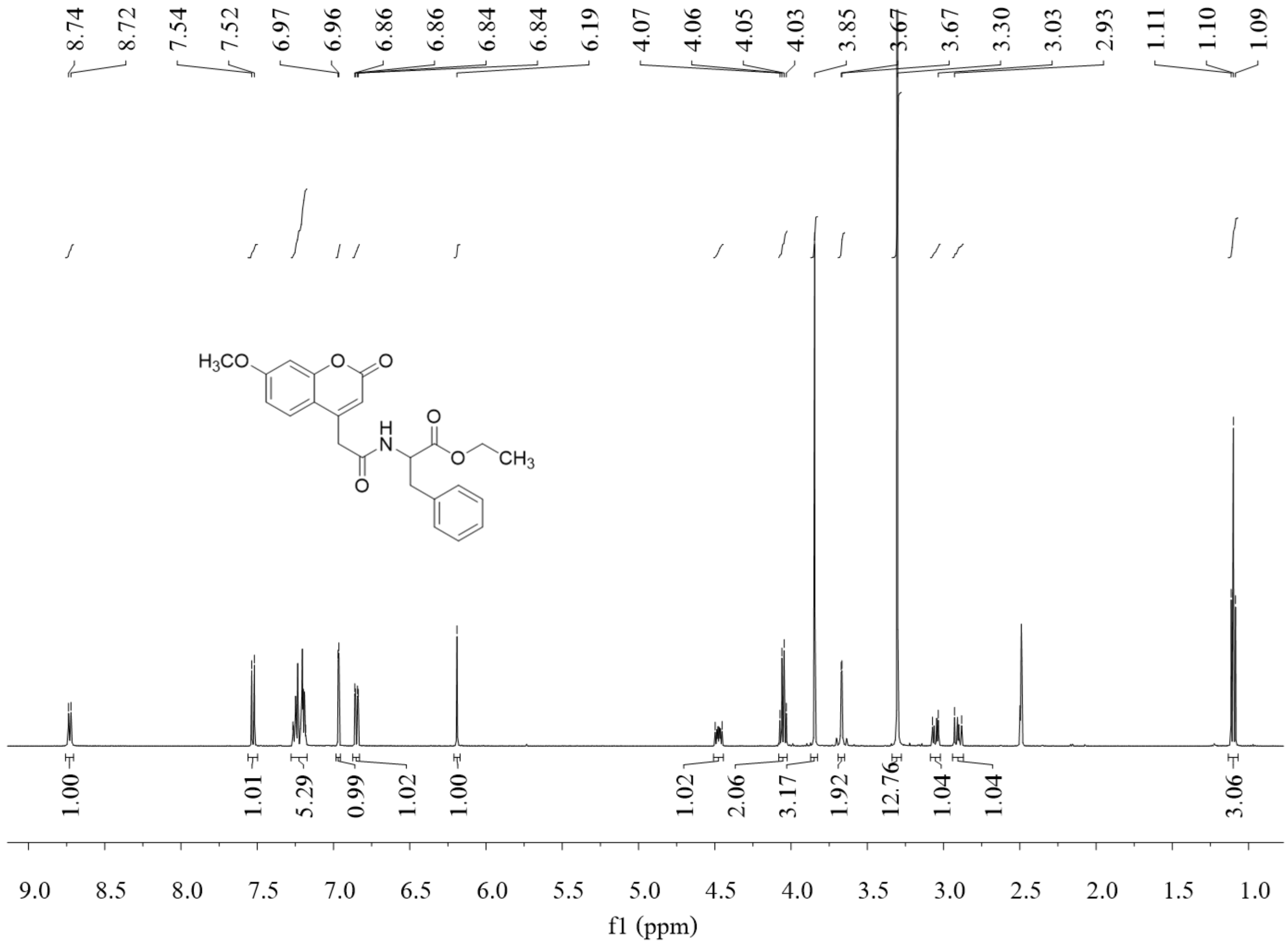


**Figure S-22** $^{1}$H-NMR spectra of Ethyl 2-(2-(7-methoxy-2-oxo-2H-chromen-4-yl)acetamido)-3-phenylpropanoate (**11**) in DMSO-$d_6$

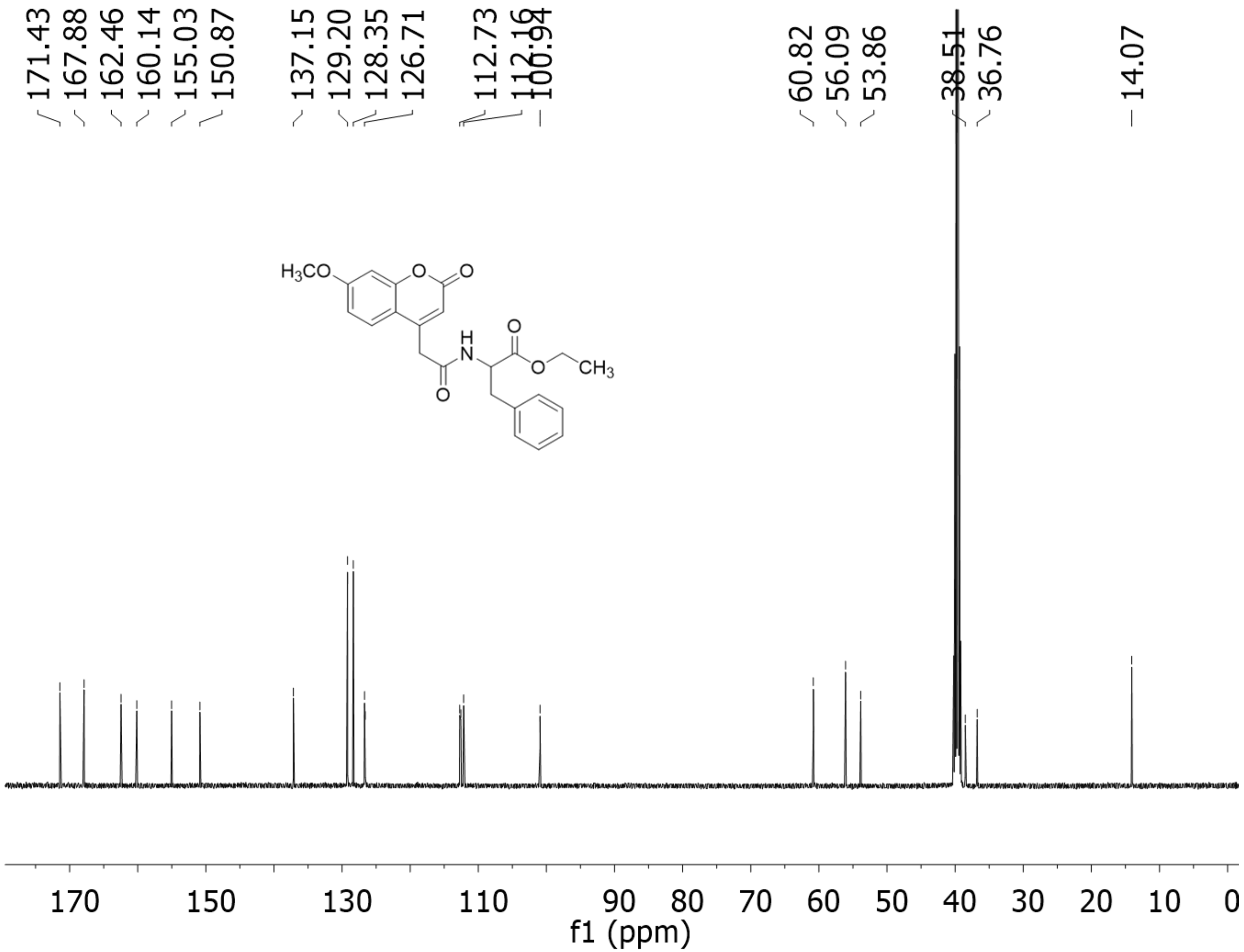


**Figure S-23** $^{13}$C-NMR spectra of Ethyl 2-(2-(7-methoxy-2-oxo-2H-chromen-4-yl)acetamido)-3-phenylpropanoate (**11**) in DMSO-$d_6$

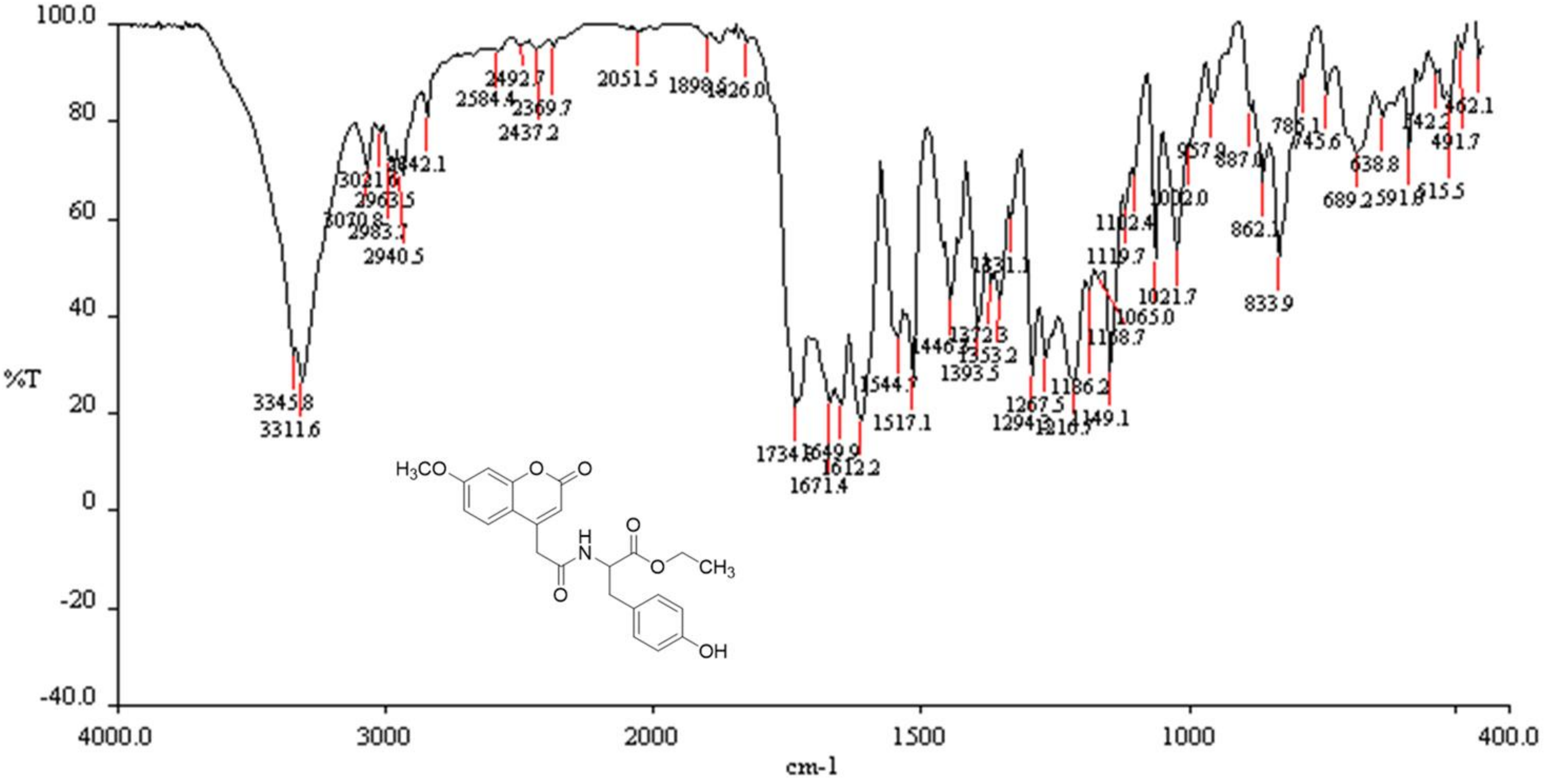


**Figure S-24** IR Spectra of Ethyl 2-(2-(7-methoxy-2-oxo-2H-chromen-4-yl)acetamido)-3-(4-hydroxyphenyl) propanoate (**12**)

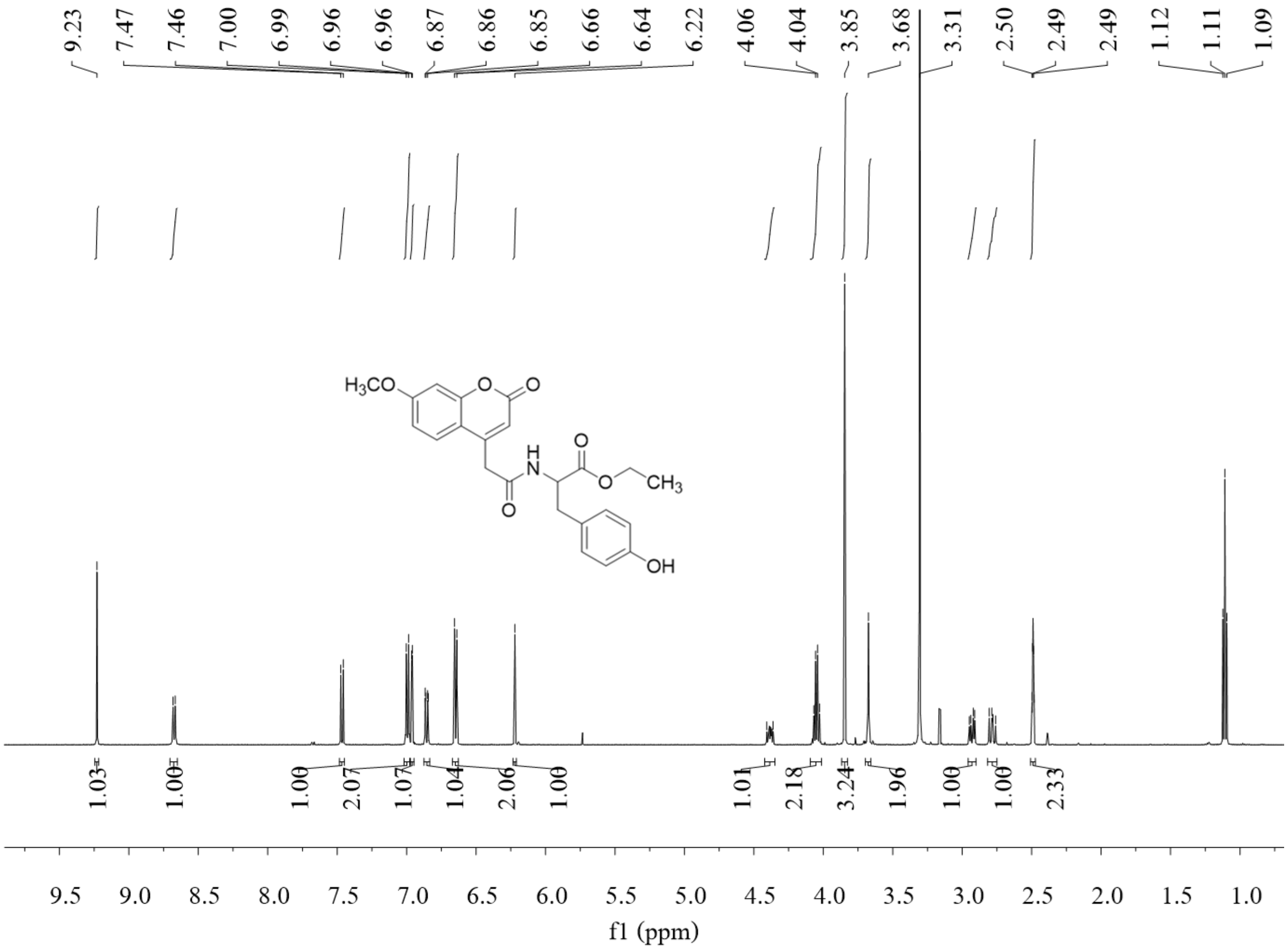


**Figure S-25** $^{1}$H-NMR spectra of Ethyl 2-(2-(7-methoxy-2-oxo-2H-chromen-4-yl)acetamido)-3-(4-hydroxyphenyl) propanoate (**12**) in DMSO-$d_6$

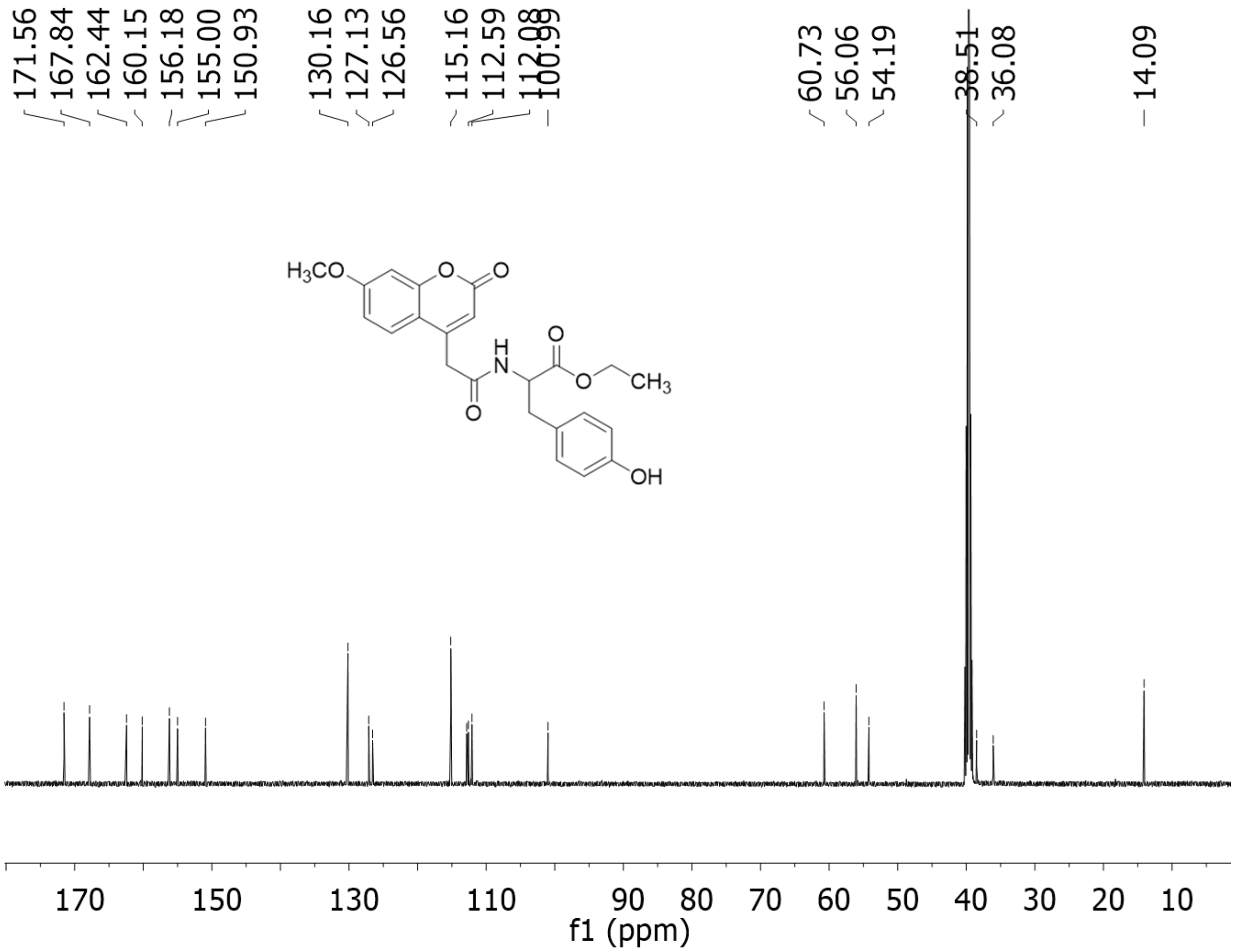


**Figure S-26** $^{13}$C-NMR spectra of Ethyl 2-(2-(7-methoxy-2-oxo-2H-chromen-4-yl)acetamido)-3-(4-hydroxyphenyl) propanoate (**12**) in DMSO-$d_6$

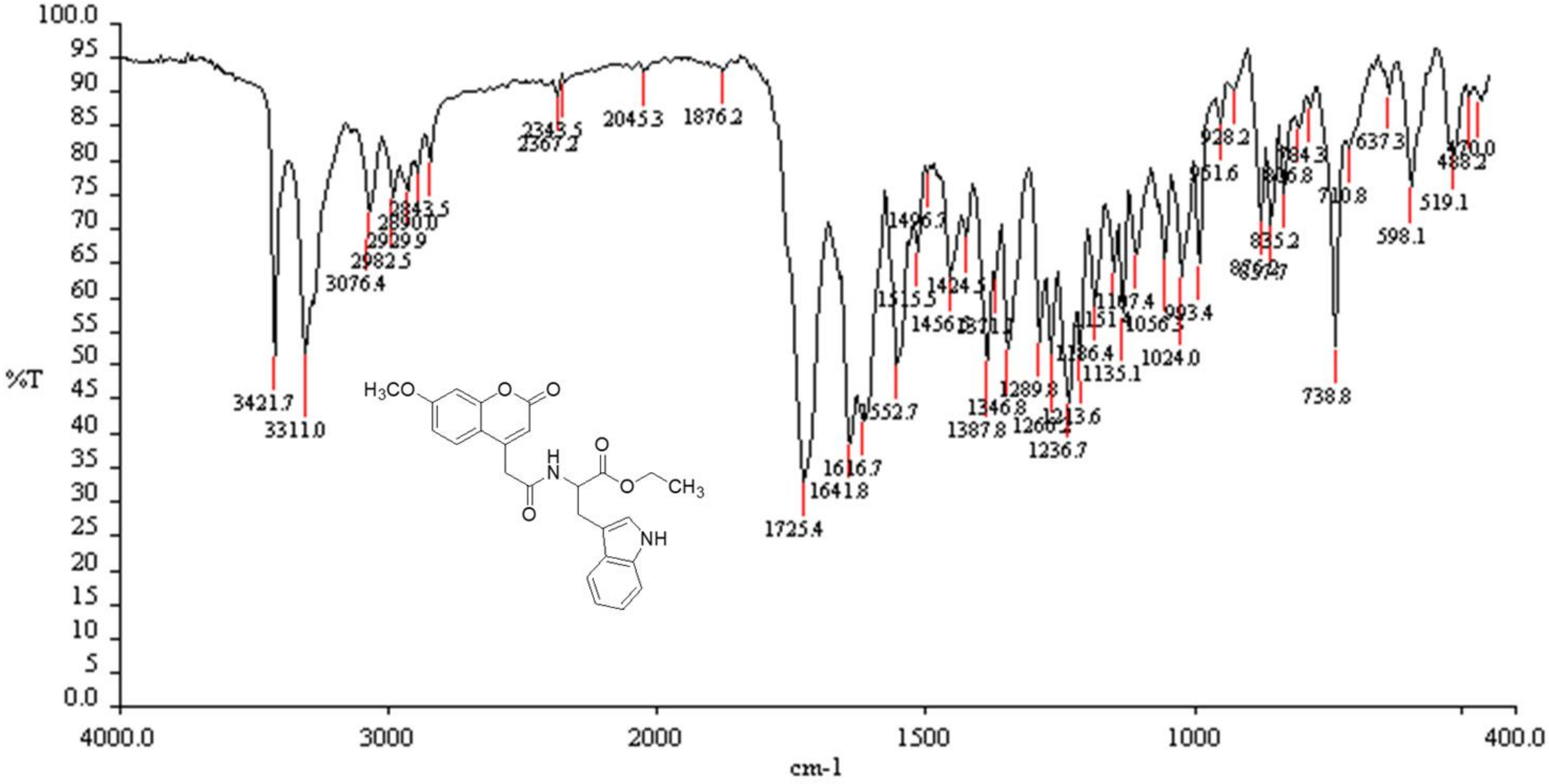


**Figure S-27** IR Spectra of Ethyl 3-(1H-indol-2-yl)-2-(2-(7-methoxy-2-oxo-2H-chromen-4-yl) acetamido) propanoate (**13**)

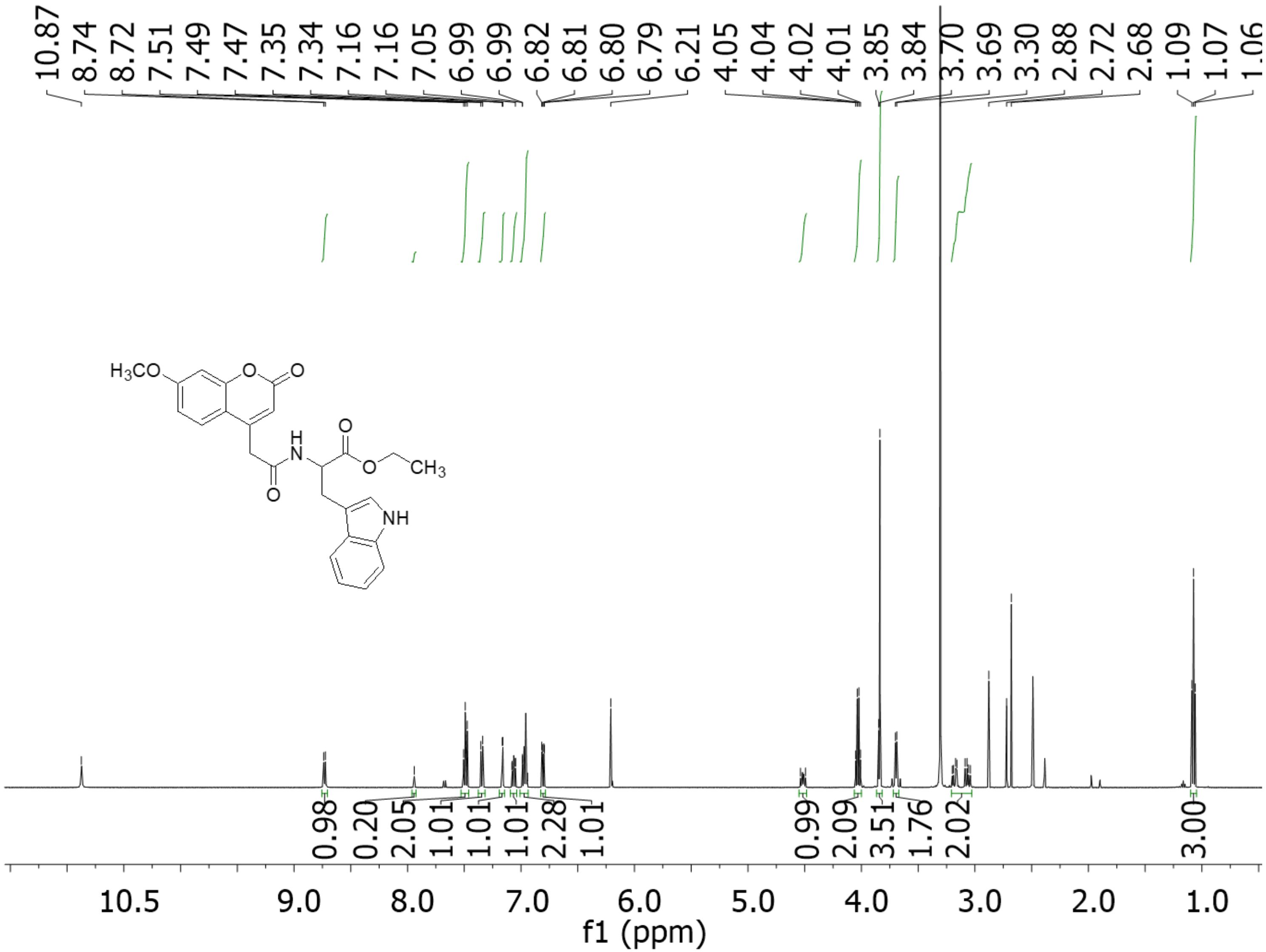


**Figure S-28** $^{1}$H-NMR spectra of Ethyl 3-(1H-indol-2-yl)-2-(2-(7-methoxy-2-oxo-2H-chromen-4-yl) acetamido) propanoate (**13**) in DMSO-$d_6$

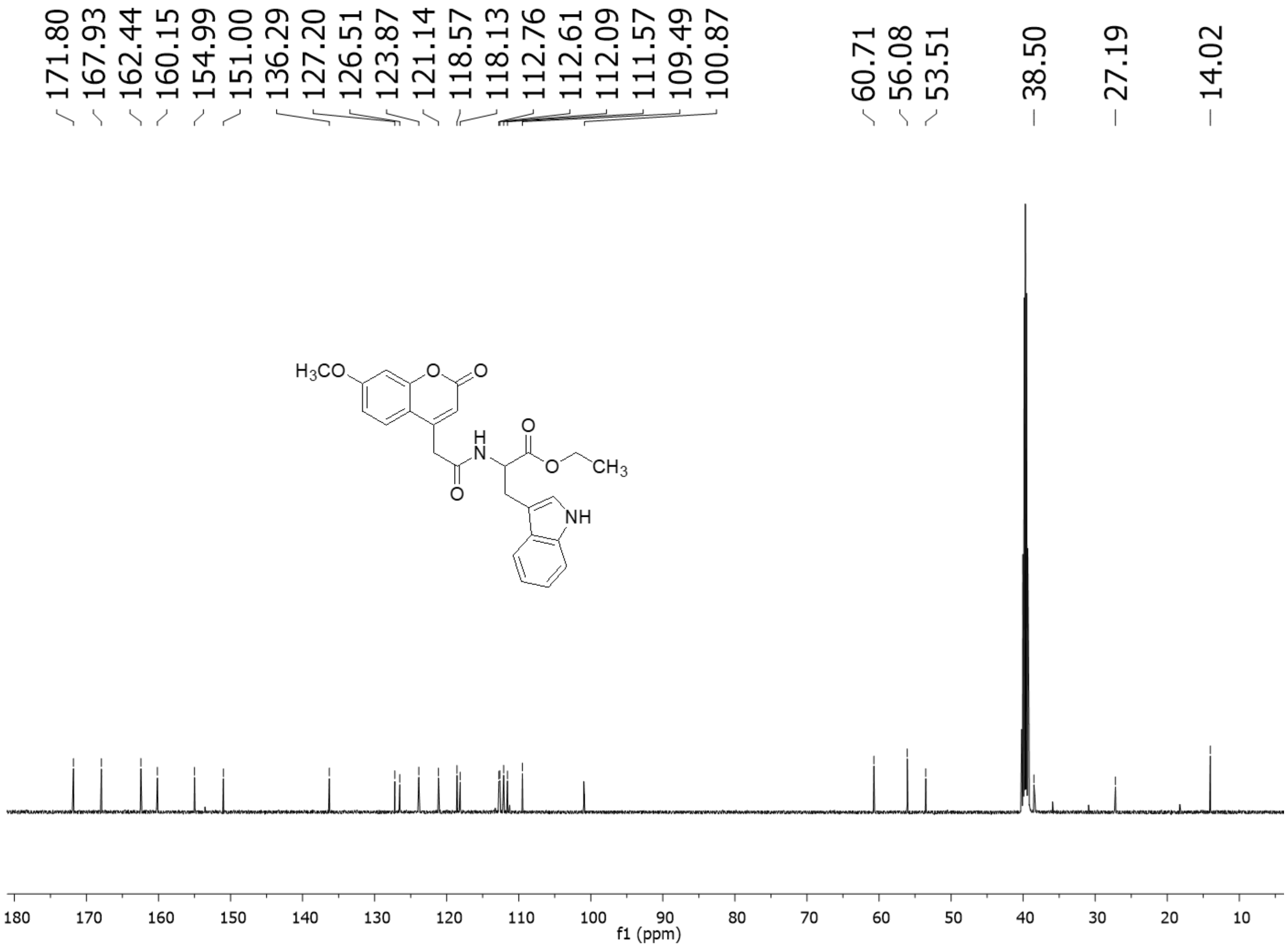


**Figure S-29** $^{13}$C-NMR spectra of Ethyl 3-(1H-indol-2-yl)-2-(2-(7-methoxy-2-oxo-2H-chromen-4-yl) acetamido) propanoate (**13**) in DMSO-$d_6$

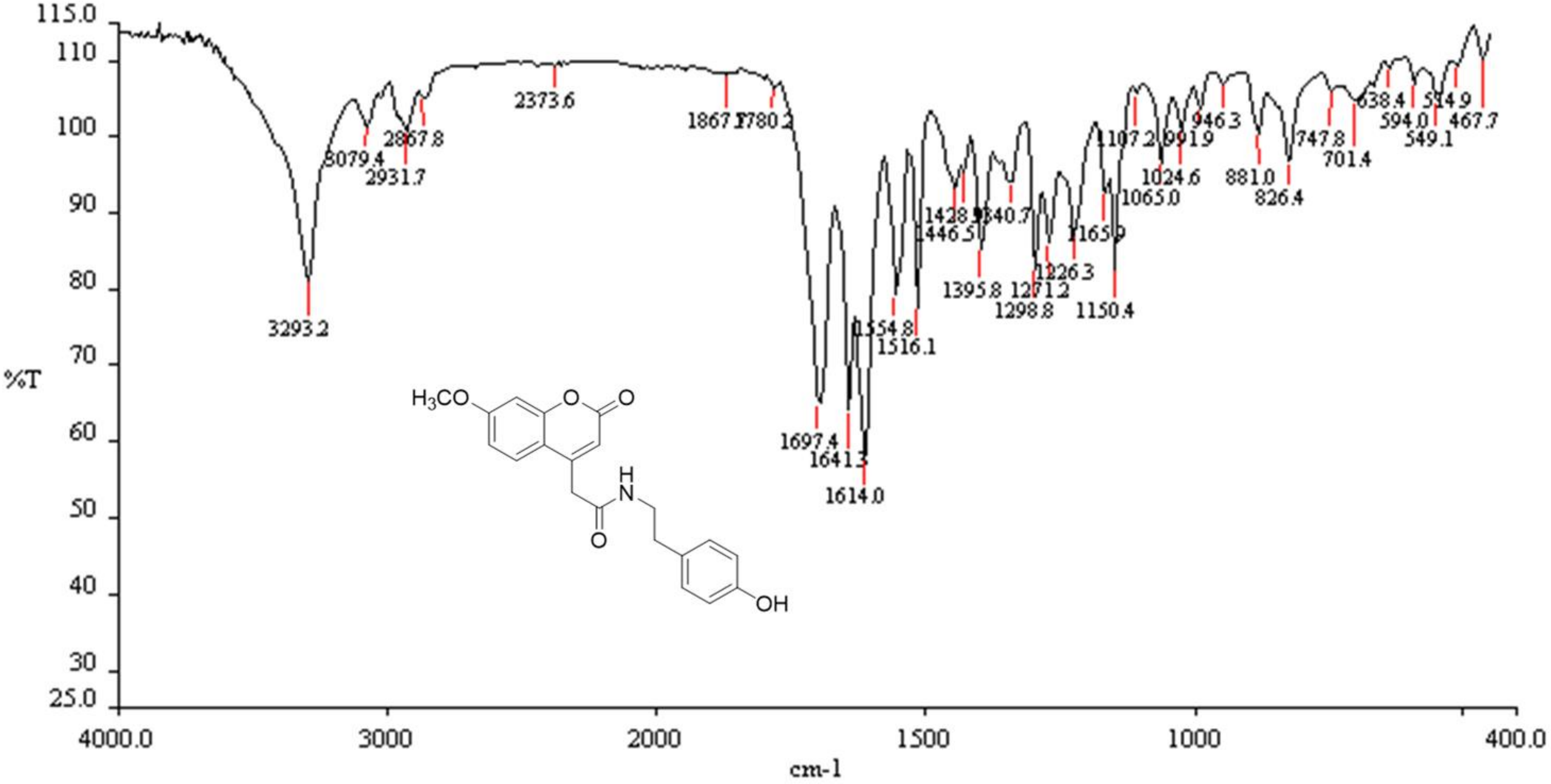


**Figure S-30** IR Spectra of N-(4-hydroxyphenethyl)-2-(7-methoxy-2-oxo-2H-chromen-4-yl)acetamide (**14**)

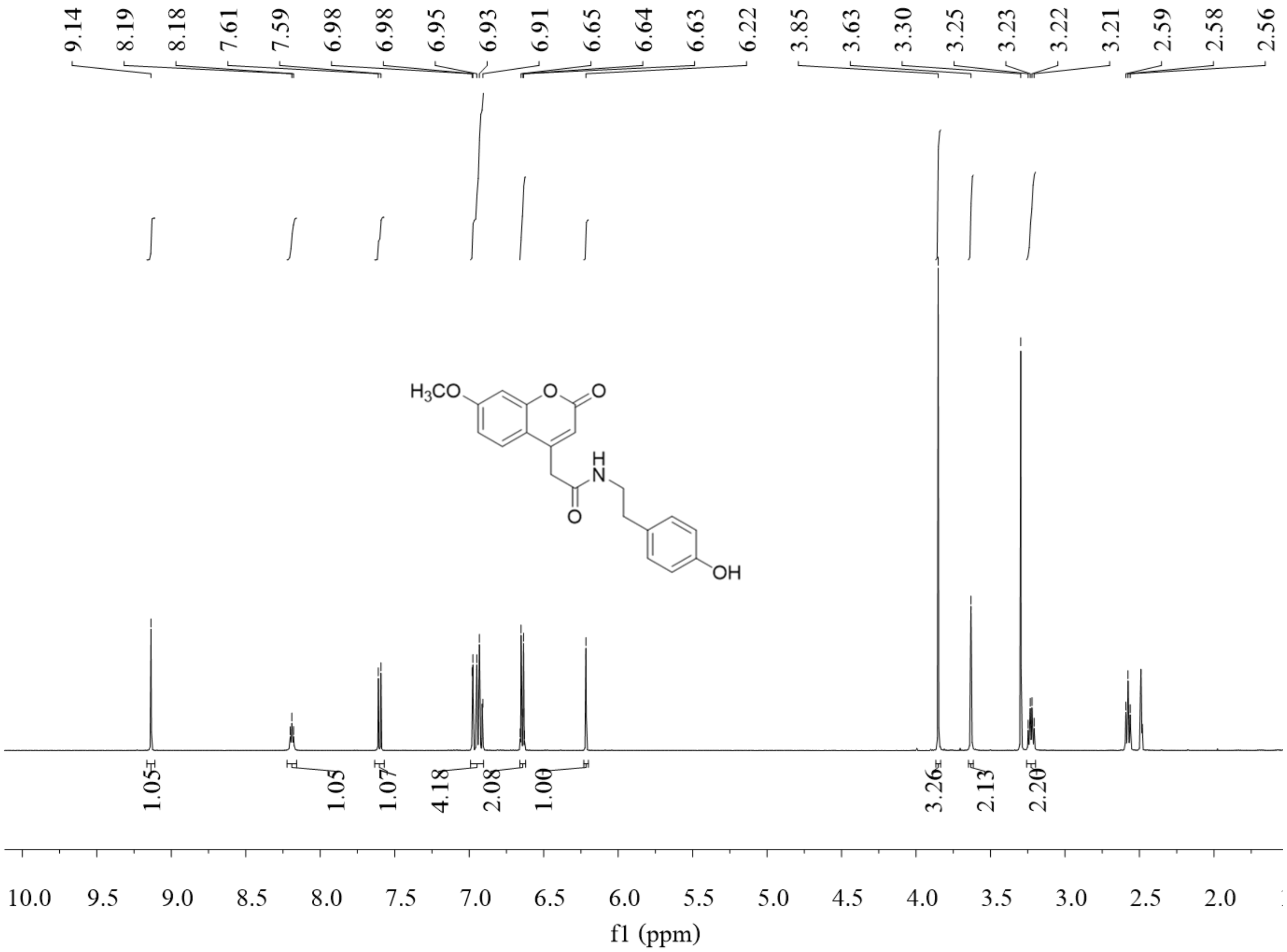


**Figure S-31** $^1$H-NMR spectra of N-(4-hydroxyphenethyl)-2-(7-methoxy-2-oxo-2H-chromen-4-yl)acetamide (**14**) in DMSO-$d_6$

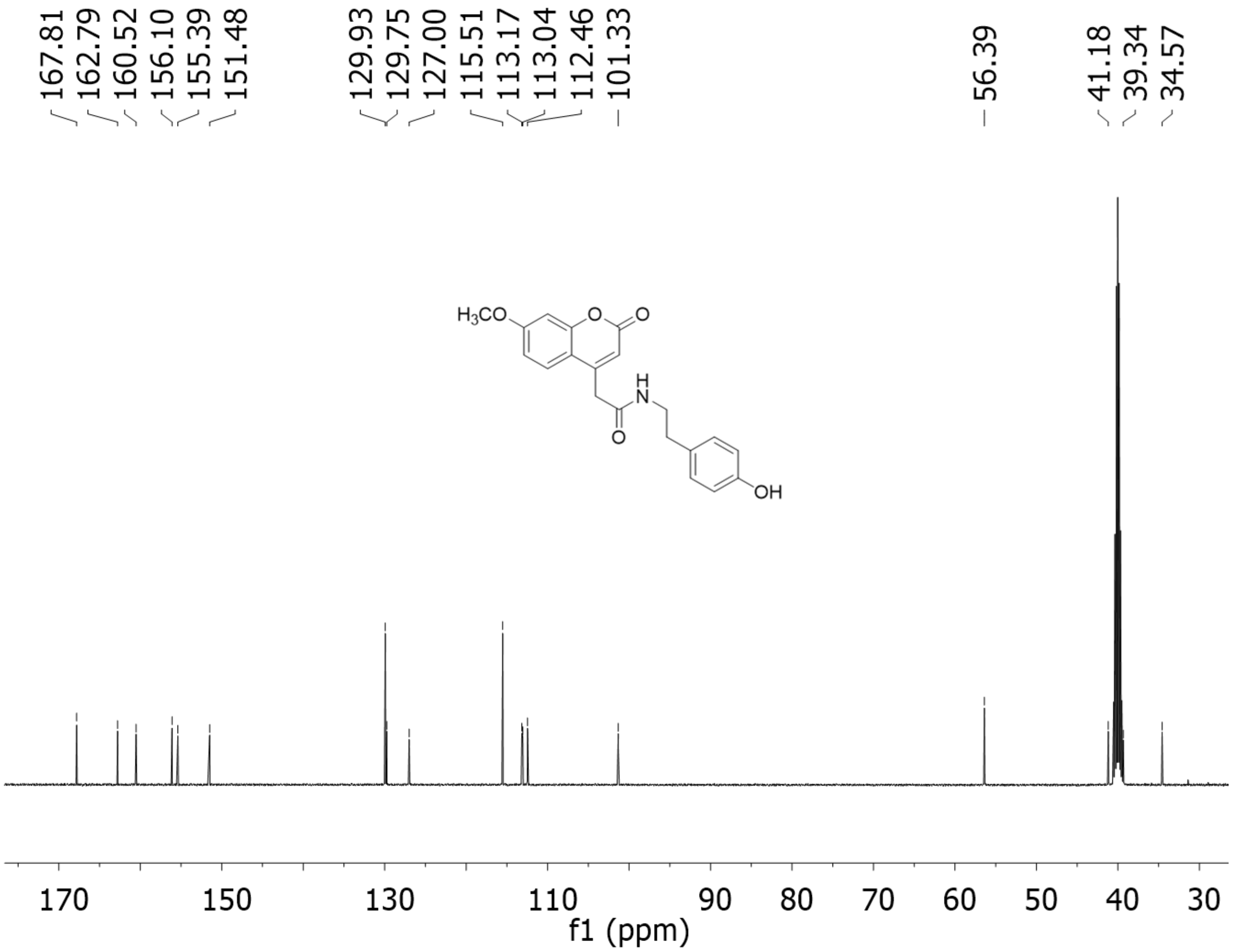


**Figure S-32** $^{13}$C-NMR spectra of N-(4-hydroxyphenethyl)-2-(7-methoxy-2-oxo-2H-chromen-4-yl)acetamide (**14**) in DMSO-$d_6$

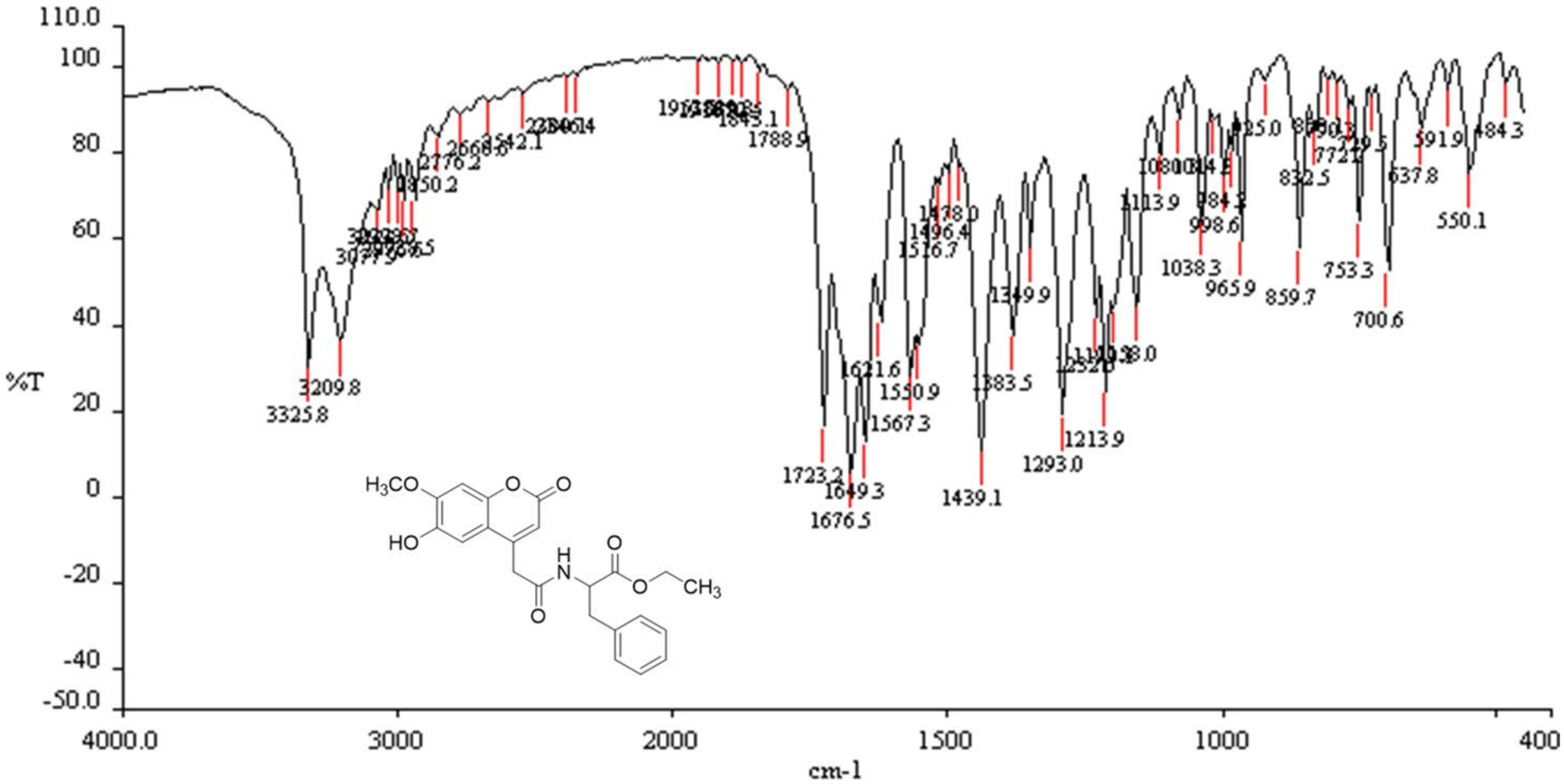


**Figure S-33** IR Spectra of Ethyl 2-(2-(6-hydroxy-7-methoxy-2-oxo-2H-chromen-4-yl)acetamido)-3-phenylpropanoate (**15**)

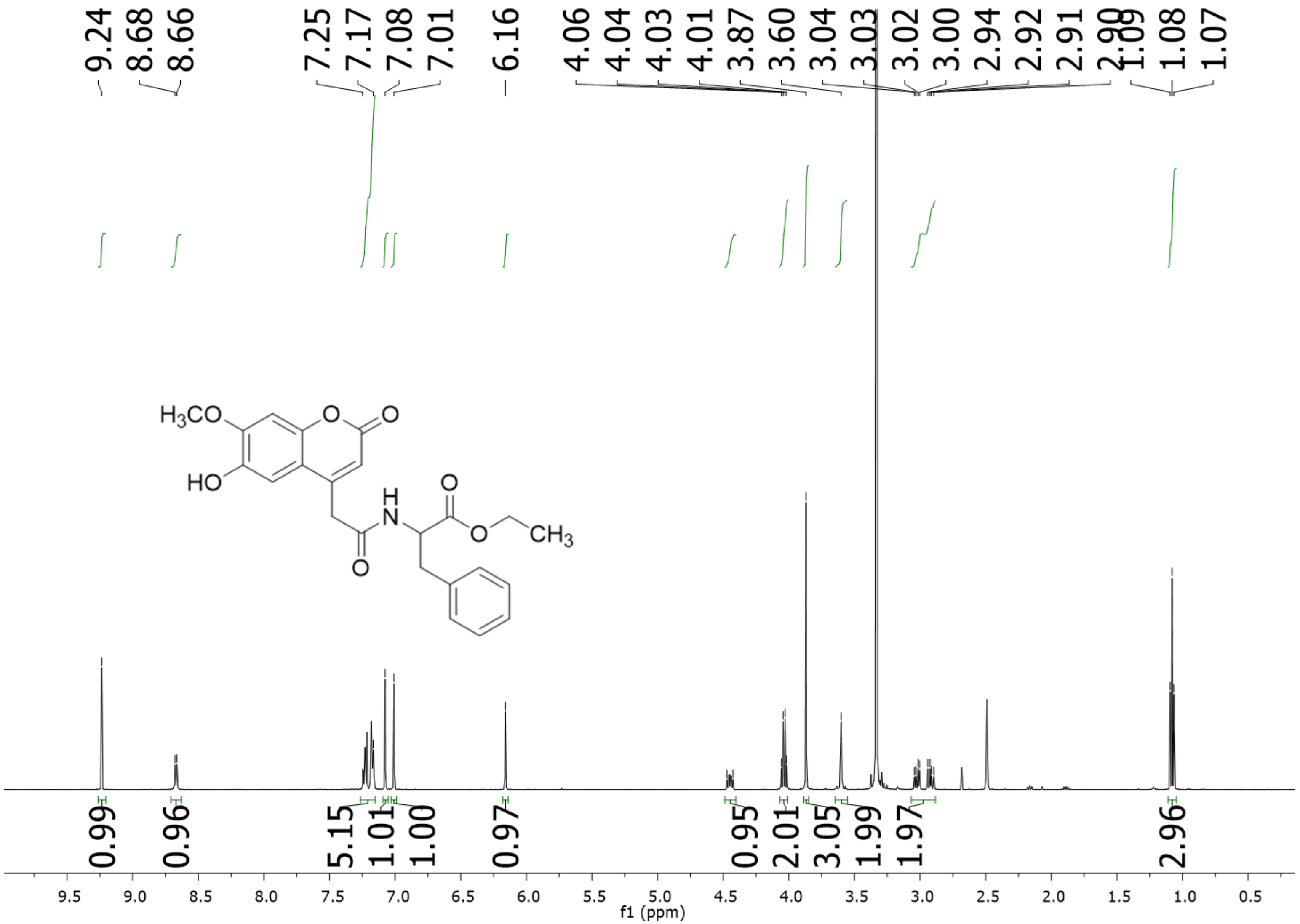

**Figure S-34** $^{1}$H-NMR spectra of Ethyl 2-(2-(6-hydroxy-7-methoxy-2-oxo-2H-chromen-4-yl)acetamido)-3-phenylpropanoate (**15**) in DMSO-$d_6$

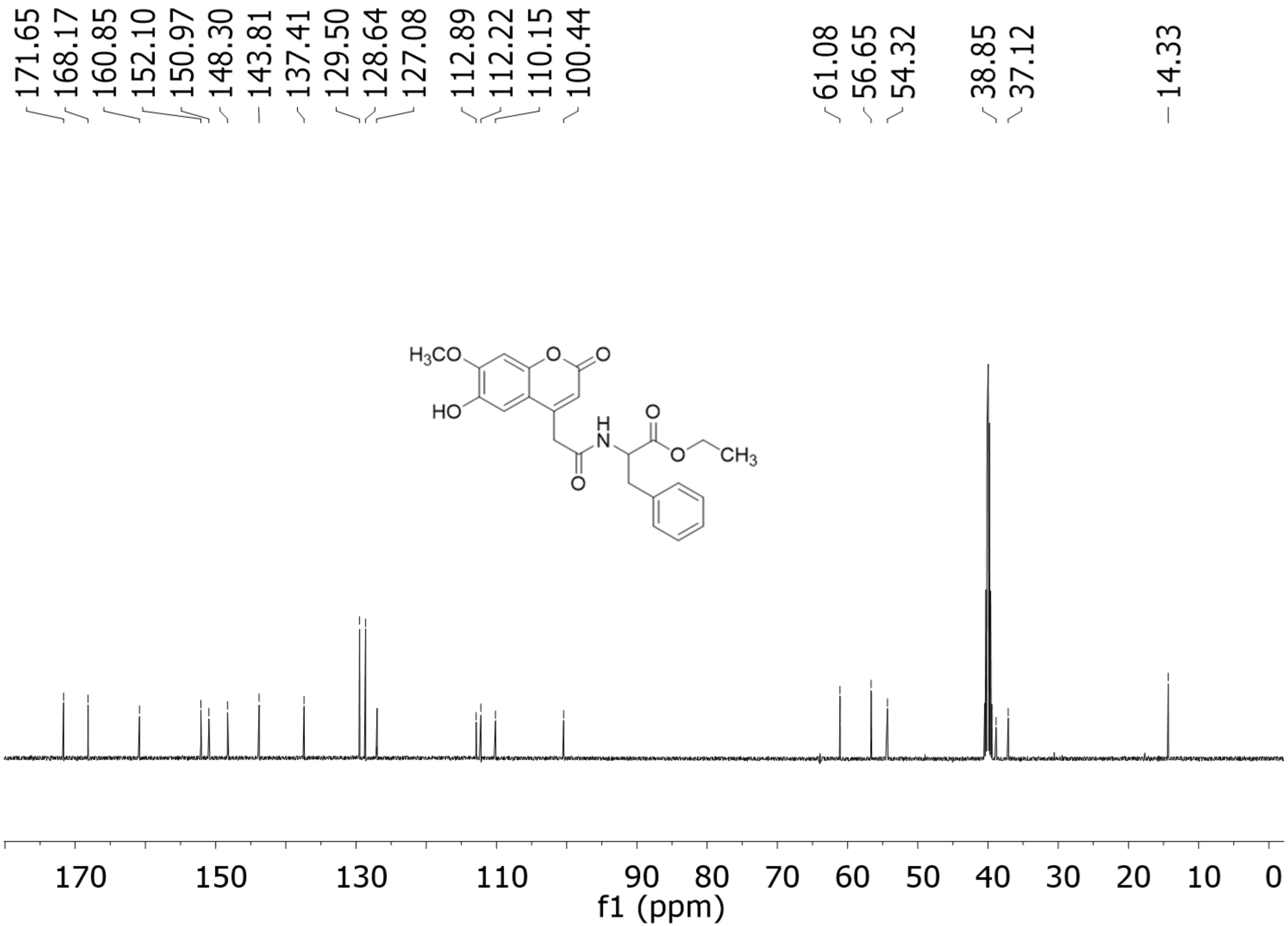


**Figure S-35** $^{13}$C-NMR spectra of Ethyl 2-(2-(6-hydroxy-7-methoxy-2-oxo-2H-chromen-4-yl)acetamido)-3-phenylpropanoate (**15**) in DMSO-$d_6$

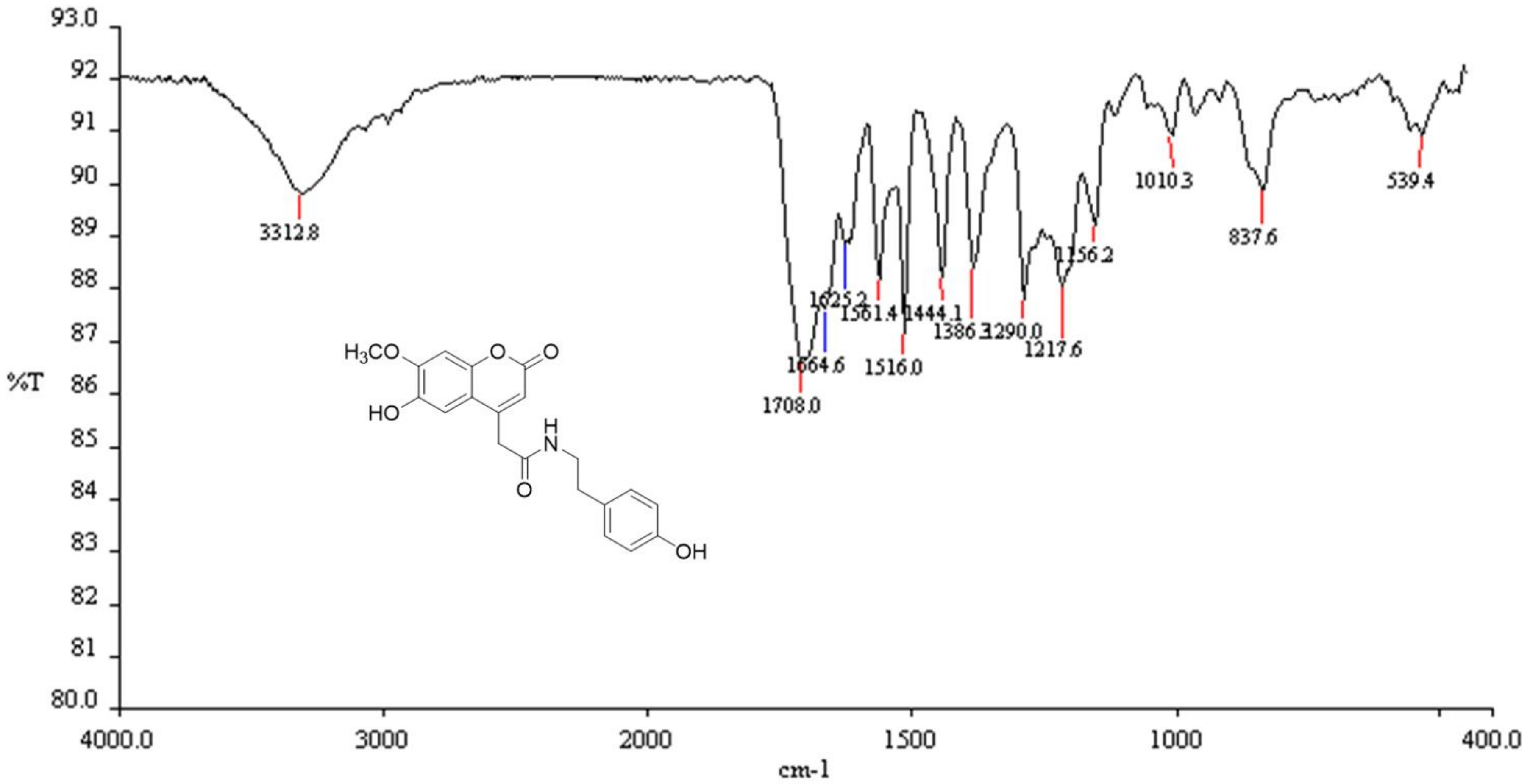


**Figure S-36** IR Spectra of Ethyl 2-(2-(6-hydroxy-7-methoxy-2-oxo-2H-chromen-4-yl)acetamido)-3-(4-hydroxyphenyl) propanoate (**16**)

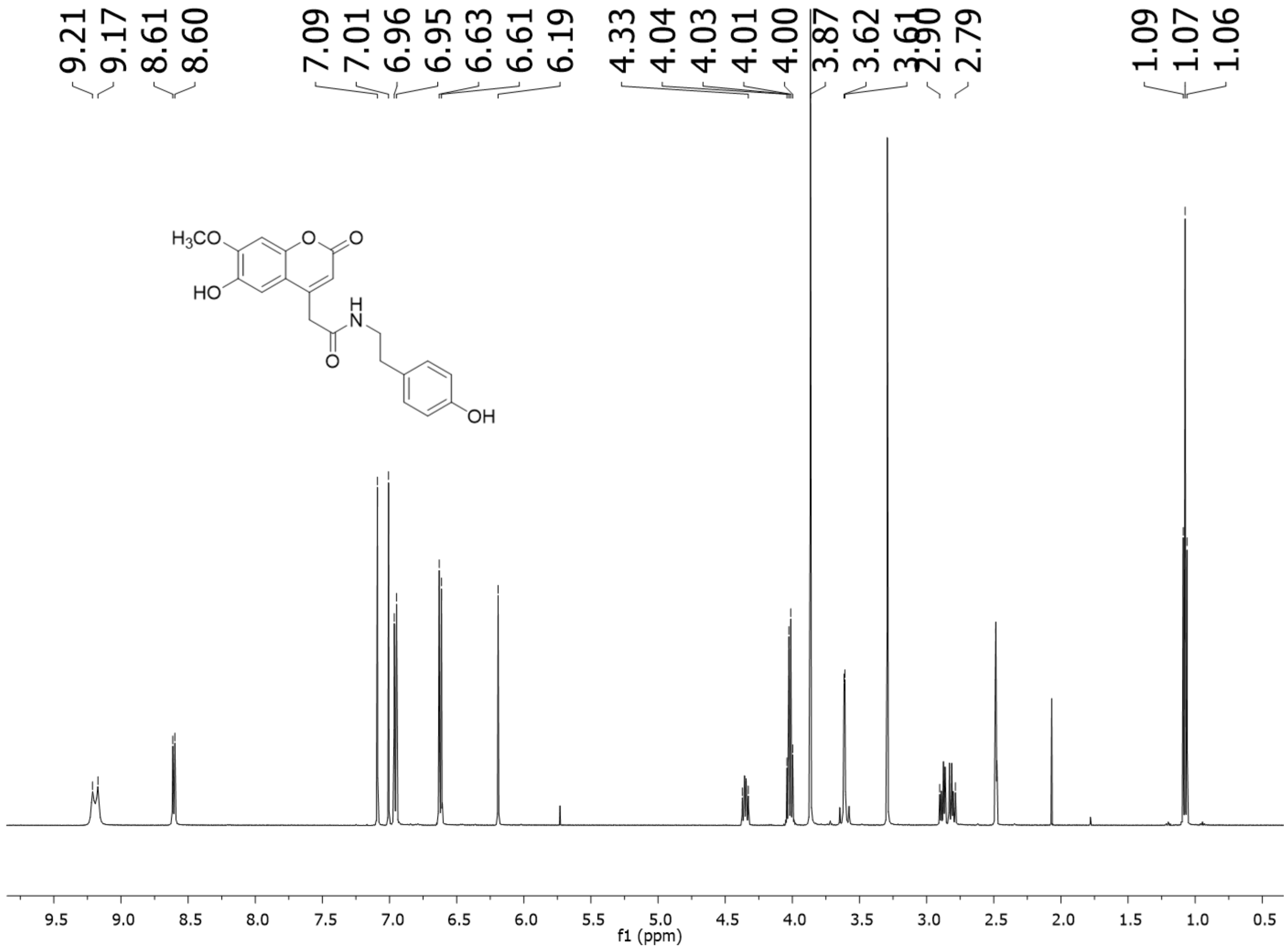


**Figure S-37** $^{1}$H-NMR spectra of Ethyl 2-(2-(6-hydroxy-7-methoxy-2-oxo-2H-chromen-4-yl)acetamido)-3-(4-hydroxyphenyl) propanoate (**16**) in DMSO-$d_6$

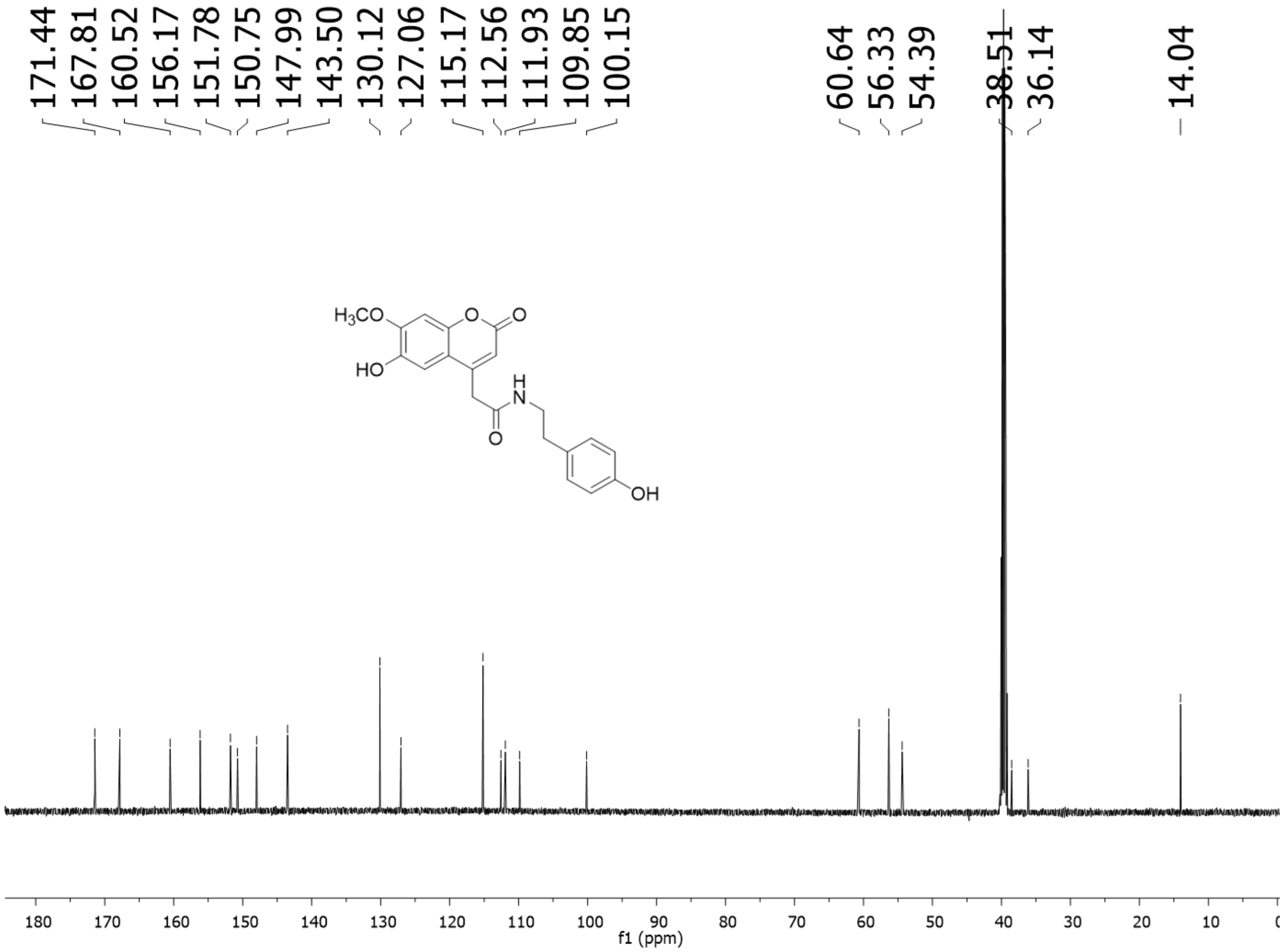


**Figure S-38** $^{13}$C-NMR spectra of Ethyl 2-(2-(6-hydroxy-7-methoxy-2-oxo-2H-chromen-4-yl)acetamido)-3-(4-hydroxyphenyl) propanoate (**16**) in DMSO-$d_6$

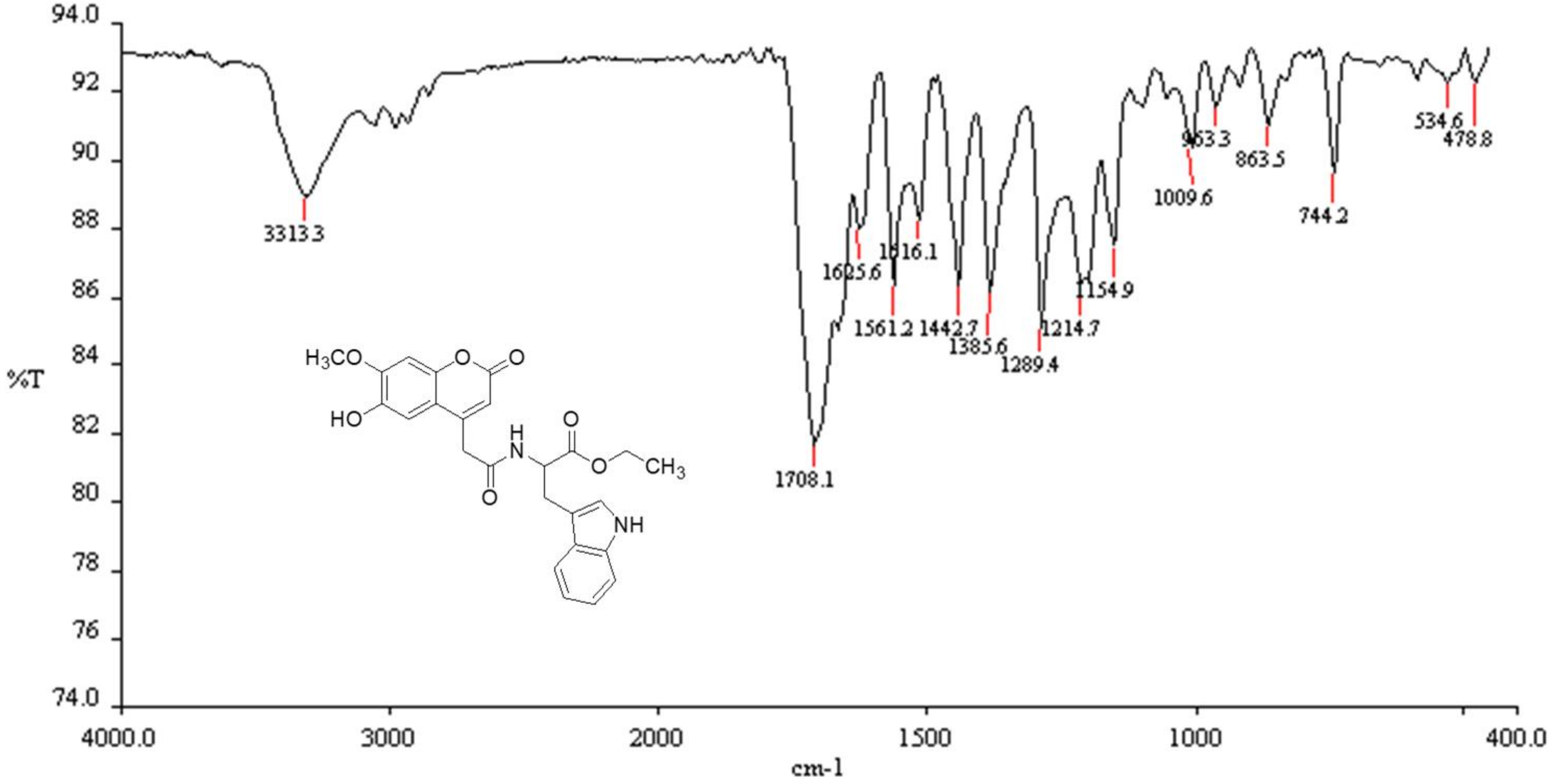


**Figure S-39** IR Spectra of Ethyl 3-(1H-indol-2-yl)-2-(2-(6-hydroxy-7-methoxy-2-oxo-2H-chromen-4-yl) acetamido) propanoate (**17**)

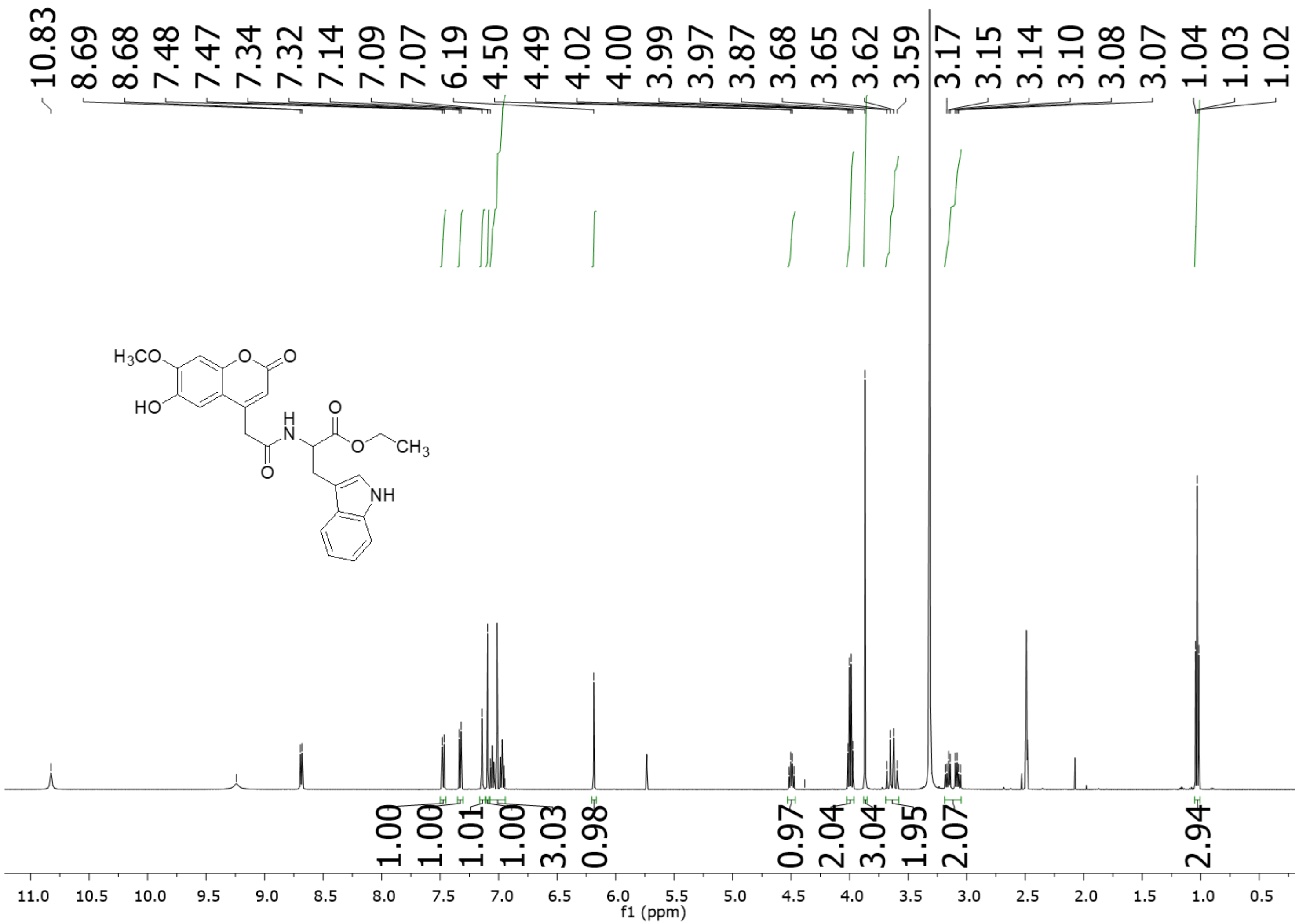


**Figure S-40** $^{1}$H-NMR spectra of Ethyl 3-(1H-indol-2-yl)-2-(2-(6-hydroxy-7-methoxy-2-oxo-2H-chromen-4-yl) acetamido) propanoate (**17**) in DMSO-$d_6$

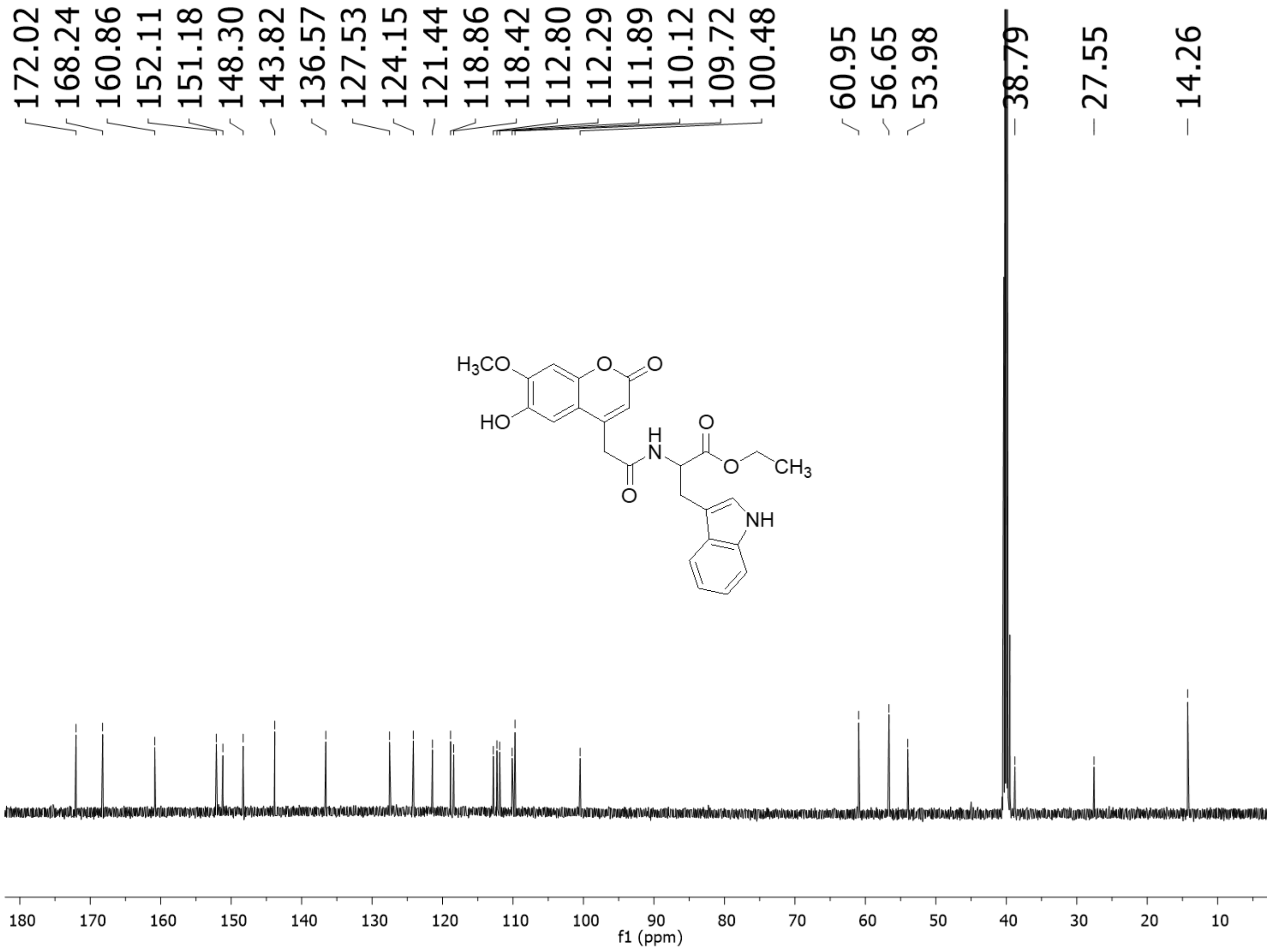


**Figure S-41** $^{13}$C-NMR spectra of Ethyl 3-(1H-indol-2-yl)-2-(2-(6-hydroxy-7-methoxy-2-oxo-2H-chromen-4-yl) acetamido) propanoate (**17**) in DMSO-$d_6$

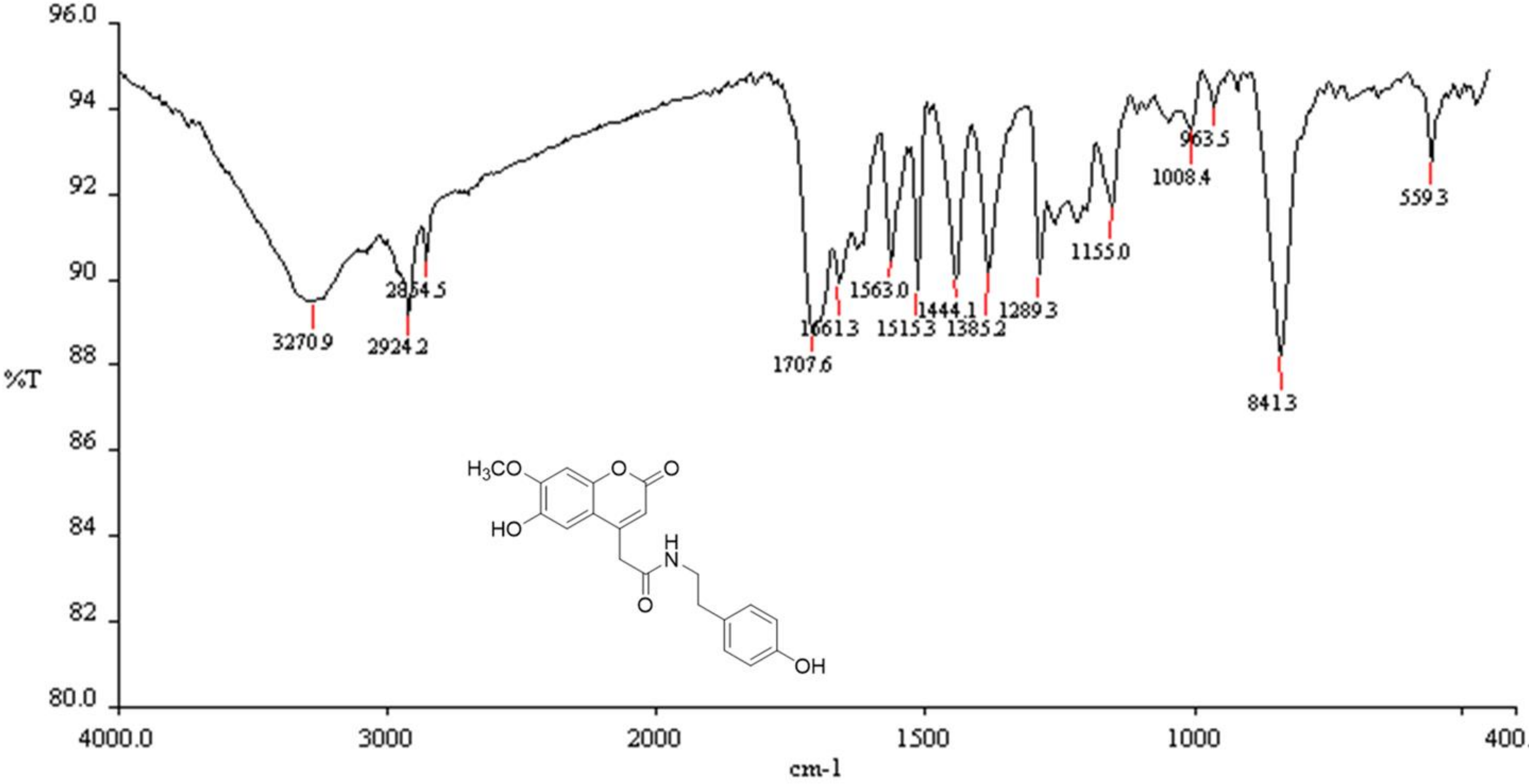


**Figure S-42** IR Spectra of N-(4-hydroxyphenethyl)-2-(6-hydroxy-7-methoxy-2-oxo-2H-chromen-4-yl)acetamide (**18**)

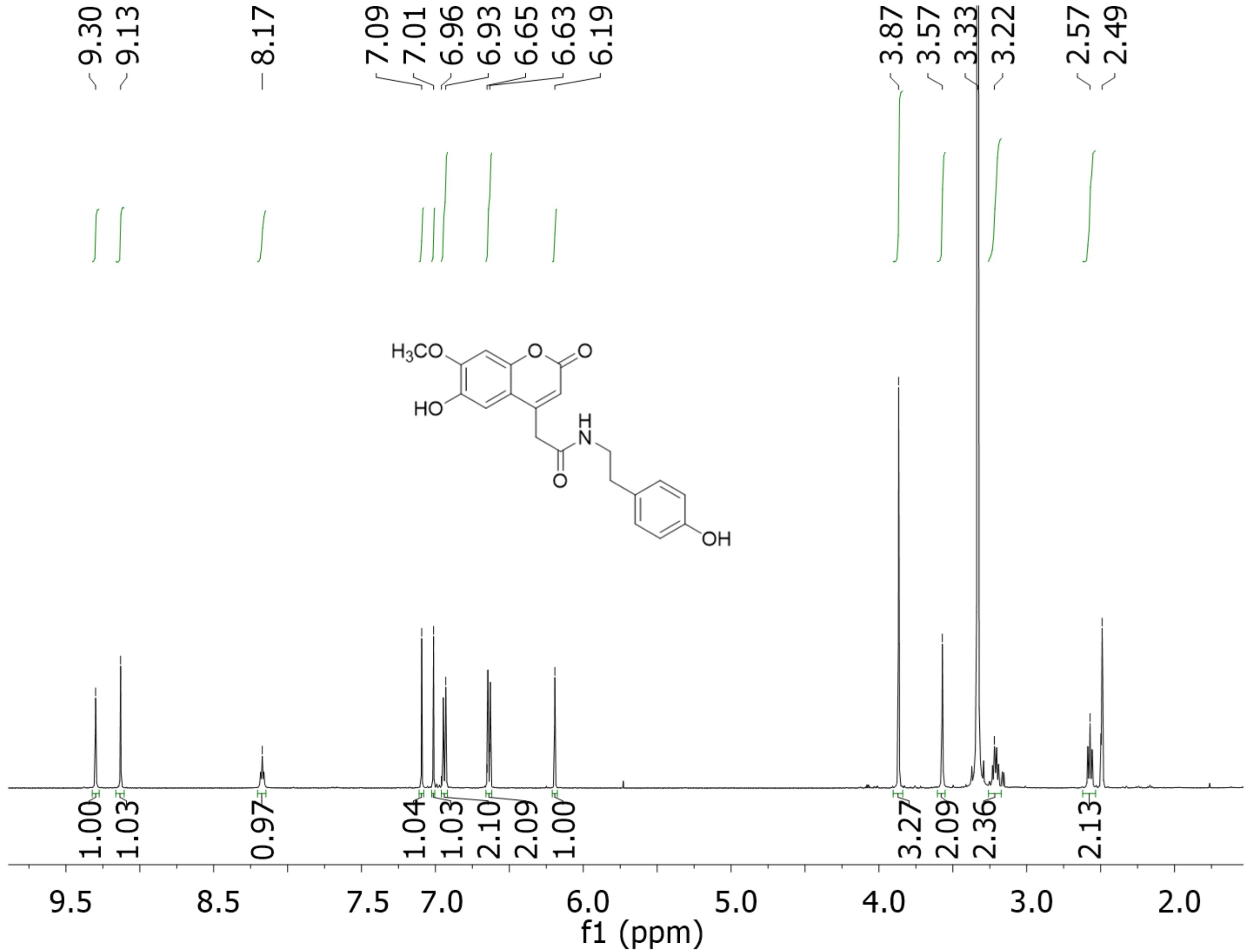


**Figure S-43** $^{1}$H-NMR spectra of N-(4-hydroxyphenethyl)-2-(6-hydroxy-7-methoxy-2-oxo-2H-chromen-4-yl)acetamide (**18**) in DMSO-$d_6$

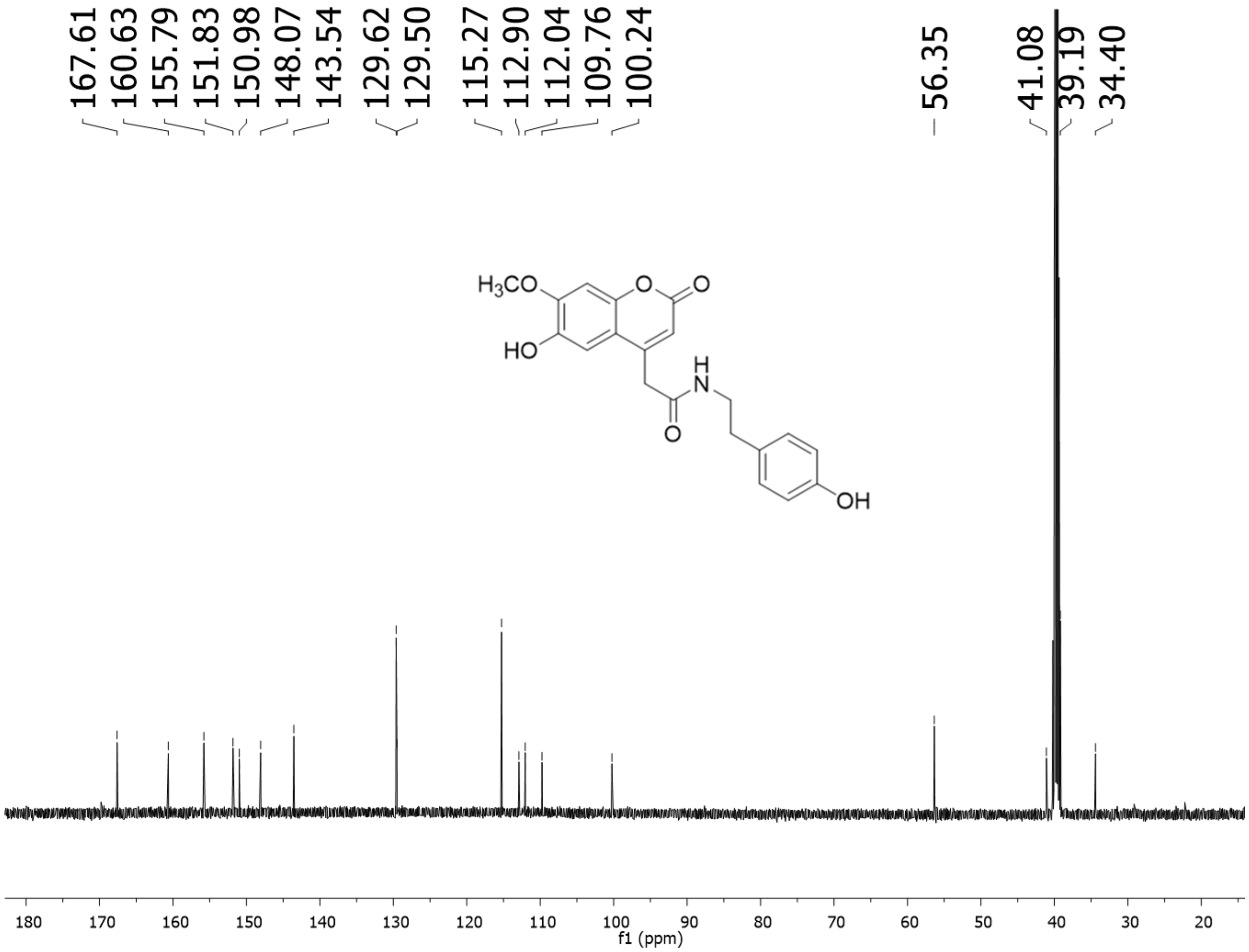


**Figure S-44** $^{13}$C-NMR spectra of N-(4-hydroxyphenethyl)-2-(6-hydroxy-7-methoxy-2-oxo-2H-chromen-4-yl)acetamide (**18**) in DMSO-$d_6$

**Percent purity of the synthesized compounds**

The purity of the final coumarin derivatives was confirmed by HPLC analysis and was found >95% purity as shown below.

**Chromatographic condition**

- HPLC HITACHI Chromaster_5110 Pump, 5210 Auto Sampler, 5310 column oven, 5430 Diode Array Detector
- Column BDS Hypersil C18 Size: 250 X 4.6 mm Brand: Thermo Scientific

**System 1:** Mobile phase MeOH:2%acetic acid (45:55), flow rate 1 (mL/min) and UV detection at 254 nm

**System 2:** Mobile phase MeOH:2%acetic acid (55:45), flow rate 1 (mL/min) and UV detection at 254 nm

**Table S-1 Percent purity of the synthesized compounds**

| **Cpd** | **HR-MS (EI)** | **System 1** | | **System 2** | |
|---|---|---|---|---|---|
| | | Purity (%) | $t_R$ (min) | Purity (%) | $t_R$ (min) |
| **4** | Calculated mass 220.1782 | 100.00 | 3.727 | - | - |
| | Measured mass (m/z, $(M+1)^+$) 221.0457 | | | | |
| **5** | Calculated mass 234.2048 | 100.00 | 4.807 | - | - |
| | Measured mass (m/z, $(M+1)^+$) 235.0616 | | | | |
| **6** | Calculated mass 250.2042 | 98.76 | 3.647 | - | - |
| | Measured mass (m/z, $(M+1)^+$) 251.0565 | | | | |
| **7** | Calculated mass 395.4052 | - | - | 97.96 | 5.200 |
| | Measured mass (m/z, $(M+1)^+$) 396.1437 | | | | |
| **8** | Calculated mass 411.4046 | - | - | 97.64 | 3.820 |
| | Measured mass (m/z, $(M+1)^+$) 412.1391 | | | | |
| **9** | Calculated mass 434.4413 | - | - | 96.50 | 6.870 |
| | Measured mass (m/z, $(M+1)^+$) 435.1383 | | | | |

| Cpd | HR-MS (EI) | System 1 | | System 2 | |
|---|---|---|---|---|---|
| | | Purity (%) | $t_R$ (min) | Purity (%) | $t_R$ (min) |
| **10** | Calculated mass 339.3420 | - | - | 100.00 | 3.453 |
| | Measured mass (m/z, $(M+1)^+$) 340.1195 | | | | |
| **11** | Calculated mass 409.4318 | - | - | 99.40 | 7.387 |
| | Measured mass (m/z, $(M+1)^+$) 410.1593 | | | | |
| **12** | Calculated mass 425.4312 | - | - | 98.64 | 4.567 |
| | Measured mass (m/z, $(M+1)^+$) 426.1539 | | | | |
| **13** | Calculated mass 448.4679 | - | - | 95.73 | 6.860 |
| | Measured mass (m/z, $(M+1)^+$) 449.1704 | | | | |
| **14** | Calculated mass 353.3686 | - | - | 99.94 | 11.740 |
| | Measured mass (m/z, $(M+1)^+$) 354.1325 | | | | |
| **15** | Calculated mass 425.4312 | - | - | 98.79 | 4.940 |
| | Measured mass (m/z, $(M+1)^+$) 426.1537 | | | | |
| **16** | Calculated mass 441.1424 | - | - | 100.00 | 3.707 |
| | Measured mass (m/z, $(M+1)^+$) 442.1496 | | | | |
| **17** | Calculated mass 464.4673 | - | - | 97.42 | 4.840 |
| | Measured mass (m/z, $(M+1)^+$) 465.1648 | | | | |
| **18** | Calculated mass 369.3680 | - | - | 100.00 | 3.480 |
| | Measured mass (m/z, $(M+1)^+$) 370.1275 | | | | |

## Chromatograms

Compound **4**

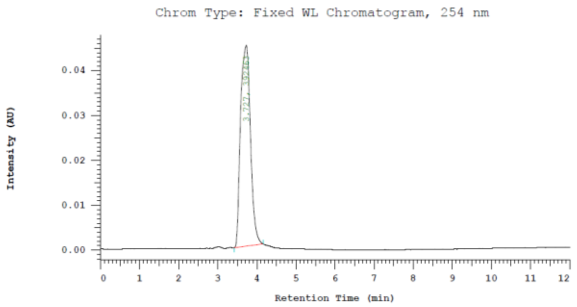


| No. | RT | Area | Conc 1 | BC |
|---|---|---|---|---|
| 1 | 3.727 | 392463 | 0.000000 | BB |
| | | 392463 | 0.000000 | |

Compound **5**

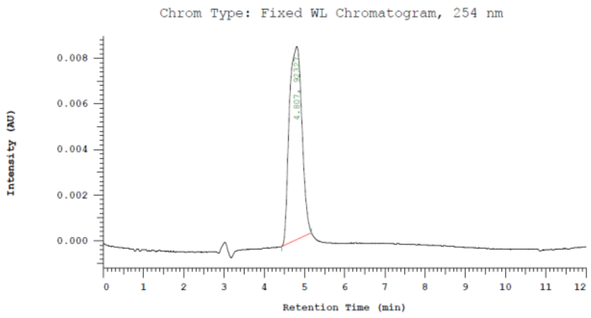


| No. | RT | Area | Conc 1 | BC |
|---|---|---|---|---|
| 1 | 4.807 | 92327 | 0.000000 | BB |
| | | 92327 | 0.000000 | |

Compound **6**

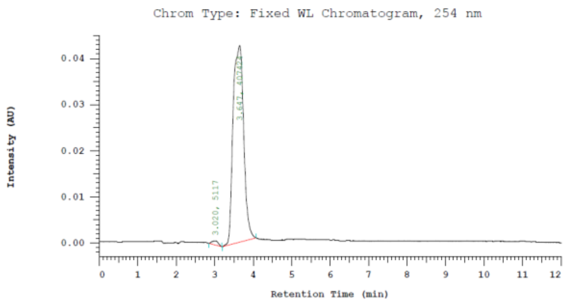


| No. | RT | Area | Conc 1 | BC |
|---|---|---|---|---|
| 1 | 3.020 | 5117 | 0.000000 | BB |
| 2 | 3.647 | 407424 | 0.000000 | BB |
| | | 412541 | 0.000000 | |

Compound **7**

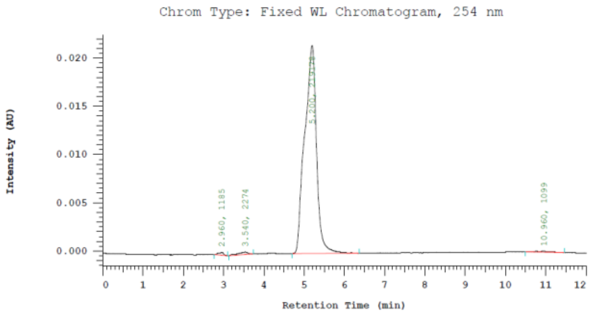


| No. | RT | Area | Conc 1 | BC |
|---|---|---|---|---|
| 1 | 2.960 | 1185 | 0.000000 | BB |
| 2 | 3.540 | 2274 | 0.000000 | BB |
| 3 | 5.200 | 219178 | 0.000000 | BB |
| 4 | 10.960 | 1099 | 0.000000 | BB |
| | | 223736 | 0.000000 | |

Compound **8**

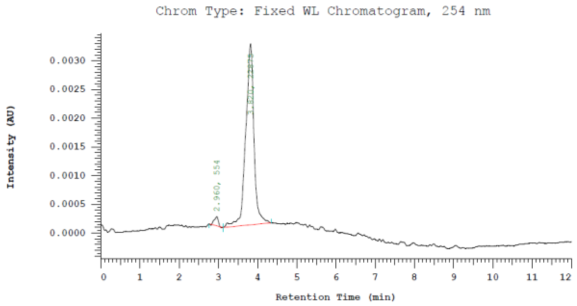


| No. | RT | Area | Conc 1 | BC |
|---|---|---|---|---|
| 1 | 2.960 | 554 | 0.000000 | BB |
| 2 | 3.820 | 22875 | 0.000000 | BB |
| | | 23429 | 0.000000 | |

Compound **9**

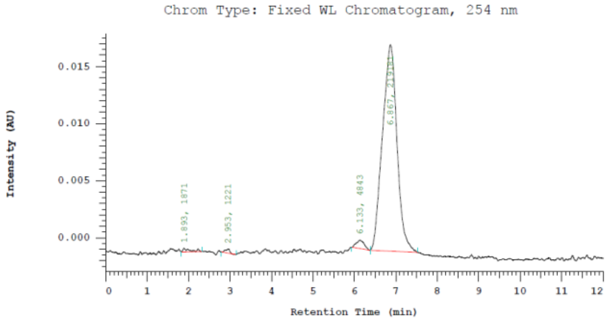


| No. | RT | Area | Conc 1 | BC |
|---|---|---|---|---|
| 1 | 1.893 | 1871 | 0.000000 | BB |
| 2 | 2.953 | 1221 | 0.000000 | BB |
| 3 | 6.133 | 4843 | 0.000000 | BB |
| 4 | 6.867 | 219181 | 0.000000 | BB |
| | | 227116 | 0.000000 | |

Compound **10**

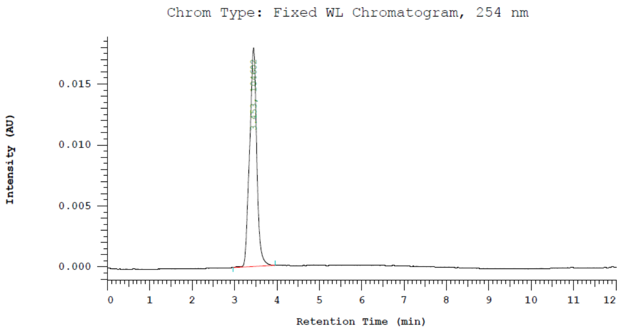


| No. | RT | Area | Conc 1 | BC |
|---|---|---|---|---|
| 1 | 3.453 | 104602 | 0.000000 | BB |
| | | 104602 | 0.000000 | |

Compound **11**

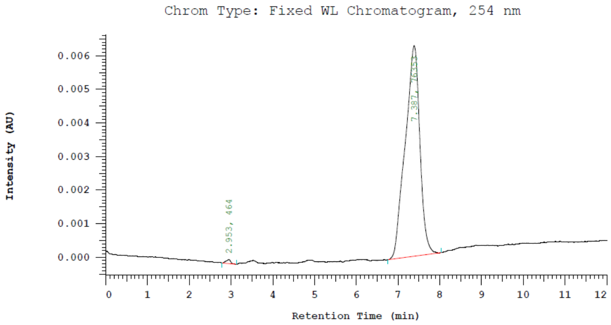


| No. | RT | Area | Conc 1 | BC |
|---|---|---|---|---|
| 1 | 2.953 | 464 | 0.000000 | BB |
| 2 | 7.387 | 76353 | 149.625 | BB |
| | | 76817 | 149.625 | |

Compound **12**

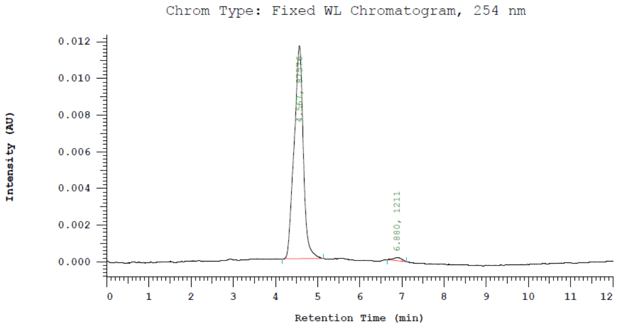


| No. | RT | Area | Conc 1 | BC |
|---|---|---|---|---|
| 1 | 4.567 | 87576 | 0.000000 | BB |
| 2 | 6.880 | 1211 | 0.000000 | BB |
| | | 88787 | 0.000000 | |

Compound **13**

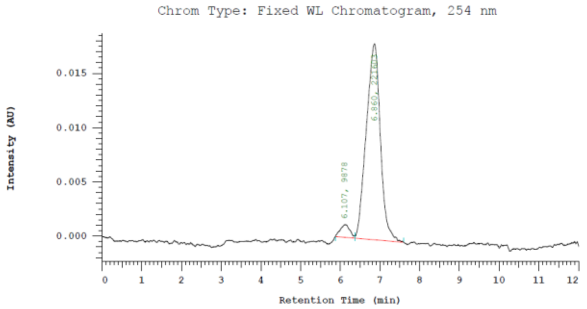


| No. | RT | Area | Conc 1 | BC |
|---|---|---|---|---|
| 1 | 6.107 | 9878 | 0.000000 | BB |
| 2 | 6.860 | 221603 | 0.000000 | BB |
| | | 231481 | 0.000000 | |

Compound **14**

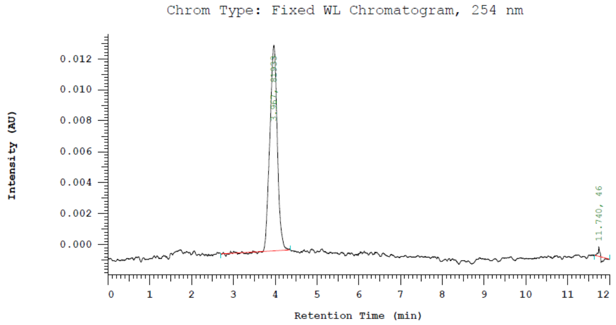


| No. | RT | Area | Conc 1 | BC |
|---|---|---|---|---|
| 1 | 3.967 | 81933 | 0.000000 | BB |
| 2 | 11.740 | 46 | 0.000000 | BB |
| | | 81979 | 0.000000 | |

Compound **15**

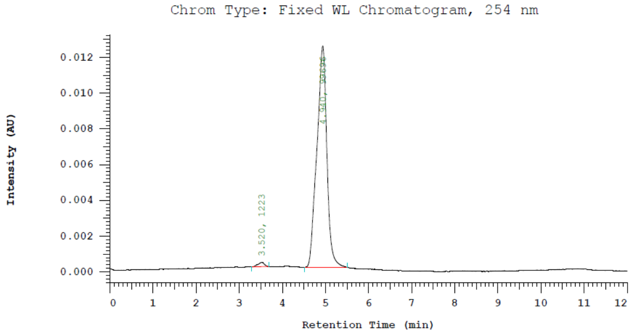


| No. | RT | Area | Conc 1 | BC |
|---|---|---|---|---|
| 1 | 3.520 | 1223 | 0.000000 | BB |
| 2 | 4.940 | 99696 | 0.000000 | BB |
| | | 100919 | 0.000000 | |

Compound **16**

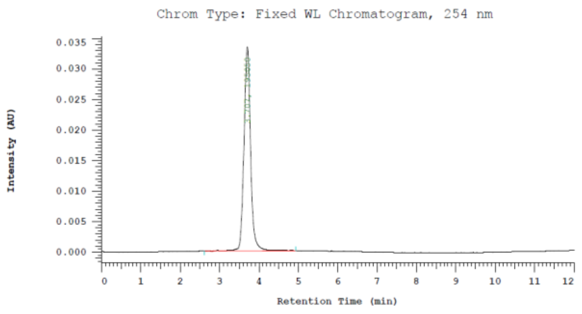


| No. | RT | Area | Conc 1 | BC |
|---|---|---|---|---|
| 1 | 3.707 | 195050 | 0.000000 | BB |
| | | 195050 | 0.000000 | |

Compound **17**

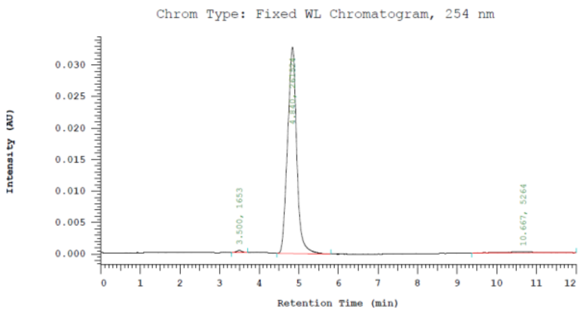


| No. | RT | Area | Conc 1 | BC |
|---|---|---|---|---|
| 1 | 3.500 | 1653 | 0.000000 | BB |
| 2 | 4.840 | 261524 | 0.000000 | BB |
| 3 | 10.667 | 5264 | 0.000000 | BB |
| | | 268441 | 0.000000 | |

Compound **18**

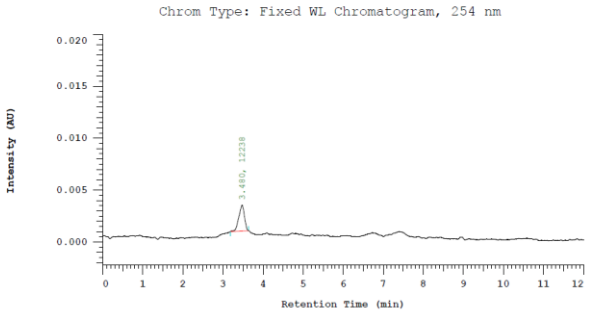


| No. | RT | Area | Conc 1 | BC |
|---|---|---|---|---|
| 1 | 3.480 | 12238 | 0.000000 | BB |
| | | 12238 | 0.000000 | |
